# Synthesis and Evaluation of Antimicrobial Activity of the Rearranged Abietane Prattinin A and Its Synthetic Derivatives

**DOI:** 10.3390/molecules29030650

**Published:** 2024-01-30

**Authors:** Mustapha Ait El Had, Manal Zefzoufi, Houda Zentar, Lahoucine Bahsis, Mouhi Eddine Hachim, Adib Ghaleb, Choukri Khelifa-Mahdjoubi, Hafida Bouamama, Ramón Alvarez-Manzaneda, José Justicia, Rachid Chahboun

**Affiliations:** 1 Departamento de Química Organica, Facultad de Ciencias, Universidad de Granada, 18071 Granada, Spain; mustapha.aitelhad20@gmail.com (M.A.E.H.); choukri_khelifa@hotmail.com (C.K.-M.); 2 Département des Sciences Fondamentales, Faculté de Médecine et de Pharmacie, et de Médecine Dentaire de Fès, Université Sidi Mohamed Ben Abdellah de Fès, Fes 30000, Morocco; 3 Recherche en Développement Durable et Santé, Faculté des Sciences et Techniques, Cadi Ayyad University, Marrakech 40000, Morocco; zefzoufi.manal1994@gmail.co (M.Z.); bouamamahafida@gmail.com (H.B.); 4 Laboratory of Analytical and Molecular Chemistry, Polydisciplinary Faculty, Cadi Ayyad University, BP 4162, Safi 46000, Morocco; bahsis.lahoucine@gmail.com (L.B.); mouhieddinehachim@gmail.com (M.E.H.); adib.ghaleb@gmail.com (A.G.); 5 Área de Química Orgánica, Departamento de Química y Física, Universidad de Almería, 04120 Almería, Spain; ralvarez@ual.es

**Keywords:** natural product, abietane rearranged, prattinin A, antibacterial activity, ADMET, DFT calculation, molecular docking

## Abstract

Synthesis of the natural product prattinin A and some new derivatives has been achieved using abietic acid. The final products and a selection of intermediates were evaluated for their antibacterial activity against three human pathogenic bacteria: *E. coli*, *P. aeruginosa*, and *S. aureus*. The results showed that the antibacterial activity varies depending on the chemical structure of the compounds. Notably, compound **27** exhibited the most potent activity against *E. coli* and *P. aeruginosa,* with a minimal inhibitory concentration (MIC) of 11.7 µg/mL, comparable to that of the standard antibiotic ciprofloxacin, and strong activity against *S. aureus*, with an MIC of 23.4 µg/mL. Furthermore, we assessed the stability of these derivative compounds as potential antimicrobial agents and determined their interactions with the crystal structure of the protein receptor mutant TEM-12 from *E. coli* (pdb:1ESU) using molecular docking via UCSF Chimera software 1.17.3. The results suggest that **27** has potential as a natural antibiotic agent.

## 1. Introduction

**Figure 1:**
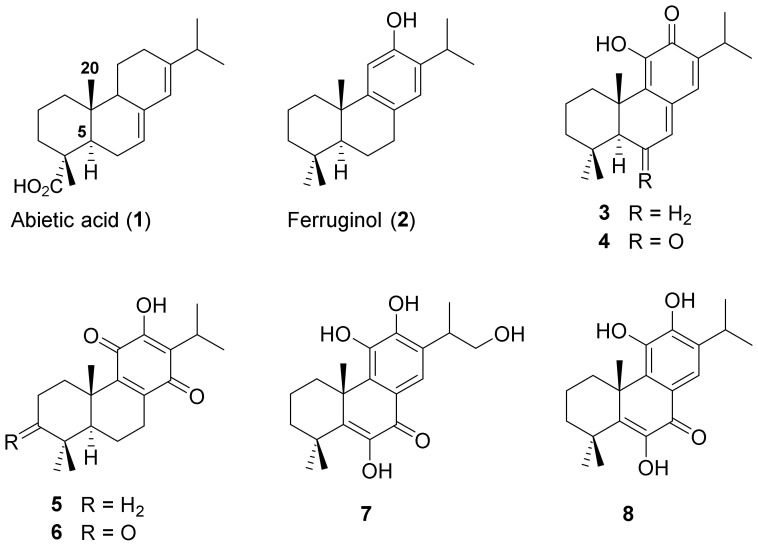
Examples of abietane diterpene compounds **1**–**8**.

Natural products are substances produced by living organisms and typically manifest as secondary metabolites. Within this group, terpenoids represent a significant and diverse class of compounds. Terpenoids can be classified on the basis of the number of carbon atoms they contain. Diterpenoids, for example, consist of twenty carbons. Among them, abietane diterpenoids have garnered attention because of their natural occurrence in a wide variety of plants. These compounds are characterized by a tricyclic ring system [1], such as abietic acid (**1**), ferruginol (**2**), 6-deoxytaxodione (**3**), and taxodione (**4**) (Figure 1). They have been isolated from *Cupressus sempervirens* L., a species of cypress native to the eastern Mediterranean region, and have demonstrated great and potent antibacterial activities (IC_50_: 0.80 and 0.85 μg/mL) against methicillin-resistant *Staphylococcus aureus* [2]. Ferruginol (**2**) also inhibits non-small-cell lung cancer growth by inducing caspase-associated apoptosis [3]. In addition, other compounds such as royleanone (**5**), **6**–**7**, and 6-hydroxysalvinolone (**8**) (Figure 1) have been extracted from *Clerodendrum eriophyllum*, a plant native to Kenya [2]. These abietane diterpenoids exhibit various potent biological activities. Thus, 6-hydroxysalvinolone (**8**) showed strong antibacterial activity against *Staphylococcus aureus* and methicillin-resistant *S. aureus*, with IC_50_/MIC values ranging between 0.96–1.56/2.5 μg/mL. Furthermore, synthetic derivatives of aromatic abietane diterpenoids also possess antiplasmodial, antileishmanial, and antifungal properties [4,5].

**Figure 2:**
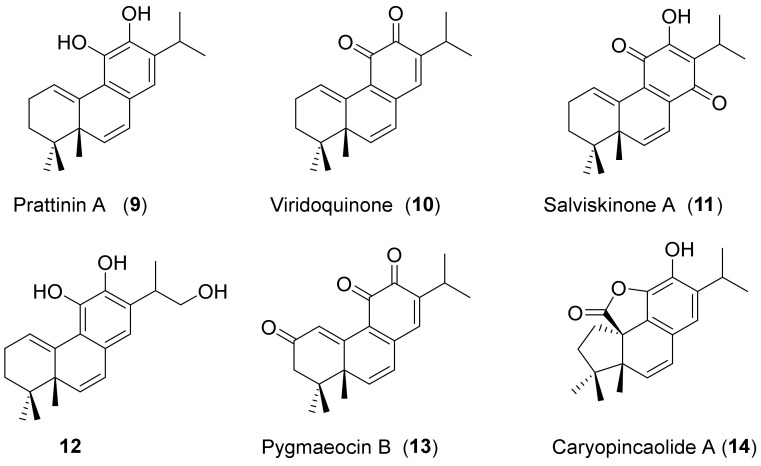
Rearranged abietane diterpenes **9**–**14**.

Rearranged-abietane-type diterpenes represent a smaller subgroup of secondary metabolites, and they are particularly noteworthy because of their intriguing structural and biological properties. When examining the arrangement of their core structures, for example, prattinin A (**9**) (Figure 2) stands out as an abietane diterpenoid with an unusual rearranged skeleton with a methyl group at the C-5 position, which was recently isolated through methanolic extraction from the roots of *Salvia prattii* (Lamiaceae) [6]. Moreover, it is noteworthy to highlight prattinin A-related compounds, including viridoquinone (**10**), salviskinone A (**11**), its hydroxyl derivative **12**, pygmaeocin B (**13**), and caryopincaolide A (**14**) (Figure 2) [7]. Pygmaeocin B exhibited significant activity against HT29 antitumor cells, showing the highest cytotoxicity (IC_50_ = 6.69 ± 1.2 μg/mL) and the strongest anti-inflammatory potential (IC_50NO_ = 33.0 ± 0.8 ng/mL) [8].

The first synthesis of rearranged abietane diterpenes related to pygmaeocin B (**13**) and viridoquinone (**10**) from ferruginol (**2**) was recently described by our group [8]. Microorganisms such as pathogenic bacteria have been responsible for several human diseases. Moreover, the indiscriminate use of antibiotics has contributed to the development of antibiotic resistance among pathogenic bacteria [9]. Therefore, considering the above data and our ongoing efforts in the pursuit of bioactive compounds, we described the first synthesis of prattinin A (**9**) from abietic acid (**1**). Furthermore, we evaluated the antimicrobial activity of the synthesized intermediate compounds and prattinin A derivatives against *Escherichia coli*, *Pseudomonas aeruginosa*, and *Staphylococcus aureus.*

## 2. Results

### 2.1. Chemistry

**Scheme 1:**
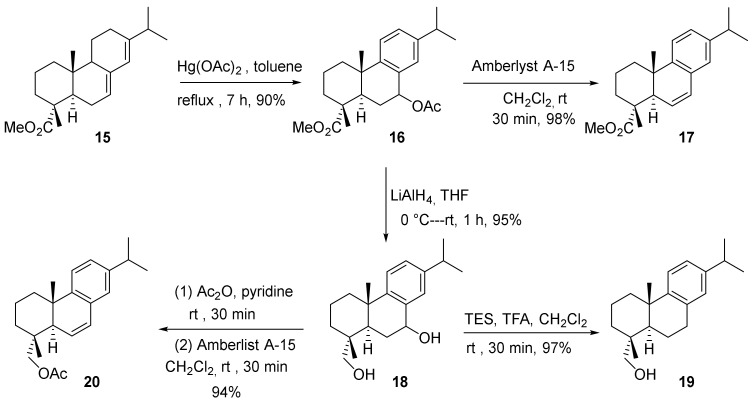
Synthesis of compounds **17**, **19**, and **20** from abietic ester **15**.

Building upon our previous work, we established a biosynthetic pathway for the synthesis of viridoquinone (**10**) and pygmaeocin B (**13**) and explored their anti-cancer and anti-inflammatory activities [8]. In this study, we synthesized prattinin A (**9**) and abietane derivatives **17**, **19**, **20**, **24**–**35**, and **37** from abietic acid (**1**). The latter was transformed into its ester derivative **15**. Therefore, the synthesis of abietane derivatives **17**, **19**, and **20** (Scheme 1) was performed.

The first step involved the oxidation and aromatization of abietic ester **15** to yield compound **16** [10] using mercury(II) acetate (Hg(OAc)_2_) under refluxing conditions in toluene. Subsequent treatment with Amberlyst A-15 resulted in the formation of derivative **17** [7]. In the presence of LiAlH_4_ in THF, compound **16** was reduced to form intermediate **18** [11]. The latter was efficiently converted into hydroxy-dehydroabietic **19** [12] in a high yield using triethylsilane (TES) in the presence of trifluoroacetic acid (TFA) in dichloromethane. On the other hand, compound **18** was transformed into **20** [7] by the action of Ac_2_O at 0 °C in pyridine and subsequent elimination of the acetate group by Amberlyst A-15.

Next, the synthesis of prattinin A derivatives (rearranged abietane) begins with the preparation of dehydroabietic ester **21** as the first step. For this transformation, various studies have used I_2_ as an aromatization agent [13,14], including the conversion of abietic ester **15** into dehydroabietic ester **21** using 1 equivalent of I_2_ in toluene at reflux (Table 1, Entry 1). In this study, we examined this reaction in the presence of I_2_, which now serves as a catalyst, while changing the solvent to dimethyl sulfoxide (DMSO).

In this study, the use of 5% of I_2_ at room temperature resulted in a single aromatic product **21** with a 92% yield after 7 days (Entry 2). Increasing the temperature to 70 °C under the same conditions as before, the reaction resulted in complete conversion after 9 h, with the formation of two products: aromatic compounds **21** (80%) and **17** (20%) (Entry 3). On the other hand, raising the temperature to 160 °C (Entry 4) revealed intriguing changes; after 9 h at this temperature, we observed the disappearance of compound **17** and the emergence of two new products, quinone **22** (10%) and ketone **23** (15%), in addition to dehydroabietic ester **21** (75%).

**Scheme 2:**
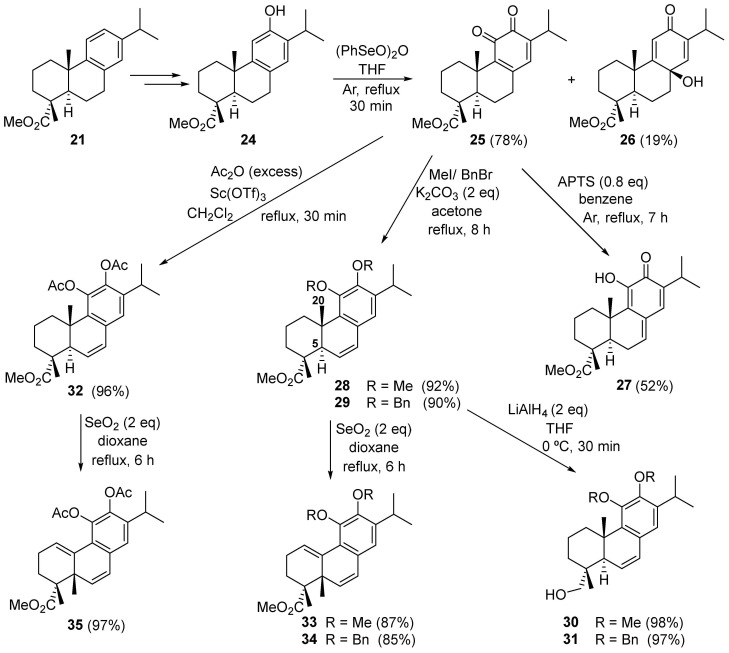
Synthesis of o-quinone ester **25**, 6-deoxytaxodione derivative **27**, and derivatives of prattinin A (**9**) **30**–**31**, **33**–**35**.

The synthesis of the 6-deoxytaxodione derivative **27** and other rearranged compounds from the dehydroabietic ester **21** was performed as described in Scheme 2. In the first step, **21** was transformed into phenol **24** following previously described conditions [12]. Subsequently, the oxidation of compound **24** with (PhSeO)_2_O (see Reference [7]) resulted in complete conversion, yielding orthoquinone **25** and hydroxy dienone **26**, respectively, in 78% and 19% yields (Scheme 2). The action of a Brønsted acid such as *p*-toluenesulfonic acid (PTSA) [15] on orthoquinone-ester **25** in anhydrous benzene led, after 7 h, to the formation of 6-deoxytaxodione derivative **27** [16], with a yield of 52%.

In addition, treatment of orthoquinone ester **25** under a basic environment using K_2_CO_3_, along with either methyl iodide or benzyl bromide in acetone at reflux, resulted in the synthesis of di-o-methoxy-abietane **28** (or di-*o*-benzyl-abietane **29**) with high efficiency, achieving yields of up to 92% (and 90%) after 8 h.

Considering the biological significance of abietane-hydroxylated products, certain abietane-type hydroxylated diterpenoids and their derivatives exhibit a wide range of cytotoxic, anti-proliferative, antibacterial, anti-parasitic, and anti-inflammatory activities [17].

In this context, with the goal of preparing additional products that could be significant in terms of antibacterial activity, we reduced compounds **28** and **29** using LiAlH_4_ in anhydrous THF at 0 °C. After 30 min, the reaction yielded the corresponding hydroxyl-abietanes **30** (98%) and **31** (97%). Conversely, orthoquinone **25** was transformed into diacetate **32** in high yield using Ac_2_O in the presence of catalytic Sc(OTf)_3_ in dichloromethane under reflux. Subsequently, treatment of compounds **28**, **29**, and **32** with two equivalents of SeO_2_ in dioxane under reflux led to the formation of the corresponding rearranged products **33**, **34**, and **35** after 6 h, in good yields (87%, 85%, and 97%, respectively).

**Scheme 3:**
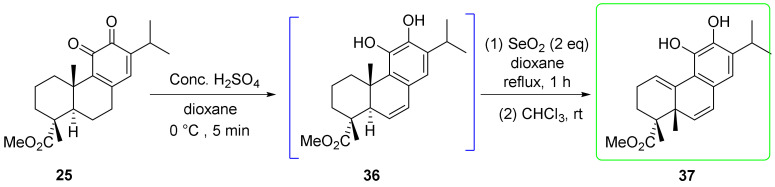
Synthesis of the prattinin A-derivative **37**.

In addition, prattinin A-derivative **37** was synthesized from ester **25** (Scheme 3). Treating the latter with concentrated H_2_SO_4_ in dioxane at 0 °C under an argon atmosphere resulted in an almost quantitative yield of intermediate **36** (an unstable catechol). Subsequently, the catechol was immediately subjected to the next reaction using SeO_2_ under reflux, followed by stirring in chloroform, leading to the formation of prattinin A-derivative **37** (70%).

**Scheme 4:**
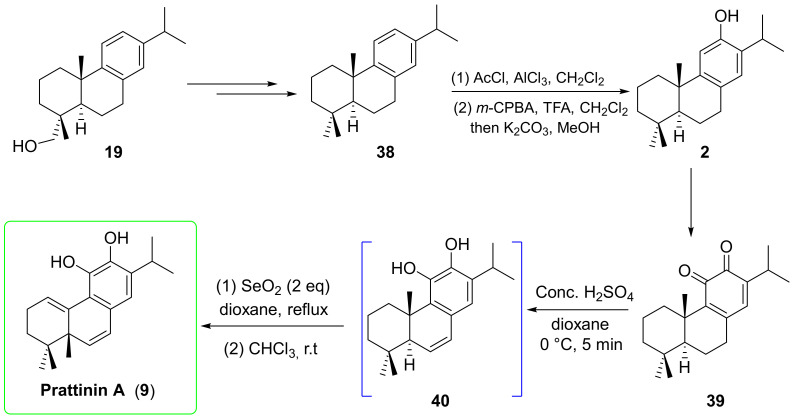
Synthesis of prattinin A (**9**) from compound **19**.

Finally, prattinin A (**9**) was synthesized from compound **19** (Scheme 4) using the same synthetic protocol as that for its derivative **37** (Scheme 3).

The first step involved the transformation of alcohol **19** into 8,11,13-abietatriene **38** through previously described reaction [18]. Subsequently, ferruginol (**2**) was formed in two steps from **38** using the Friedel–Crafts reaction, followed by a Baeyer–Villiger transformation, and concluded with deprotection in a basic medium [19], as described in Scheme 4. The oxidation of ferruginol (**2**) was then examined using the (PhSeO)_2_O promoted reaction, as described in our previous work [8], resulting in quinone **39**. In the presence of concentrated sulfuric acid in dioxane at 0° C, orthoquinone **39** was immediately reduced to an unstable intermediate **40**, which rearranged and transformed into the target product prattinin A (**9**) in the presence of SeO_2_ in refluxing dioxane. The reaction was then followed by stirring in chloroform at room temperature for 2 h, yielding **9** in 75% yield. The NMR spectroscopic data of prattinin A (**9**) were identical to those of natural products (see SI, Tables S2 and S3).

### 2.2. Antibacterial Activity

#### Disc Diffusion Test

The in vitro antibacterial activity of abietane diterpene derivatives (such as abietane ester derivative **17**, alcohols **19**, **30**–**31**, phenol **24**, quinone **25**, hydroxy dienone **26**, taxodione derivative **27**, as well as **28**–**29** and **32**), along with rearranged compounds (prattinin A (**9**) and its derivatives **33**–**35** and **37**), was initially evaluated qualitatively by measuring the inhibition diameter using the agar disc diffusion method against the human pathogenic bacteria *E. coli* (ATCC 25922), *P. aeruginosa* (CIP A22), and *S. aureus* (ATCC 25923). The obtained results are presented in Table 2. The compounds exhibited inhibitory activity ranging from moderate to excellent compared with the standard antibiotic ciprofloxacin (Table 2). Among the active products, compounds **25** and **27** displayed the best antibacterial activity. Overall, compound **27** exhibited excellent inhibition activity against all tested pathogenic bacteria, whereas compound **25** was more active against Gram-negative *E. coli* and *P. aeruginosa* than against Gram-positive *S. aureus*. Compounds **26** and **30** also showed significant inhibition activity against *S. aureus* and *P. aeruginosa*. In contrast, compounds **17**, **19**, **28**, **32**, **33**, and **35** showed moderate antibacterial activity (Table 2).

The antibacterial activity of these compounds was quantitatively estimated by measuring the minimal inhibitory concentration (MIC) using the microdilution broth assay. The results are presented in Table 3. The studied compound showed variable MIC against the tested bacteria, with compounds **25**, **27**, and **30** exhibiting the most potent activity with the lowest MIC values. Indeed, compound **27** exhibited the best inhibition activity against Gram-negative *E. coli* and *P. aeruginosa* with an MIC value of 11.7 µg/mL, which is close to that of the standard antibiotic (10 µg/mL), and against Gram-positive *S. aureus* with an MIC of 23.4 µg/mL. Compounds **27** and **30** showed excellent activity against Gram-negative *E. coli* and *P. aeruginosa* with MICs ranging from 23.4 to 46.9 µg/mL. Compound **30** also exhibited an important inhibition against *S. aureus* with an MIC of 46.9 µg/m compared with the standard (Table 3). These results are consistent with previous findings in the literature on the antibacterial activity of diterpenoids against Gram-negative and Gram-positive bacteria and highlight the potential application of these compounds to control pathogenic and multidrug-resistant bacteria [5,20].

### 2.3. ADMET: In Silico Prediction of Physicochemical and ADME Properties

#### 2.3.1. Physicochemical Parameters

The drug pharmacokinetic profile is a key step in the selection of a lead candidate in a drug discovery program because it is critical to biological potency and selectivity by optimizing the administration route. The most active synthesized compounds **25**, **27**, and **30** have been subjected to computational studies to predict their oral bioavailability using Lipinski’s rules of five (LRo5) and Veber’s rules for drug-likeness [21,22]

For this purpose, Molsoft L.L.C. (San Diego, CA, USA) and Swiss ADME free access servers were used. The generated results are shown in Table 4. Lipinski established some rules for drug bioavailability: (i) a molecular weight ≤500 g/mol; (ii) an octanol–water partition coefficient ≤5; (iii) less than five hydrogen bond donor atoms; and (iv) no more than ten hydrogen bond acceptor atoms [21]. According to the data, the size inferred from the molar mass of the mentioned compounds is less than 500, indicating that the three molecules have almost the same molar mass (344.20–344.24 g/mol), which indicates that they can be easily metabolized in comparison with larger molecules. Log P suggests that lipophilicity plays a significant role in drug discovery and compound design because it can determine pharmacokinetic processes such as drug absorption, distribution, and excretion [23]. Log *p*-values for products **25** and **27** are in the recommended range: 4.19 and 4.23, respectively, except for compound **30**, which has a higher log *p*-value of 5.36. Peculiarly, all compounds possess 3 to 4 H-bond acceptors and 0 to 1 H-bond donors. Two of the three selected compounds were found to obey Lipinski’s rules, and found to have drug-like character, except compound **30** with one violation, which could explain its lower exhibited antibacterial potential.

The polar surface area (polarity) and the count of rotatable bonds (flexibility) have been found to better determine oral active compounds. Veber´s conditions state that for a compound to be administered orally, it should comply with the following chemical features: (i) the polar surface area must be no greater than 140 Å^2^, and (ii) the number of rotatable bonds must be less than 10. The PSA values of the three selected compounds varied from 32.63 Å^2^ to 47.74 Å^2^, and the sum of rotatable bonds was between 3 and 4, which indicated promising oral availability for all compounds according to Veber’s parameters. Therefore, all selected compounds comply with Lipinski’s rules (except compound **30**, with 1 violation of LRo5: LogP = 5.36 ≥ 5) and Veber’s rules; hence, it can be concluded that they exhibited good drug-like properties. Therefore, they could be administered orally, and it is probable that they could be lead candidates.

#### 2.3.2. Absorption, Distribution, Metabolism, Excretion, and Toxicity (ADME) Properties

An efficient drug is a compound with high biological activity at a lower effective concentration and low toxicity. In addition, the pharmacokinetic properties of absorption, distribution, metabolism, and excretion (ADME) are crucial for developing new drug candidates. Thus, the evaluation of these properties is one of the most important preliminary procedures undertaken by the drug discovery industry to save time and reduce costs.

The ADME properties in silico calculations of the most active compounds **25**, **27**, and **30** were performed using online databases such as Swiss ADME [24] and pkCSM [25]. The results are shown in Table 5.

One of the most important parameters in drug discovery is drug absorption, which plays an essential role in drug bioavailability. Since the absorption of an orally administered drug occurs mainly through the small intestine, two intestinal absorption indicators were used: human intestinal absorption (HIA) and Caco-2 permeability. For a given compound, the HIA factor can predict the percentage that will be absorbed through the human intestine: the HIA percentage is considered high (70–100%), medium (20–70%), and low (0–20%). Caco-2 permeability can predict oral drug intake because Caco-2 from human colon carcinoma resembles intestinal epithelial cells. It should be mentioned that high Caco-2 permeability would translate into predicted values of Papp >8 × 10^−6^ cm/s [26]. According to these parameters, all compounds showed high human intestinal absorption ranging from 94.79% to 100% and high Caco-2 permeability (0.82–1.35). Consequently, they could be absorbed through the human intestine. The brain is protected from exogenous compounds by the blood–brain barrier (BBB). The ability of drugs to cross the blood–brain barrier is an advantageous parameter for improving the efficacy of drugs exerting their pharmacological activity on the central nervous system (CNS). This parameter can also be considered to help reduce the side effects and toxicities of therapeutic agents. In this way, the distribution profile of the drugs was predicted through the BBB crossing ability using SwissADME. Thus, all the selected compounds are considered to readily cross the BBB. CYP2C9 and CYP2C6, among others, are isoforms of cytochrome P450, which is an important enzyme mainly found in the liver and is responsible for drug metabolism, which oxidizes xenobiotics to facilitate their excretion. Therefore, it is important to assess a compound’s ability to inhibit cytochrome P450. In silico calculations indicate that all the tested compounds had no CYP interactions; thus, they are inhibitors of these cytochromes. The excretion profile was predicted through the total clearance of drugs, both hepatic (metabolism and biliary) and renal (excretion via the kidneys). It is important for determining the dosing rates and bioavailability of drugs [27]. All products showed a lower total clearance of 0.76–1.01 logml/min/kg. The AMES test is a widely employed method for assessing the mutagenic potential of compounds using bacteria. A positive AMES test indicates that the compound is mutagenic and may, therefore, act as a carcinogen. Hepatotoxicity is liver damage that can be caused by several agents, including drugs, toxins, and herbs. Drug-induced hepatic injury is the most common reason cited for withdrawal of an approved drug. The predicted toxicity of all our tested products (**25**, **27**, and **30**) through AMES toxicity parameters and hepatoxicity showed no mutagenic potential for these molecules. These results reinforce the theory that they could be good candidates for the design of oral drugs.

### 2.4. Molecular Docking

To study the stability of the three potent compounds **25**, **27**, and **30** as antimicrobial agents and determine the interactions between these compounds and the crystal structure of the protein receptor mutant TEM-12 from *E. coli* (pdb:1ESU), we applied molecular docking using USCF Chimera software 1.17.3.

**Figure 3:**
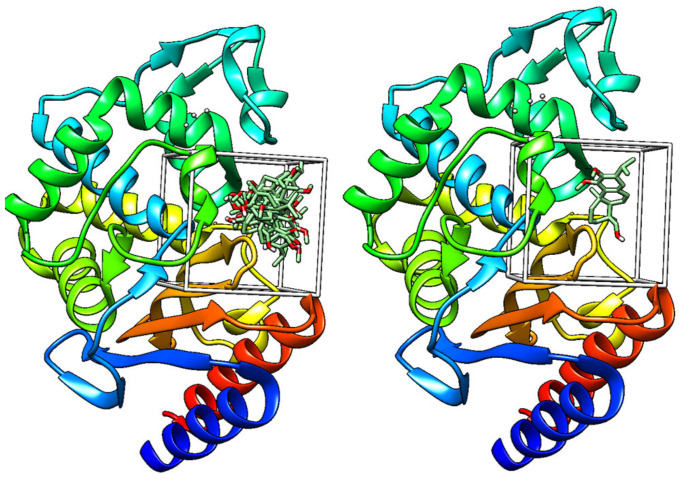
Possible positions of compound **30** and the stable ligand position inside the grid.

The re-docking of the co-crystal ligand showed a small RMSD value, which improves the reliability of the docking performed in further studies. Figure 3 shows the possible positions and the stable position of compound **30** as a ligand.

**Figure 4:**
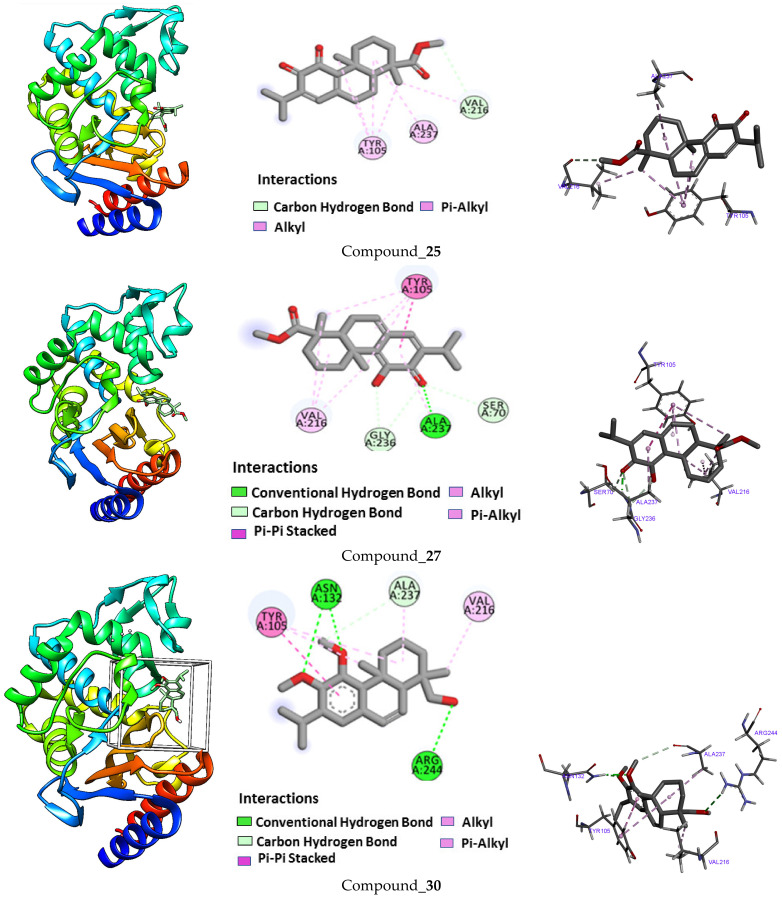
The interactions of compounds **25**, **27,** and **30** and protein receptor pdb:1ESU using Discovery Studio Visualizer and UCSF Chimera software 1.17.3.

The results of applied molecular docking of compound **25** showed three interactions with protein receptors: an alkyl interaction with the TYR A:105 residue, a π-alkyl interaction with ALA A:237, and a carbon–hydrogen interaction with VAL A:216. The energy affinity of these interactions shows an interesting score function of −6.9, which can explain the obtained activity. As described in Figure 4, compound **27** shows different π-π and π-alkyl interactions with both VAL A:216 and TYR A:105 residues, carbon–hydrogen interactions with both GLY A:236 and SER A:70 and a hydrogen bond with ALA A:237 residue. The ligand binding results showed a high affinity with a score function of −7.2. The stable position of compound **30** in the receptor pocket shows two hydrogen bonds with both ASN A:132 and ARG A:244 residues and π-alkyl interactions. The score function obtained is −7.4, which explains the stability of this ligand.

### 2.5. DFT Computational Studies

#### 2.5.1. Global Reactivity Descriptors

The reactivity descriptors, based on the analysis of the electronic chemical potential (µ), chemical hardness (η), softness (σ), electrophilicity (ω), and electronegativity (χ) index, provide useful insight into the chemical reactivity and stability of the molecules. Therefore, chemical hardness (η) and softness (σ) are essential properties for measuring the reactivity and molecular stability of molecules. Chemical hardness measures resistance to changes in electron distribution or charge transfer and corresponds to the gap between the HOMO and LUMO. The larger the energy gap, the harder the molecule becomes and the more stable/less reactive [28]. As shown in Table S1 (see Supplementary Materials), compound **27** is the most stable because it has a hardness value comparable to the other molecules. Therefore, compound **25** will react more easily with other systems, given that softness is the reciprocal of hardness.

The electrophilicity index (ω) measures the capacity of a species to accept electrons (the electrophilic tendency of the molecule). It is a measure of the stabilization in energy after a system has accepted an additional amount of electronic charge from the environment [29]. The global electrophilicity index measures the stabilization of energy when the system acquires an additional electronic charge from the environment. Table 6 shows that compound **25** has a higher electrophilicity index value (3.653 a.u.) than the other compounds. High electrophilicity index values increase the electron-accepting abilities of the molecules.

Electronegativity is the measure of an atom, molecule, or solid substance’s ability to attract electrons to itself. The first connection of the electronegativity (χ) concept with quantum mechanics within density functional theory (DFT) was made by Parr et al. [30]. Furthermore, it was proposed that (μ) is the negative of electronegativity (χ), which helped to establish a direct connection with chemical reactivity [31]. The higher the electronegativity of the species, the greater its electron-accepting power or electrophilicity. Table 6 shows the order of electronegativity as **27** > **25** > **26**> … compounds. The electronegativity values indicate that compounds **27** and **25** have the highest tendency to attract electrons; hence, this compound would be energetically favored for nucleophilic attack.

On the other hand, we conclude that molecules **28** to **34** have almost the same values of chemical reactivity descriptors.

#### 2.5.2. Molecular Electrostatic Potential (MESP)

The molecular electrostatic potential (MESP) at a point in the space around a compound provides information about the net electrostatic effect produced at that point by the total charge distribution of the molecule [32]. Moreover, MESP helps to understand the relative polarity of a molecule and serves to explain the reactivity, residual interaction, polarizability, and structure–activity relationship of biomolecules and drugs [33].

The MESP maps shown in Table 6 indicate that there are negative potential zones characterized by red color around the oxygen atoms. A relatively larger region around the oxygen atoms of the C=O functions, reflected by the yellowish blobs, represents the most negative potential region (dark red) and is permissible for electrophilic interaction. The hydrogen atom carries the maximum force of the positive charge (dark blue), whereas the neutral potential is localized on the aromatic ring surfaces and is represented by green [34].

## 3. Materials and Methods

### 3.1. Chemistry

Experimental details of the synthesis of all new substrates are described in the Supplementary Materials.

### 3.2. Biological Activity

#### 3.2.1. Antibacterial Test

The antibacterial activity of compounds **9**, **17**, **19**–**20**, **24**–**35**, and **37** was evaluated in vitro against three human pathogenic bacteria, including Gram-negative *Escherichia coli* (ATCC 25922) and *Pseudomonas aeruginosa* (CIP A22), and Gram-positive *Staphylococcus aureus* (ATCC 25923), using the agar disc diffusion method and the microdilution broth assay. The tested bacteria were kindly provided by the Pasteur Institute of Casablanca, Morocco

#### 3.2.2. Agar Disc Diffusion Method

The agar disc diffusion method was performed following the procedure described by Smaili et al. [35]. Briefly, sterile cellulose discs of 6 mm diameter impregnated with 10 µL of the tested compounds at a concentration of 1.5 mg/mL dissolved in DMSO were placed on Mueller–Hinton agar previously amended with 3 × 10^8^ CFU mL^−1^ of bacteria. A disc containing DMSO was used as the negative control, and a disc containing the standard antibiotic ciprofloxacin was used as the positive control. After 24 h of incubation at 37 °C, the inhibition diameter around the discs was measured, compared with the standard antibiotic, and interpreted as follows: ≤8 mm: no activity; 8 < D ≤ 12 mm: moderate activity; 12 < D ≤ 14 mm: significant activity; and D > 14 mm: excellent activity. All experiments were performed in triplicate, and the results are expressed as mean value ± standard deviation.

#### 3.2.3. Microdilution Broth Assay

The microdilution broth assay was performed to estimate the minimum inhibitory concentration (MIC) of the studied compounds against pathogenic bacteria using 96-well microdilution plates as described by Anthony et al. [36]. For each compound, a series of solutions were prepared in Mueller–Hinton broth using the two-fold dilution method at 2.92 to 1500 µg/mL. Subsequently, 100 µL from each compound’s solution and 10 μL of bacterial suspension (adjusted at 0.5 McFarland) were added to the 96-well plates and incubated at 37 °C for 24 h. The compound solutions were replaced by Mueller–Hinton broth and DMSO as negative controls, and ciprofloxacin as a positive control. After incubation, the optical density was measured at 600 nm using a UV/Vis spectrophotometer, and the MIC was determined as the lowest concentration of each compound that could inhibit the growth of the pathogenic bacteria. All experiments were conducted in triplicate, and the results were expressed in mean value ± standard deviation.

### 3.3. Molecular Docking

#### 3.3.1. In Silico Studies

Molecular docking is a computational tool for estimating the minimum energy generated between a specific target and ligand. The effective ligand against a protein is selected on the basis of the minimum docking score between protein–ligand interactions. In this study, we performed molecular docking studies using the UCSF Chimera software 1.17.3 with its AutoDock Vina [37] tool to explain the biological activity of the three most active compounds. These compounds were used as ligands against the target protein mutant TEM-12 from *E. coli* (pdb:1ESU) retrieved from the protein data bank [38].

#### 3.3.2. Ligand Preparation

The three-dimensional structures of the studied compounds were drawn using ChemSketch software (Version 14.01) [39] in mol format. Structure minimization of all ligands was performed using UCSF Chimera before conducting molecular docking analysis.

#### 3.3.3. Protein Preparation

The target receptor, a mutant TEM-12 from *E. coli*, was prepared by retrieving the three-dimensional crystal structure from the RCSB protein bank (pdb:1ESU). Energy minimization and geometry optimization were performed using Dock Prep, a built-in tool for preparing the structure before docking in UCSF Chimera to add hydrogen atoms and charges. The protein was later saved in the PDBQT format.

#### 3.3.4. Molecular Docking with Autodock Vina

Following ligand and receptor preparation, molecular docking analysis was performed using UCSF Chimera’s built-in AutoDock Vina tool to determine binding affinities and different ligand–receptor interactions. After the minimization process, the grid box resolution was set at 11.5466, 10.2182, and 09.2616 along the x, y, and z points, respectively, at a grid resolution of 1 Å, and the grid dimensions were set at 20, 20, and 20 Å. The resulting interactions were visualized using Discovery Studio software (https://discover.3ds.com/discovery-studio-visualizer-download, accessed on 25 January 2024) [40].

### 3.4. DFT Computational Studies

#### Computational Details

DFT calculations were applied in aqueous media to correlate the results of experimental studies with the understanding of the efficiency of organic molecules and to observe quantum chemical parameters [41]. The quantum computation technique applied in this study is the B-3LYP (Becke-3-parameters-Lee-Yang-Parr) level at 6–31 G(d′,p′) base set, using Gaussian 09 and GaussView 5.0.8 software [42,43]. Furthermore, theoretical research on corrosion inhibitors necessitates the development of a set of descriptors: global reactivity and local selectivity.

The important parameter descriptors of global reactivity, such as the energy gap (ΔE), electronic affinity (EA), ionization potential (EI), electronegativity (χ), chemical hardness (η), global softness (σ), electrophilicity (ω), and electronegativity (χ) index, were calculated using the Equations (1)–(7), to explain the chemical reactivity of the newly synthesized molecular [44,45]:

IE = −E HOMO(1)

EA = −E LUMO(2)

η = 1/2 (IE − EA) = 1/2 (−E HOMO + E LUMO)(3)

σ = 1/η(4)

χ = 1/2(IE + EA) = 1/2 (−E HOMO − E LUMO)(5)

μ = −1/2(IE + EA) =−χ(6)

ω = μ^2^/2η (7)

## 4. Conclusions

In conclusion, this study enhances our understanding of abietane-derived compounds and their rearranged derivatives, highlighting their potential applications in various biological activities. The successful synthesis of prattinin A (**9**) and its new derivatives from abietic acid (**1**) yielded impressive yields of up to 98%. Our biological investigations demonstrated significant antibacterial potential in these compounds, particularly orthoquinone **25**, its isomer 6-deoxytaxodione derivative **27,** and alcohol **30**. These compounds exhibited variable minimum inhibitory concentrations (MICs) against the tested bacteria, with compounds **25**, **27**, and **30** displaying the most potent activity, boasting the lowest MIC values. In particular, compound **27** exhibited remarkable inhibition against Gram-negative *E. coli* and *P. aeruginosa*, with an MIC of 11.7 µg/mL, which is close to that of the standard antibiotic (10 µg/mL). It also showed notable activity against Gram-positive *S. aureus*, with an MIC of 23.4 µg/mL. Compounds **27** and **30** displayed excellent activity against Gram-negative *E. coli* and *P. aeruginosa*, with MIC values ranging from 23.4 to 46.9 µg/mL. Compound **30** also exhibited substantial inhibition against *S. aureus*, with an MIC of 46.9 µg/mL. In addition, the absorption, distribution, metabolism, excretion, and toxicity (ADME) properties of these potentially active compounds were studied. Molecular docking analyses revealed promising interactions between these compounds. Compound **25** demonstrated significant interactions with specific residues, which were closely associated with its observed activity, achieving an affinity score of −6.9. Compound **27** displayed diverse interactions, including π-π and π-alkyl interactions, along with a hydrogen bond, resulting in a high-affinity score of −7.2. Compound **30** exhibited stability with hydrogen bonds and π-alkyl interactions, leading to an affinity score of −7.4. These docking results align with our experimental findings. Furthermore, DFT computational studies indicated that compounds **25** and **27** have the highest electronegativity values, suggesting their strong affinity for attracting electrons and favoring nucleophilic attacks. These results agree with previous research on the antibacterial properties of diterpenoids against both Gram-negative and Gram-positive bacteria, underscoring the potential utility of these compounds in combating pathogenic and multidrug-resistant bacteria. This study offers promising prospects for the development of new antibacterial agents.

## Figures and Tables

**Table 1:** Treatment of abietic ester **15** with I_2_ at different temperatures.

* 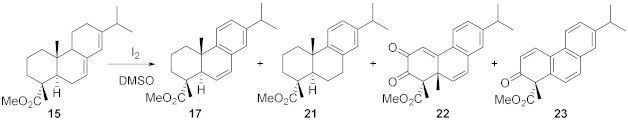 *		
Entry	Conditions	Products (Yield%)
1	1 equiv. I_2_, toluene, reflux, 6 h [13]	**21** (84)
2	5% I_2_, DMSO, rt, 7 days	**21** (92)
3	5% I_2_, DMSO, 70 °C, 9 h	**21** (80), **17** (20)
4	5% I_2_, DMSO, 160 °C, 9 h	**21** (75), **22** (10), **23** (15)

**Table 2:** Inhibition zones (mm) of compounds **9**, **17**, **19**–**20**, **24**–**35**, and **37** and controls (ciprofloxacin) on pathogenic bacteria.

Compound	Inhibition Zones (mm)		
	*E. coli* (ATCC 25922)	*S. aureus* (ATCC 25923)	*P. aeruginosa* (CIP A22)
**9**	9 ± 0.1	10 ± 0.1	10 ± 0.1
**17**	NE	NE	NE
**19**	11 ± 0.2	12 ± 0.1	13 ± 0.5
**20**	NE	NE	NE
**24**	11 ± 0.1	11 ± 0.1	12 ± 0.4
**25**	15 ± 0.2	14 ± 0.4	15 ± 0.3
**26**	NE	14 ± 0.5	11 ± 0.1
**27**	17 ± 0.5	18 ± 0.1	16 ± 0.8
**28**	9 ± 0.2	10 ± 0.1	12
**29**	NE	10 ± 0.1	11 ± 0.1
**30**	11 ± 0.1	13 ± 0.6	14 ± 0.7
**31**	NE	11 ± 0.1	11 ± 0.1
**32**	9 ± 0.2	9 ± 0.2	11 ± 0.2
**33**	11 ± 0.1	13 ± 0.2	12
**34**	NE	NE	NE
**35**	10 ± 0.1	10 ± 0.2	12 ± 0.1
**37**	9 ± 0.12	11 ± 0.05	11 ± 0.5
Ciprofloxacin	20 ± 0.1	24 ± 0.1	25 ± 0.1
DMSO	/	/	/

/: Inactive; NE: no effect; ±: standard deviation.

**Table 3:** Minimum inhibitory concentration (MIC) (µg/mL) of compounds **9**, **17**, **19**–**20**, **24**–**35**, and **37** and controls (ciprofloxacin) on pathogenic bacteria.

Compound	MIC (µg/mL)		
	*E. coli* (ATCC 25922)	*S. aureus* (ATCC 25923)	*P. aeruginosa* (CIP A22)
**9**	750 ± 0.1	450 ± 0.05	450 ± 0.01
**17**	NE	NE	NE
**19**	375 ± 0.2	187.5 ± 0.2	375
**20**	750 ± 0.2	375 ± 0.3	500 ± 0.1
**24**	187.5 ± 0.1	187.5 ± 0.1	375 ± 0.1
**25**	46.9 ± 0.1	93.7 ± 0.1	46.9 ± 0.2
**26**	750 ± 0.1	500 ± 0.1	750 ± 0.23
**27**	11.7 ± 0.1	23.4 ± 0.1	11.7 ± 0.1
**28**	375 ± 0.1	500	375 ± 0.1
**29**	750	500 ± 0.2	500 ± 0.1
**30**	46.9 ± 0.1	46.9 ± 0.3	23.4 ± 0.2
**31**	750	500 ± 0.2	500 ± 0.1
**32**	750 ± 0.1	500 ± 0.1	187.5 ± 0.1
**33**	187.5 ± 0.2	187.5 ± 0.1	375 ± 0.1
**34**	NE	NE	NE
**35**	375 ± 0.1	500	375 ± 0.1
**37**	750 ± 0.11	500 ± 0.06	500 ± 0.01
Ciprofloxacin	10 ± 0.2	10 ± 0.1	10 ± 0.1
DMSO	/	/	/

/: Inactive; NE: no effect; ±: standard deviation.

**Table 4:** Pharmacokinetic analysis of compounds **25, 27**, and **30**.

Product	MW	LogP	HBD	HBA	nVs	nRB	MolPSA
Lipinski *	≤500	≤5	≤5	≤10	≤1	_	_
Veber **	_	_	_	_	_	≤10	≤140 Å^2^
25	344.20	4.19	0	4	0	3	47.74 Å^2^
27	344.20	4.23	1	4	0	3	49.46 Å^2^
30	344.24	5.36	1	3	1	4	32.63 Å^2^

* Lipinski reference values. ** Veber reference values; MW, molecular weight; LogP, lipophilicity (O/W); HBD, number of hydrogen bond donors; HBA, number of hydrogen bond acceptors; nVs, number of Lipinski’s rule violations; nRB, number of rotatable bonds; and MolPSA, molecular polar surface area (PSA) (Å^2^).

**Table 5:** ADME properties of compounds **25**, **27**, and **30**.

Compound	Absorption		Distribution	Metabolism		Excretion	Toxicity	
	HIA (%)	Caco-2 (Log Papp)	BBB (Log BB)	CYP2C9 Inhibitor	CYP2D6 Inhibitor	Total Clearance (Log CLtot)	AMES	Hepatotoxicity
**25**	100.0	1.35	Yes	No	No	1.01	No	No
**27**	95.91	0.82	Yes	No	No	0.93	No	No
**30**	94.79	1.30	Yes	No	No	0.76	No	No

HIA, human intestinal absorption (%); Caco-2 (Log Papp in 10^−6^ cm/s), permeability through Caco-2 (human colorectal carcinoma) cells in vitro; BBB, blood–brain barrier penetration; CYP2C9, Cytochrome P450 2C9; CYP2C6, Cytochrome P4502C6; total clearance (Log CLtot in mL/min/kg); AMES, *Salmonella typhimurium* reverse mutation assay; and hepatotoxicity, liver-induced damage caused by exposure to a drug.

**Table 6:** MESP maps of compounds **24**–**31**, **33**, and **34** showing areas of negative electrostatic potential.

**24**	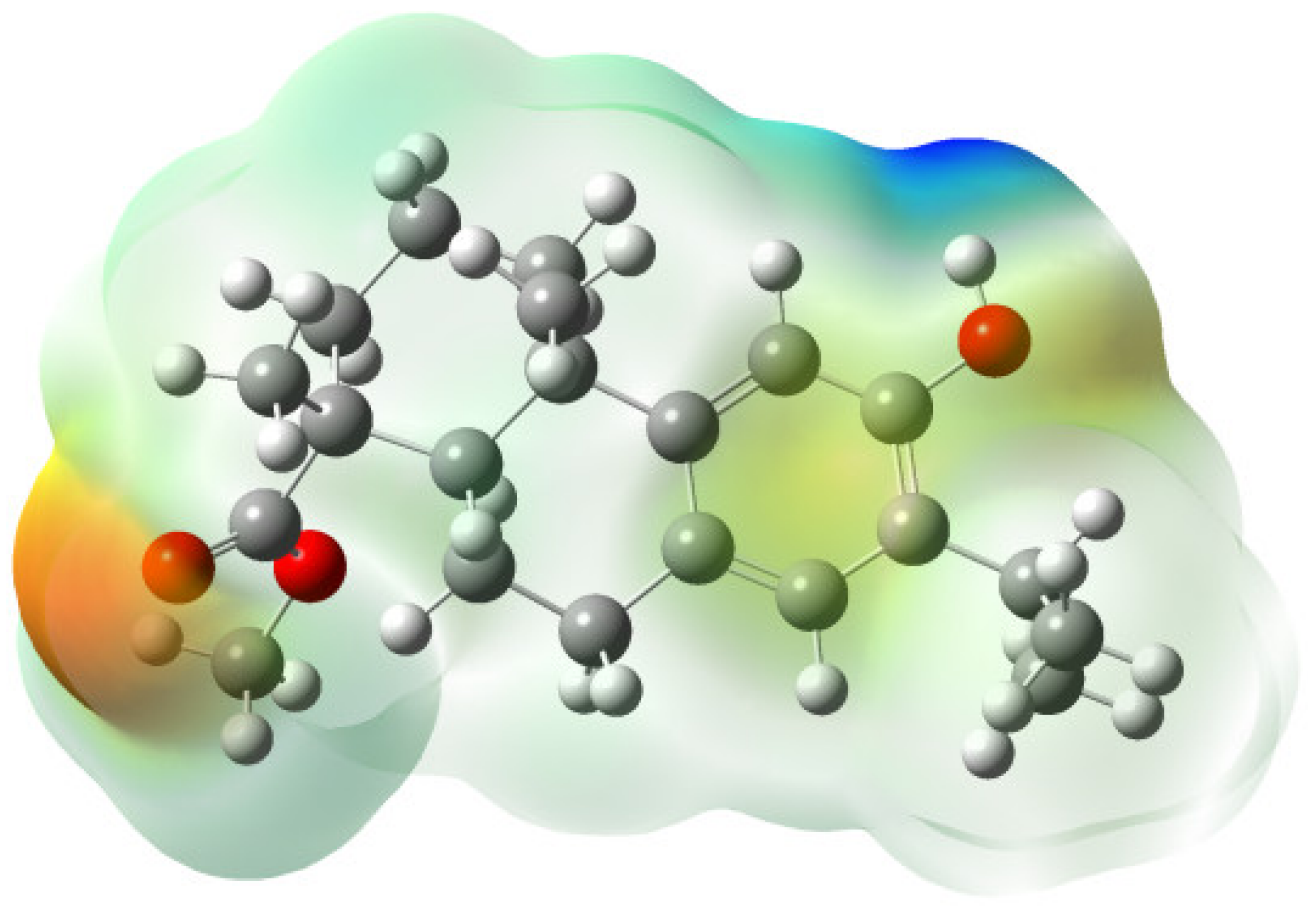	**25**	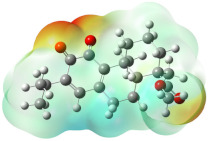
**26**	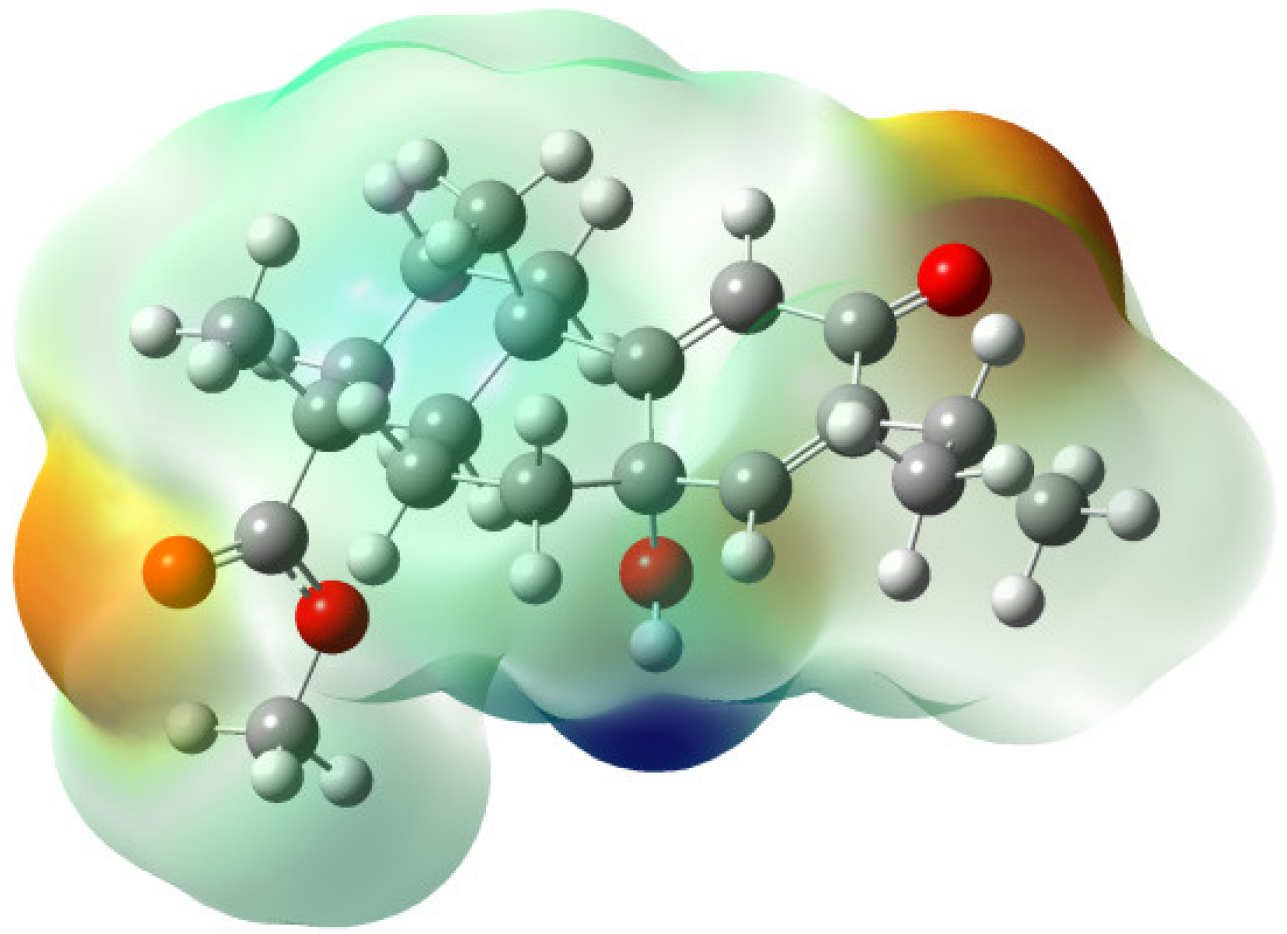	**27**	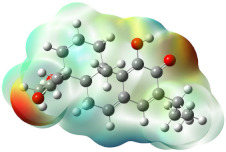
**28**	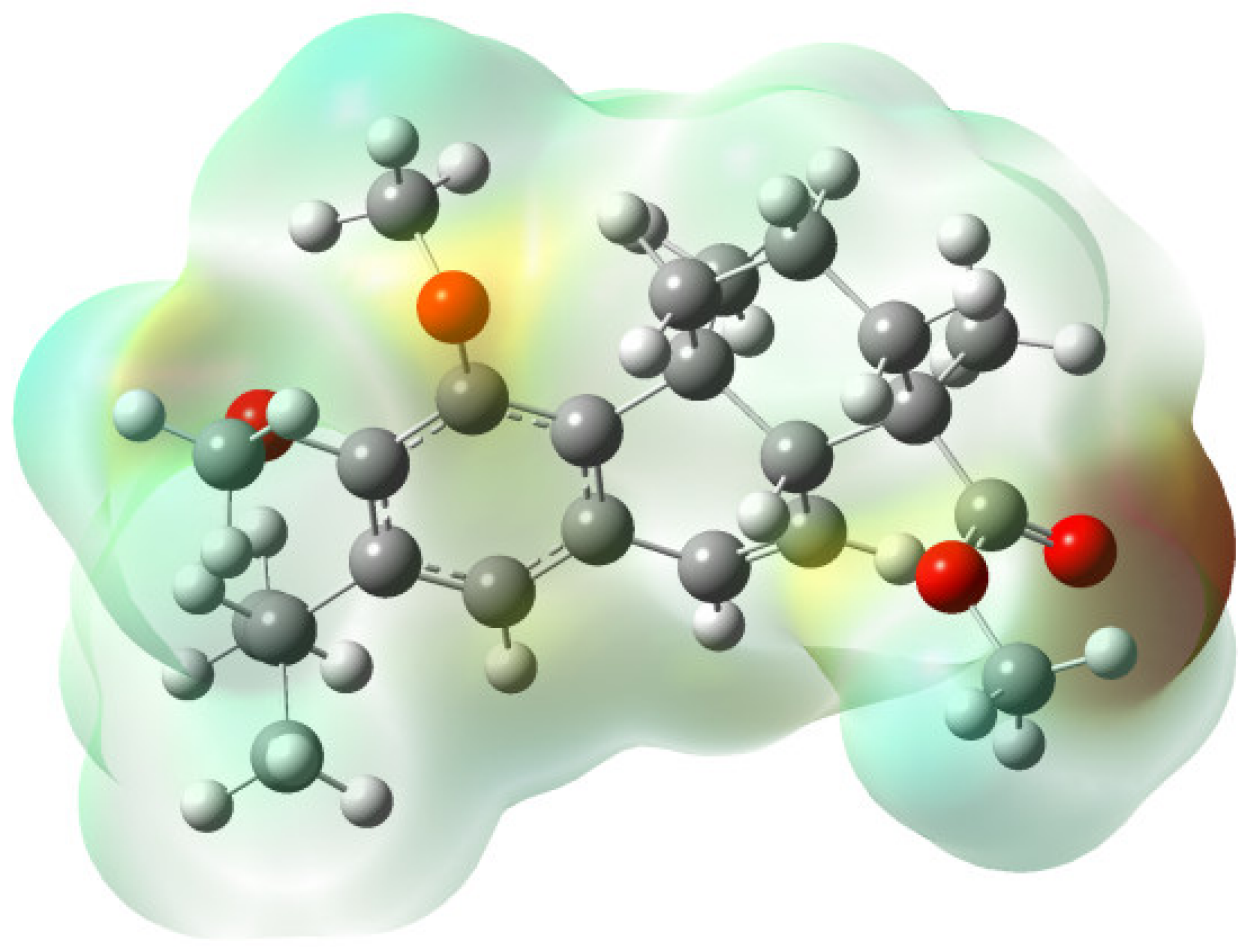	**29**	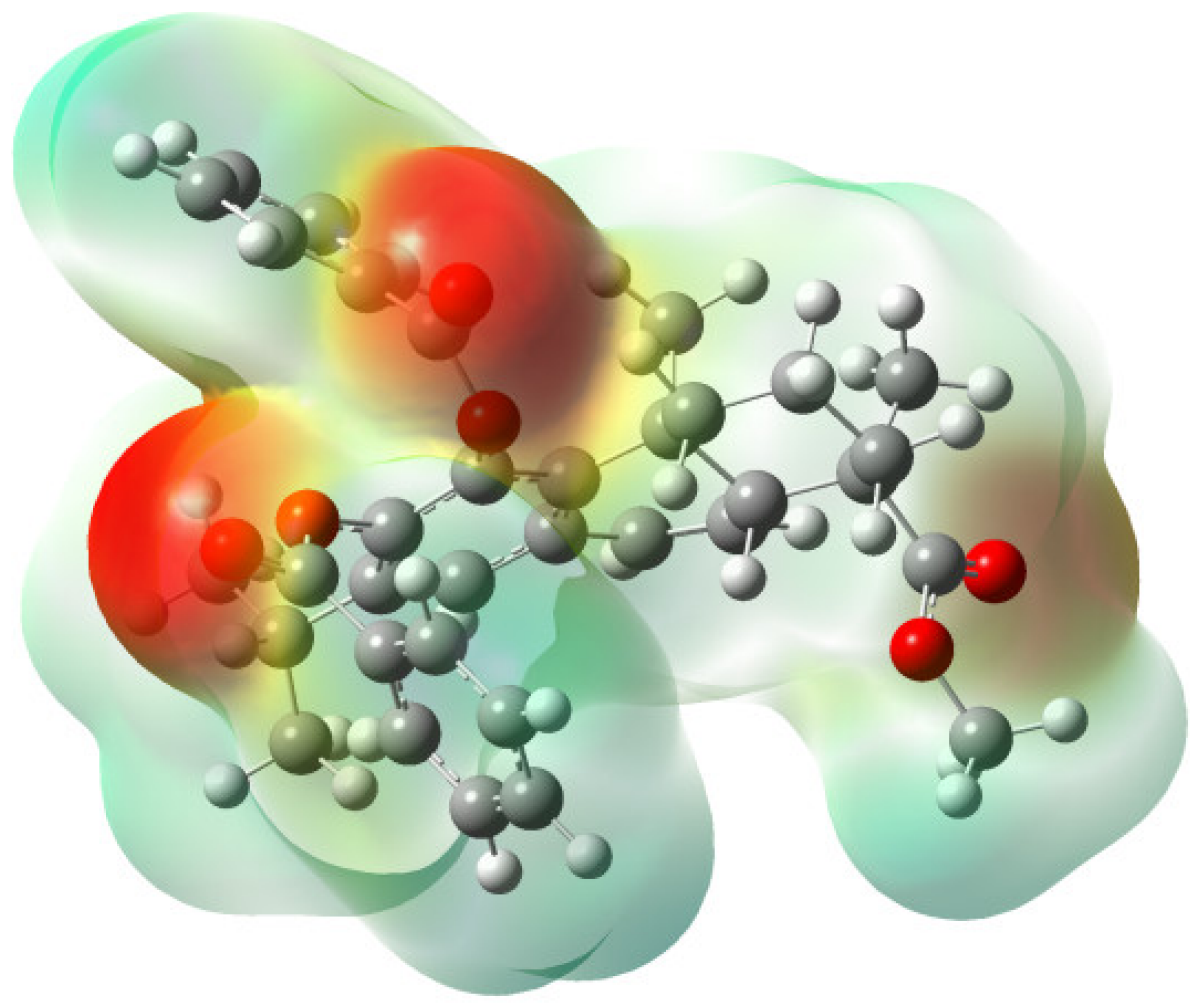
**30**	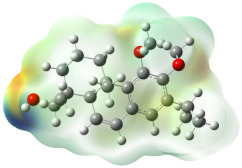	**31**	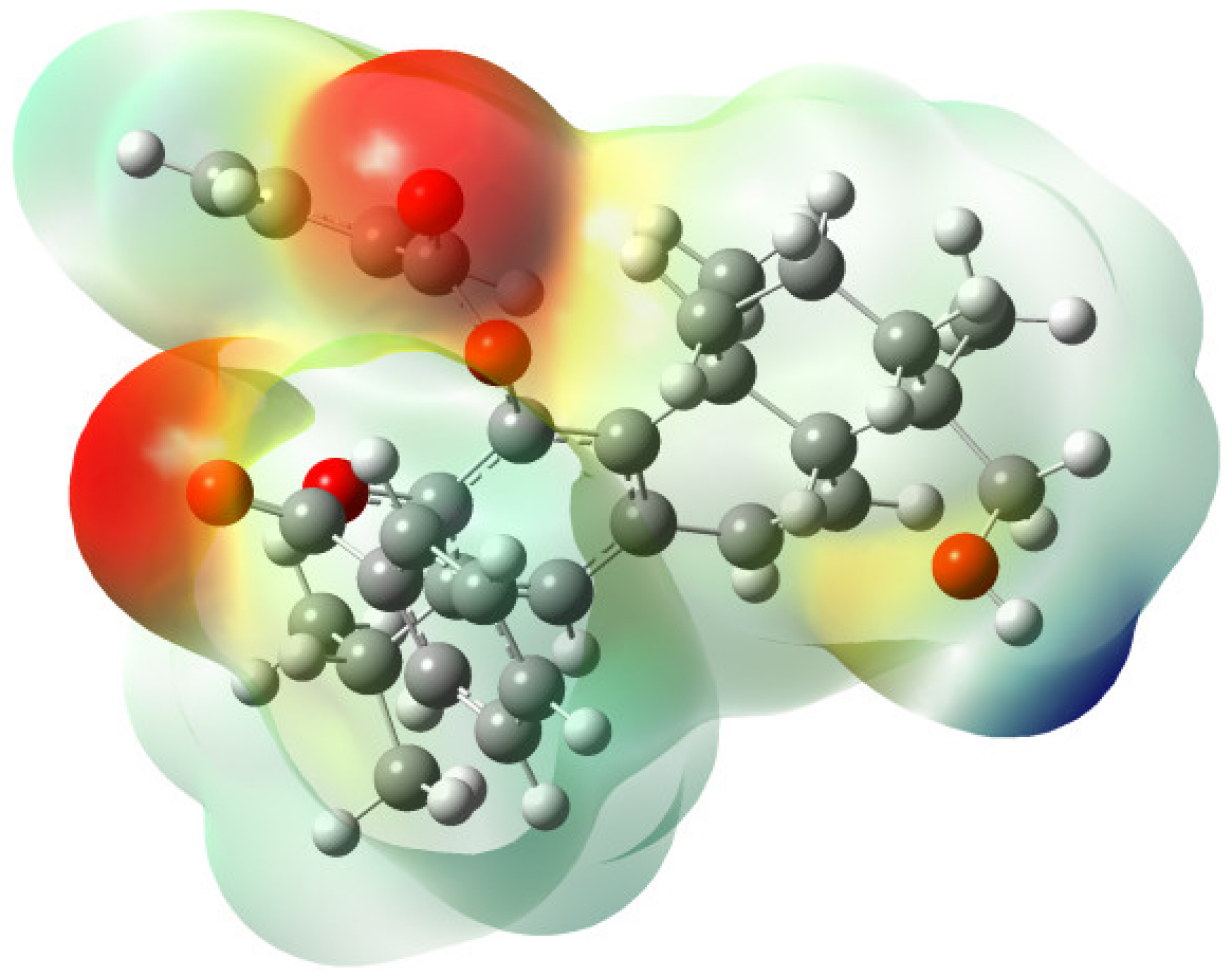
**33**	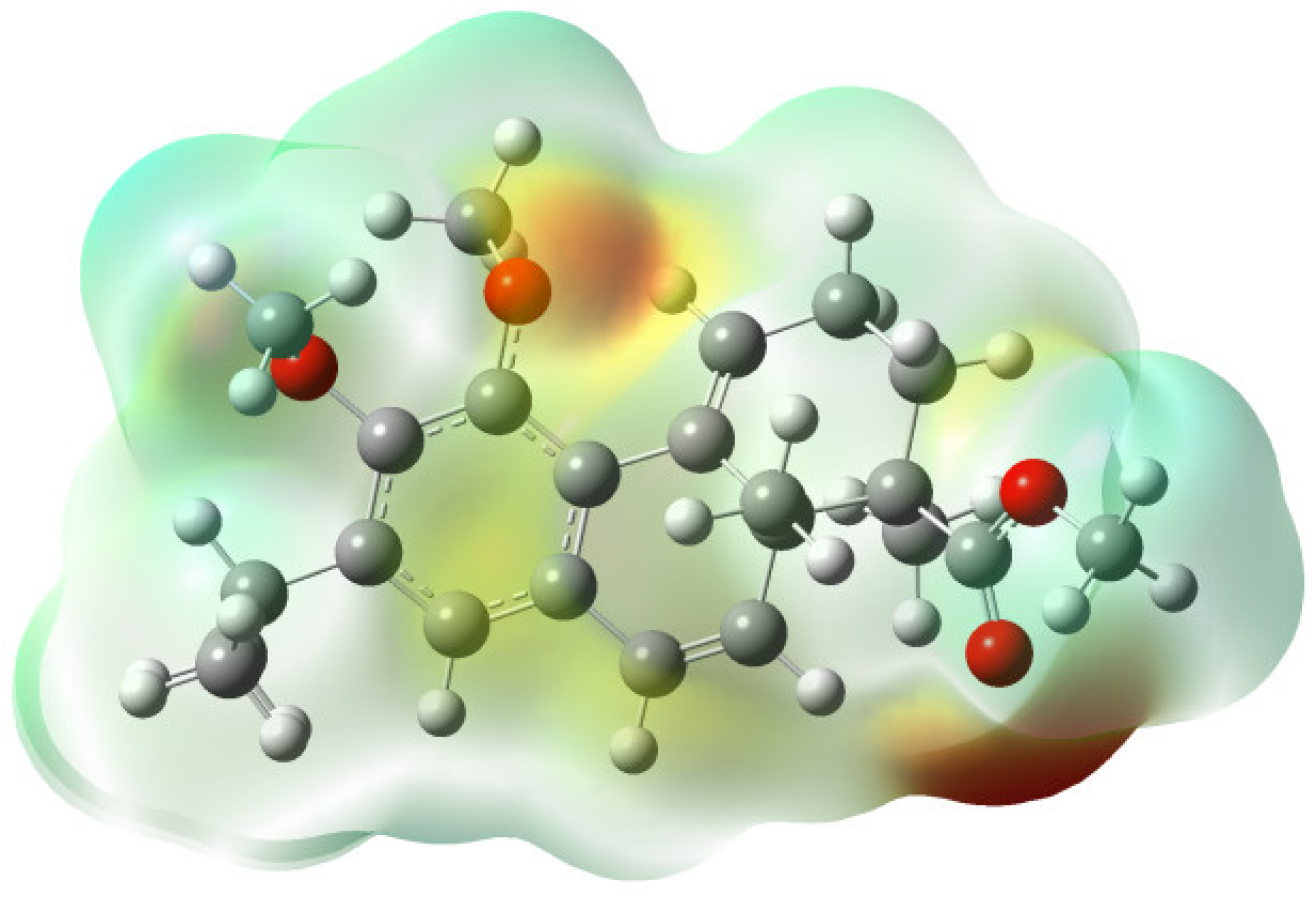	**34**	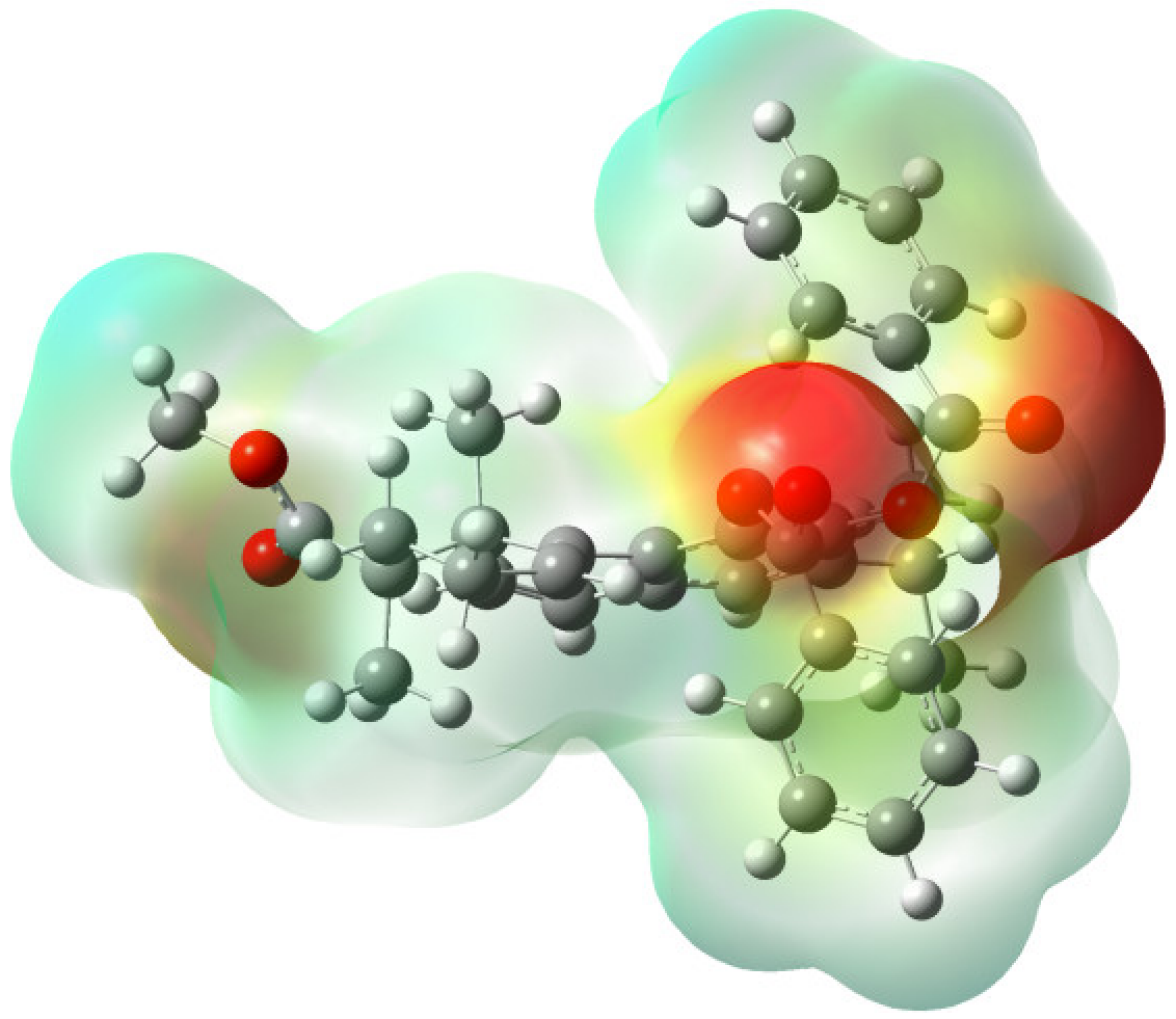

## Data Availability

Data are contained within the article or Supplementary Materials.

## Supplementary Materials

The following supporting information can be downloaded at: https://www.mdpi.com/article/10.3390/molecules29030650/s1, Experimental details of the synthesis of all new substrates. Copy of ^1^H NMR and ^13^C NMR spectra of all new compounds.
