# Supplementary material for: Synthesis and Evaluation of Antimicrobial Activity of the Rearranged Abietane Prattinin A and Its Synthetic Derivatives
Source: Molecules. 2024 Jan 30;29(3):650. doi: 10.3390/molecules29030650 (PMC10856147; doi:10.3390/molecules29030650)
Supplement: Supplementary file 1 [file molecules-29-00650-s001.zip › molecules-2829114-supplementary.pdf]

Supporting Information

Synthesis and Evaluation of Antimicrobial Activity of the Rearranged Abietane Prattinin A and its Synthetic Derivatives

Mustapha Ait El Had [1,2], Manal Zefzoufi [3], Houda Zentar [1], Lahoucine Bahsis [4], Mouhi Eddine Hachim [4], Adib Ghaleb [4], Choukri Khelifa-Mahdjoubi [1], Hafida Bouamama [3], Ramón Alvarez-Manzaneda [5], José Justicia [1*], Rachid Chahboun [1 *]

1 Departamento de Química Organica, Facultad de Ciencias, Universidad de Granada, 18071 Granada, Spain; mustapha.aitelhad20@gmail.com (M.A.E.H.); choukri_khelifa@hotmail.com (C.K.-M.)
2 Département des Sciences Fondamentales, Faculté de Médecine et de Pharmacie, et de Médecine Dentaire de Fès, Université Sidi Mohamed Ben Abdellah de Fès, Fes 30000, Morocco
3 Recherche en Développement Durable et Santé, Faculté des Sciences et Techniques, Cadi Ayyad University, Marrakech 40000, Morocco; zefzoufi.manal1994@gmail.co (M.Z.); bouamamahafida@gmail.com (H.B.)
4 Laboratory of Analytical and Molecular Chemistry, Polydisciplinary Faculty, Cadi Ayyad University, BP 4162, Safi 46000, Morocco; bahsis.lahoucine@gmail.com (L.B.); mouhieddinehachim@gmail.com (M.E.H.); adib.ghaleb@gmail.com (A.G.)
5 Área de Química Orgánica, Departamento de Química y Física, Universidad de Almería, 04120 Almería, Spain; ralvarez@ual.es
* Correspondence: jjusti@ugr.es (J.J.); rachid@ugr.es (R.C.); Fax: +34-958-248437 (J.J.&R.C.)

- Energies of the HOMO, LUMO, energy gap, and global chemical reactivity indices of the synthesized compounds S2
- Experimental Procedures S3-S21
- ^{1}H, ^{13}C NMR and mass spectra of compounds S22-S90

Table S1. Energies of the HOMO, LUMO, energy gap, and global chemical reactivity indices of the synthesized compounds.

**Compounds**	**E_{HOMO} (eV)**	**E_{LUMO} (eV)**	**ΔE**	**IP**	**EA**	**μ**	**η**	**σ**	**ω**	**χ**
**24**	-5.58	0.064	5.644	5.58	-0.064	-2.758	2.822	0.354	0.673	2.758
**25**	-6.345	-3.217	3.128	6.345	3.217	-4.781	1.564	0.639	3.653	4.781
**26**	-6.553	-1.935	4.618	6.553	1.935	-4.244	2.309	0.433	1.950	4.244
**27**	-8.81	-1.195	7.615	8.81	1.195	-5.002	3.807	0.262	1.643	5.002
**28**	-5.603	-0.784	4.819	5.603	0.784	-3.193	2.409	0.415	1.058	3.193
**29**	-5.953	-1.346	4.607	5.953	1.346	-3.649	2.303	0.434	1.445	3.649
**30**	-5.522	-0.654	4.868	5.522	0.654	-3.088	2.434	0.410	0.979	3.088
**31**	-5.784	-1.22	4.564	5.784	1.22	-3.502	2.282	0.438	1.343	3.502
**33**	-5.339	-0.842	4.497	5.339	0.842	-3.090	2.248	0.444	1.061	3.090
**34**	-5.719	-1.412	4.307	5.719	1.412	-3.565	2.153	0.464	1.475	3.565

General Procedures

Except where otherwise indicated, The reactions were carried out under argon atmosphere using dry solvents. Solvents were dried as follows: THF and dioxane over Na–benzophenone, toluene over Na, dichloromethane (DCM) over CaH2. Thin-layer chromatography (TLC) was performed using F254 precoated plates (0.25 mm) and visualized by UV fluorescence quenching and phosphomolybdic acid solution staining. Flash chromatography was performed on silica gel (230-400 mesh). Chromatography separations were carried out by conventional column on silica gel 60 (230-400 Mesh), using hexanes-AcOEt (AcOEt-hexane) mixtures of increasing polarity. 1H and 13C NMR spectra were recorded at 500 and 400 MHz, and at 125 and 100 MHz, respectively. $CDCl_3$ was treated with K_2CO_3. Chemical shifts (δ H) are quoted in parts per million (ppm) referenced to the appropriate residual solvent peak and tetramethylsilane. Data for 1H NMR spectra are reported as follows: chemical shift (δ ppm) (multiplicity, coupling constant (Hz), integration), with the abbreviations s, br s, d, br d, t, q, sext and m denoting singlet, broad singlet, doublet, broad doublet, triplet, quartet, sextet and multiplet, respectively. J = coupling constant in Hertz (Hz). Data for 13C NMR spectra are reported in terms of chemical shift relative to Me4Si (δ 0.0) and the signals were assigned utilizing DEPT experiments and on the basis of heteronuclear correlations. Infrared spectra (IR) were recorded as thin films or as solids on a FTIR spectrophotometer with samples between sodium chloride plates or as potassium bromide pellets and are reported in frequency of absorption (cm^{-1}). HRMS were recorded on a spectrometer, utilizing a Q-TOF analyzer, and ESI^+ ionization.

Experimental Procedures

Synthesis of compounds16, and 17 from 15

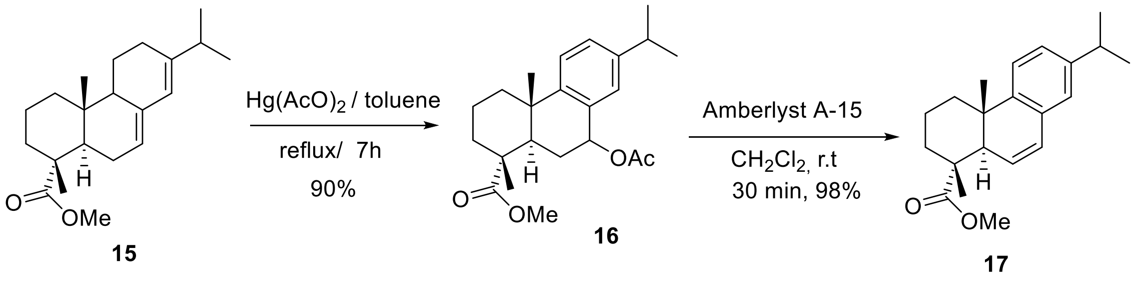

To a solution of compound **15** (1.3 g, 4.11 mmol) in toluene (10 mL) was added $Hg(OAc)_2$ (2.65g, 8.28 mmol) and the mixture was stirred at reflux for 7 hours, at which time TLC showed no starting material. Then, the mixture was filtered, and the solvent was removed

under vacuum. The resulting crude product was purified by flash column chromatography, using 5% EtOAc/hexanes to afford product **16**[1] (1.37 g, 90%) as a white solid.

To a solution of **16** (0.5 g, 1.34 mmol) in DCM (8 mL), was added Amberlyst A-15 (0.3 g) and the mixture was stirred at room temperature for 30 min, at which time TLC showed no starting material. Then, the mixture was filtered and the solvent was removed under vacuum to give a crude product which was purified by column chromatography using 2% EtOAc/hexanes to afford product **17**[2] (410 mg, 98%) as a colourless syrup.

Synthesis of product 20 from 16

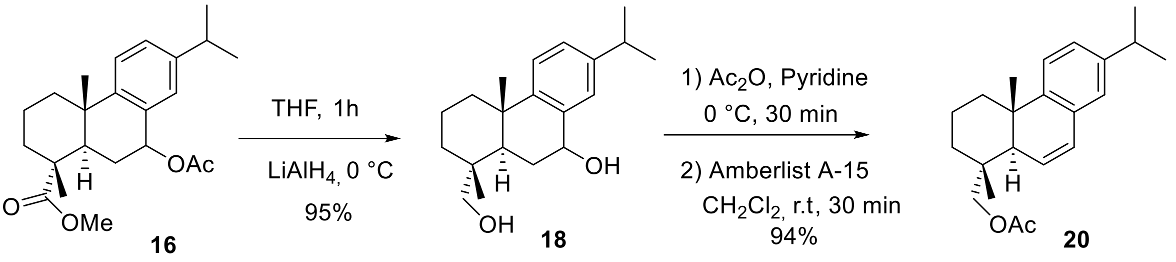

To a solution of compound **16** (270 mg, 0.726 mmol) in anhydrous THF (5 ml) was added at 0°C $LiAlH_4$ (40 mg, 1.05 mmol) and the mixture was stirred at room temperature for one hour. After verifying by TLC that the reaction is complete, the reaction crude is poured slowly onto ice, and AcOEt (15 mL) was added. the phases were shaken and separated. The organic phase was washed with water (2 x 5 ml), brine (5 mL), and dried over anhydrous sodium sulfate. After filtration and evaporation of the solvent, the diol product **18**[3] was obtained quantitatively (219 mg).

The latter diol **18** without purification was dissolved in pyridine (3 ml) and Ac_2O (2 ml) was added. The resulting mixture was stirred at room temperature for 30 minutes. After this time, TLC indicated the complete consumption of the starting material. Water was added and stirred for 6 minutes. Meanwhile, 20 ml of diethyl ether was added and the phases were shaken and separated. The organic phase was washed with 2 N HCl (5 x 10 ml), water (2 x 15 ml), saturated $NaHCO_3$ solution (3 x 10 ml), and brine (2 x 10 ml). The mixture was then dried over anhydrous Na_2SO_4 and filtered. The solvent was evaporated under vacuum to obtain the crude product which was dissolved in DCM (5 mL) and treated by Amberlyst A-15 (200 mg) for 30 min, to give, after filtration, compound **20**[2] (222 mg, 94%) as a colourless syrup.

Treatment of 18 with the system TES/TFA

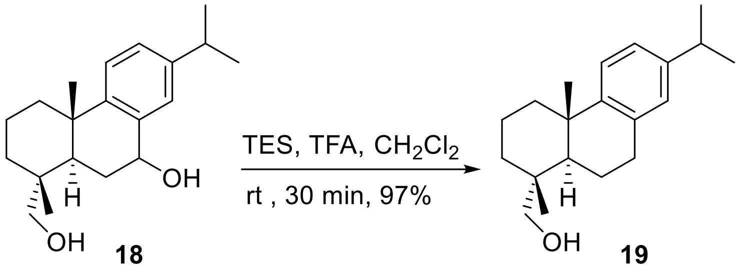

To a solution of **18** (158 mg, 0.52 mmol) in DCM (6 mL) were added Triethylsilane (TES) (0.5 mL) and TFA (0.2 mL) and the reaction mixture was stirred at room temperature for 30 min. Then, the solvente was removed under vacum and the crude product was directly purified by column chromatography using 10% EtOAc/hexanes to give compound **19**[4] (144 mg, 97%) as a colourles syrup.

Treatment of abietic methylester 15 by I_2/DMSO.(entry 4)

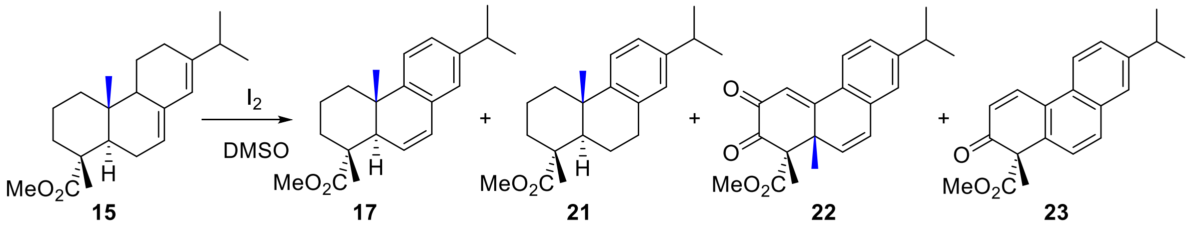

The action of 5% iodine (8 mg, 31.5 µmol) with abietic ester **15** (200 mg, 0.631 mmol), solubilized in the minimum amount of DMSO (4 ml) solvent at 160°C for 9 hours, leads to the complete transformation of the starting material. The reaction mixture is treated with water and dichloromethane. The organic phase is collected and dried over Na_2SO_4, and solvent concentration yields a crude product. The latter has been purified by column chromatography on silica gel, affording ester **21**[5] (128 mg, 75%) eluted with 10% EtOAc/hexane, dioxo-ester **22** (21.4 mg, 10%), and one oxo-ester **23** (29.2 mg, 15%) eluted with 20% EtOAc/hexane.

Methyl (1S,10aS)-7-isopropyl-1,10a-dimethyl-2,3-dioxo-1,2,3,10a-tetrahydrophenanthrene-1-carboxylate (22)

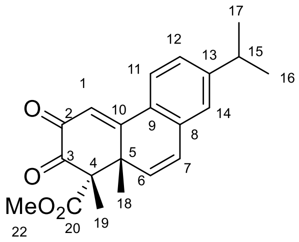

Viscous yellow. ^{1}H NMR (400 MHz, Chloroform-*d*) δ 7.80 (d, *J* = 8.3 Hz, 1H, H-11), 7.25 (dd, *J* = 8.3, 1.9 Hz, 1H, H-12), 7.12 (d, *J* = 1.9 Hz, 1H, H-14), 7.08 (s, 1H, H-1), 6.67 (d, *J* = 9.8 Hz, 1H, H-6), 6.33 (d, *J* = 9.8 Hz, 1H, H-7), 3.58 (s, 3H, H-19), 2.97 (hept, *J* = 6.9 Hz, 1H, H-15), 1.70 (s, 3H, H-18), 1.30 (d, *J* = 6.9 Hz, 3H, H-16), 1.24 (d, *J* = 6.9 Hz, 3H, H-17), 1.18 (s, 3H, H-22). ^{13}C NMR (101 MHz, Chloroform-*d*) 192.79 (C=O, C-3) , 181.21 (C=O, C-2), 170.65 (C=O, C-20), 166.00 (C, C-10), 154.75 (C, C-8), 133.83 (CH, C-12), 133.71 (C, C-13), 126.96 (CH, C-11), 126.03 (CH, C-14) , 125.82 (CH, C-1), 125.20 (C, C-9), 124.75 (CH, C-6), 121.94 (CH, C-7), 66.56 (C, C-4), 52.93 (CH_3, C-22), 47.53 (C, C-5), 34.14 (CH, C-15), 27.01 (CH_3, C-19), 23.51 (2 × CH_3, C-16, C-17), 13.18 (CH_3, C-18). HRMS (FAB-TOF) m/z: $[M + H]^+$ calcd for $C_{21}H_{22}O_4$; 339.1596 found, 339.1593.

<u>Summary table of assignment of NMR data of compound **22**</u>

Atom	δ (ppm)	HMBC	Atom	δ (ppm)	HMBC
1 C	121.94		13 C	154.90	15
1 H	7.00	3, 5, 9	14 C	126.03	
2 C	181.21		14 H	7.04	7, 12, 15
3 C	192.79	1, 18	15 C	34.14	12, 14
4 C	66.56	6, 18, 19	15 H	2.89	12, 13, 16
5 C	47.53	1, 6, 7, 18, 19	16 C	23.51	15
6 C	133.96	19	16 H3	1.21	
6 H	6.25	4, 5, 8, 10	17 C	23.51	
7 C	124.88	14, 18	17 H3	1.21	
7 H	6.59	5, 9	18 C	13.18	
8 C	133.71	6	18 H3	1.62	3, 4, 5, 7, 10, 20
9 C	125.30	1, 7, 12	19 C	27.01	
10 C	166.00	6, 18, 19	19 H3	1.10	4, 5, 6, 10
11 C	125.95		20 C	170.65	18, 22
11 H			22 C	52.93	
12 C	126.96	14, 15	22 H3	3.50	20

Methyl (S)-7-isopropyl-1-methyl-2-oxo-1,2-dihydrophenanthrene-1-carboxylate (23)

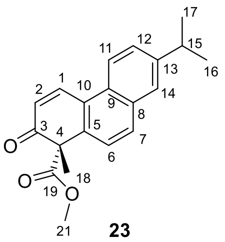

23

Viscous yellow.^{1}H NMR (500 MHz, Chloroform-*d*) δ 8.30 (d, *J* = 15.9 Hz, 1H, H-1), 7.97 (d, *J* = 8.85 Hz, 1H, H-11), 7.71 (d, *J* = 8.4 Hz, 1H, H-7), 7.63 (d, *J* = 1.9 Hz, 1H, H-14), 7.42 (dd, *J* = 8.8, 1.9 Hz, 1H, H-12), 7.33 (d, *J* = 8.4 Hz, 1H, H-6), 6.93 (d, *J* = 15.9 Hz, 1H, H-2), 3.83 (s, 3H, H-21), 3.07 (hept, *J* = 9.6 Hz, 1H, H-15), 2.08 (s, 3H, H-18), 1.35 (d, *J* = 6.9 Hz, 6H, H-16, H-17). ^{13}C NMR (126 MHz, Chloroform-*d*) δ 190.03 (C=O, C-3), 170.81 (C=O, C-19), 130.74 (C, C-10), 124.51 (CH, C-11), 130.27 (C, C-9), 145.74 (C, C-13), 132.44 (C, C-8), 133.9 (C, C-5), 124.64 (CH, C-14), 126.56 (CH, C-12), 128.29 (CH, C-2), 141.87 (CH, C-1), 128.7 (CH, C-7), 128.96 (CH, C-6), 61.4 (C, C-4), 170.8 (CH_3, C-19), 33.93 (CH, C-15), 19.65 (CH_3, C-18), 23.87 (CH_3, C-17), 23.88 (CH_3, C-16). IR (film): 2957, 2925, 2868, 1737, 1688, 1610, 1446, 1247, 1118, 1029, 823 cm^{-1}. HRMS (FAB-TOF) m/z: $[M + H]^+$ calcd for $C_{20}H_{21}O_3$; 309.1412 found, 309.1513.

Summary table of assignment of NMR data of compound **23**

Atom	δ (ppm)	HMBC	Atom	δ (ppm)	HMBC
1 C	141.87		12 C	126.56	
1 H	8.26	3, 5	12 H	7.38	10
2 C	128.29		13 C	145.74	11, 15
2 H	6.89	3, 10	14 C	124.64	
3 C	190.03	1, 2, 18	14 H	7.59	10
4 C	61.4	18	15 C	33.93	
5 C	133.9	1, 7	15 H	3.02	13
6 C	128.96		16 C	23.88	
6 H	7.29	8, 10	16 H3	1.31	17
7 C	128.7		17 C	23.87	16
7 H	7.67	5, 10, 11	17 H3	1.31	
8 C	132.44	6, 11	18 C	19.65	
9 C	130.27		18 H3	1.72	3, 4, 19
10 C	130.74	2, 6, 7, 11, 12, 14	19 C	170.8	18, 21
11 C	124.51	7	21 C	53.18	
11 H	7.93	8, 10, 13	21 H3	3.79	19

Treatment of phenol 24 with $(PhSeO)_2O$.

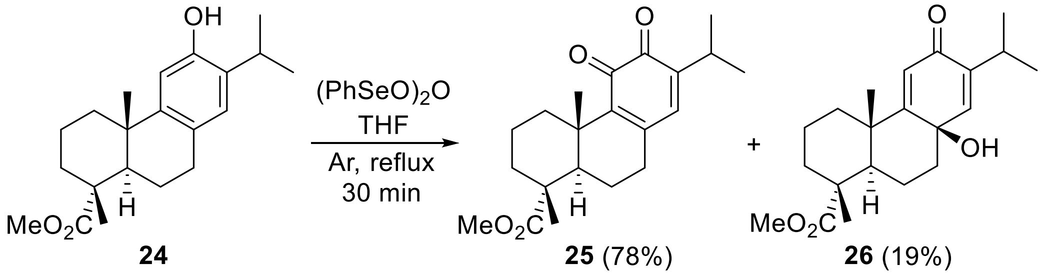

$(PhSeO)_2O$ (217 mg, 0.61 mmol) was aded to a solution of phenol **24** (200 mg, 0.61 mmol) in anhydrous THF (7 mL) under an argon atmosphere, and the reaction was kept stirring at reflux for 15 min. The solvent was evaporated under vacuum to give a crude product, which after column chromatography on silica gel, afforded quinone **25** (168 mg, 80%), eluted with 5% EtOAc/hexane, and hydroxy dienone **26** (42.3 mg, 20%), eluted with 10% EtOAc/hexane).

Methyl (1R,4aS,10aR)-7-isopropyl-1,4a-dimethyl-5,6-dioxo-1,2,3,4,4a,5,6,9,10,10a-decahydrophenanthrene-1-carboxylate (25)

Green solid , ^{1}H NMR (400 MHz, Chloroform-*d*) δ 6.40 (s, 1H), 3.70 (s, 3H), 2.91 (hept, *J* = 6.9 Hz, 1H), 2.78 (d, *J* = 12.6 Hz, 1H), 2.57 – 2.38 (m, 2H), 2.03 (dd, *J* = 12.4, 1.8 Hz, 1H), 1.76 – 1.55 (m, 4H), 1.45 – 1.31 (m, 1H), 1.27 (s, 3H), 1.25 (s, 3H), 1.12 (d, *J* = 2.5 Hz, 3H), 1.10 (d, *J* = 2.5 Hz, 3H). ^{13}C NMR (101 MHz, Chloroform-*d*) δ 180.93 (C=O), 179.91 (C=O), 178.77 (C=O), 147.93 (C), 146.95 (C), 144.10 (C), 137.57 (CH), 52.00 (CH), 47.57 (C), 45.47 (CH_3), 37.38 (C), 36.65 (CH_2), 35.21 (CH_2), 33.24 (CH_2), 26.89 (CH), 21.37 (2 × CH_3), 20.79 (CH_2), 20.18 (CH_3), 18.00 (CH_2), 16.51 (CH_3). IR (film): 1728, 1439, 1376, 1228, 1118 cm^{-1}. HRMS (FAB-TOF) m/z: $[M + H]^+$ calcd for $C_{21}H_{28}O_4$, 344.20, found: 345.2051.

Methyl (1R,4aS,8aR,10aR)-8a-hydroxy-7-isopropyl-1,4a-dimethyl-6-oxo-1,2,3,4,4a,6,8a,9,10,10a-decahydrophenanthrene-1-carboxylate (26)

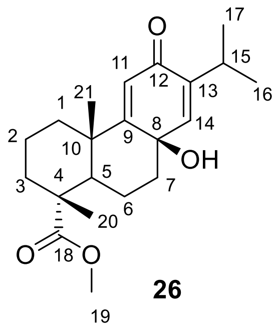

Colorless crystal, ^{1}H NMR (500 MHz, Chloroform-*d*) δ 6.37 (s, 1H, H-14), 6.00 (s, 1H, H-11), 3.64 (s, 3H, H-19), 2.88 (hept, *J* = 6.9 Hz, 1H, H-15), 2.10 – 1.95 (m, 2H), 1.83 – 1.65 (m, 5H), 1.63 – 1.44 (m, 3H), 1.39 (s, 3H, H-21), 1.28 (s, 3H, H-20), 1.06 (d, *J* = 6.9 Hz, 3H, H-16), 1.03 (d, *J* = 6.9 Hz, 3H, H-17). ^{13}C NMR (126 MHz, Chloroform-*d*) δ 187.19 (C=O, C-12), 178.58 (C=O, C-18), 168.01 (C, C-9), 145.27 (CH, C-14), 141.97 (C, C-13), 122.08 (CH, C-11), 69.29 (C-OH), 52.03 (CH_3, C-19), 48.79 (CH, C-5), 47.98 (C, C-10) , 40.47 (C, C-4), 39.21 (CH_2), 36.97 (CH_2), 36.82 (CH_2), 25.61 (CH, C-15), 21.77 (CH_3, C-20), 21.33 (CH_3, C-21), 20.56 (CH_3, C-16), 20.28 (CH_2), 17.81 (CH_2), 16.65 (CH_3, C-17). IR (film): 3446; 2926, 2870, 1726, 1664, 1632, 1460, 1388, 1250, 1179, 1132, 1104, 1045 cm^{-1}. HRMS (FAB-TOF) m/z: $[M + H]^+$ calcd for $C_{21}H_{30}O_4$, 347.2222; found, 347.2213.

Synthesis of 6-deoxytaxodione derivative 27

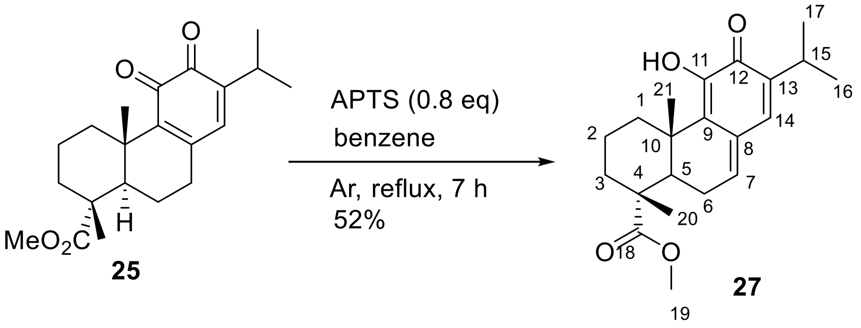

The compound **25** (50 mg, 0.145 mmol) was dissolved in benzene (8 mL), and then APTS (20 mg, 0.116 mmol) was added. After refluxing for 7 hours under argon atmosphere, the solution was concentrated. The residue was purified by silica gel column chromatography using a 10% EtOAc/hexane mixture to yield **27** (26 mg, 52%).

6-Deoxytaxodione derivative 27: ^{1}H NMR (500 MHz, Chloroform-*d*) δ 7.48 (s, 1H, H-14), 6.79 (s, 1H), 6.74 (dd, *J* = 6.9, 3.0 Hz, 1H, H-7), 3.68 (s, 3H, H-19), 3.08 (hept, *J* = 6.7 Hz, 1H, H-15), 3.06 – 3.01 (m, 2H), 2.55 – 2.38 (m, 1H), 2.20 – 2.00 (m, 1H), 1.87 – 1.53 (m, 5H), 1.32 (s, 3H, H-21), 1.23 (s, 3H, H-20), 1.16 (d, *J* = 6.7 Hz, 3H, H-16), 1.15 (d, *J* = 6.7

Hz, 3H, H-17). ^{13}C NMR (126 MHz, Chloroform-*d*) δ 181.34 (C=O, C-12), 178.47 (C=O, C-18), 147.69 (CH, C-11), 143.83 (C, C-13) , 140.71 (C, C-8), 136.12 (CH, C-14), 131.61 (C, C-9), 126.54 (C), 52.09 (CH_3, C-19), 47.30 (C, C-4), 45.01 (CH, C-5), 38.09 (C, C-10), 36.80 (CH_2), 35.96 (CH_2), 27.72 (CH_2), 26.62 (CH, C-15), 21.80 (CH_3, C-21), 21.46 (CH_3, C-20), 18.84 (CH_3, C-16), 18.20 (CH_2), 17.13 (CH_3, C-17). HRMS (FAB-TOF) m/z: [M + H]$^+$ calcd for $C_{20}H_{29}O_3$, 317.2117; found, 317.2106.

Synthesis of compounds 28 and 29.

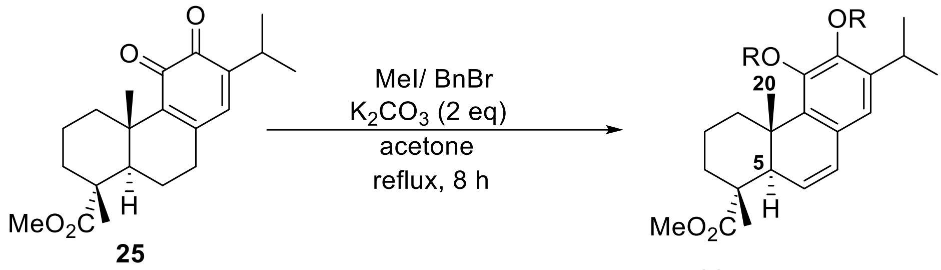

28 R = Me (92%)
29 R = Bn (90%)

The treatment of quinone **25** (100 mg**,** 0.290 mmol) with 0.638 mmol of MeI or (BnBr) in the presence of K_2CO_3 (88.2 mg, 0.638 mmol) in acetone, under reflux for 8 hours, leads to the complete transformation of the initial product. Subsequently, the solvent is evaporated, and the reaction is treated with water and ethyl acetate. The organic phase is collected and dried with Na_2SO_4. Separation by column chromatography filled with silica gel and a mixture of 10% AcOEt/hexane yields the pure product **28** (99.4 mg, 92%) or **29** (137 mg, 90%)

Methyl (1R,4aS,10aR)-7-isopropyl-5,6-dimethoxy-1,4a-dimethyl-1,2,3,4,4a,10a-hexahydrophenanthrene-1-carboxylate (28)

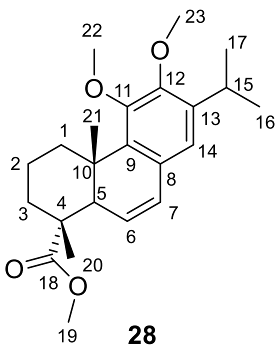

28

Colourless oil, ^{1}H NMR (500 MHz, Chloroform-*d*) δ 6.67 (s, 1H, H-14), 6.42 (dd, *J* = 9.5, 3.1 Hz, 1H, H-7), 5.58 (dd, *J* = 9.4, 2.8 Hz, 1H, H-6), 3.82 (s, 3H, H-22), 3.81 (s, 3H, H-23), 3.66

(s, 3H, H-19), 3.26 (hept, *J* = 7.1 Hz, 1H, H-15), 3.00 (t, *J* = 3.1 Hz, 1H, H-5), 2.95 (dd, *J* = 13.5, 3.7 Hz, 2H), 1.95 – 1.68 (m, 2H), 1.67 – 1.61 (m, 2H), 1.39 (s, 3H, H-21), 1.21 (dd, *J* = 24.0, 6.8 Hz, 6H, , H-17, H-16), 1.18 (s, 3H, H-20). ^{13}C NMR (126 MHz, Chloroform-*d*) δ 178.58 (C=O, C-18), 150.86 (C, C-11), 150.74 (C, C-12), 140.25 (C, C-13), 137.18 (C, C-9), 130.27 (C, C-8), 128.51 (CH, C-14), 128.36 (CH, C-6), 120.32 (CH, C-7), 60.37 (CH_3, C-22), 60.10 (CH_3, C-23), 52.01 (CH_3, C-19), 47.12 (C, C-10), 46.67 (CH, C-5), 40.49 (C, C-4), 36.04 (CH_2, C-3), 35.46 (CH_2, C-1), 26.52 (CH, C-15), 23.73 (CH_3, C-21), 23.16 (CH_3, C-16), 19.81 (CH_3, C-17), 18.90 (CH_2, C-2), 17.92 (CH_3, C-20). IR (film): 2930, 2867, 1726, 1461, 1446, 1383, 1299, 1243, 1125, 1085, 1021, 987, 877 cm^{-1}. HRMS (FAB-TOF) m/z: [M + H]$^+$ calcd for $C_{23}H_{32}O_4$, 373.2379; found, 373.2370

Methyl (1R,4aS,10aR)-5,6-bis(benzyloxy)-7-isopropyl-1,4a-dimethyl-1,2,3,4,4a,10a-hexahydrophenanthrene-1-carboxylate (29)

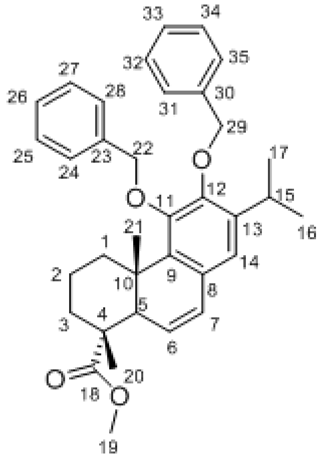

Colorless oil, ^{1}H NMR (500 MHz, Chloroform-*d*) δ 7.45 – 7.41 (m, 2H, H-32, H-35), 7.39 – 7.34 (m, 4H, H-24, H-26, H-27, H-28), 7.34 – 7.30 (m, 4H, H-31, H-33, , H-27 , H-34), 6.75 (s, 1H, H-14), 6.48 (dd, *J* = 9.4, 3.1 Hz, 1H, H-22), 5.64 (dd, *J* = 9.4, 3.1 Hz, 1H, H-29), 5.11 (d, *J* = 11.1 Hz, 1H, H-6), 5.08 (d, *J* = 11.1 Hz, 1H, H-7), 5.03 (d, *J* = 11.0 Hz, 1H), 4.92 (d, *J* = 11.0 Hz, 1H), 3.69 (s, 3H, H-19), 3.34 (hept, *J* = 6.9 Hz, 1H, H-15), 3.10 (br, 1H, H-5), 3.06 (q, *J* = 3.0 Hz, 2H), 2.01 (d, *J* = 4.1 Hz, 2H), 1.92 – 1.57 (m, 2H), 1.42 (s, 3H, H-21), 1.24 (s, 3H, H-20), 1.20 (dd, *J* = 27.7, 6.9 Hz, 6H, H-16, H-17). ^{13}C NMR (126 MHz, Chloroform-*d*) δ 178.61 (C=O, C-18), 149.58 (C, C-11), 140.86 (C, C-12), 137.90 (C, C-13), 137.68 (C, C-9), 137.66 (C, C-8), 130.81 (C, C-23), 128.62 (CH, C-26), 128.60 (CH, C-33), 128.39 (2 × CH, C-25, C-27), 128.35 (2 × CH, C-31, C-35), 128.15 (2 × CH, C-31, C-35), 127.92 (2 × CH, C-24, C-28), 127.83 (CH, C-14), 127.66 (CH), 120.77 (CH), 75.09 (CH_2, C-22), 74.84

(CH_2, C-29), 52.06 (CH_3, C-19), 47.13, 46.75 (CH), 40.60, 36.27 (CH_2), 35.48 (CH_2), 26.44 (CH), 23.72 (CH_3, C-21), 23.29 (CH_3, C-16), 20.02 (CH_3, C-17), 18.78 (CH_3, C-20), 17.99 (CH_2). IR (film): 2959, 2867, 1725, 1454, 1422, 1363, 1296, 1243, 1142, 1126, 1083, 1007, 752, 732, 696 cm^{-1}. HRMS (FAB-TOF) m/z: $[M + H]^+$ calcd for $C_{35}H_{40}O_4$, 525.3005; found, 525.3008

General procedure for the synthesis of compounds 33, and 34.

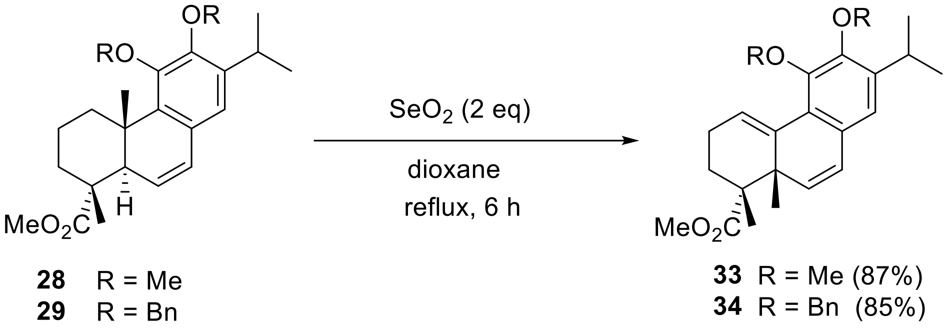

Two equivalents of SeO_2 were added to a solution of the mixture of compound **28** (120 mg, 0.322 mmol) or **29** (120 mg, 0.229 mmol) in dry 1,4-dioxane, and the mixture was stirred at reflux under an argon atmosphere for 6 hours. Following the same work-up procedure as in the general reference 2, the product **33** (103.8 mg, 87%) or **34** (101.8 mg, 85%) was obtained after column chromatography using a 10% EtOAc/hexane mixture.

Methyl (1R,10aS)-7-isopropyl-5,6-dimethoxy-1,10a-dimethyl-1,2,3,10a-tetrahydrophenanthrene-1-carboxylate (33)

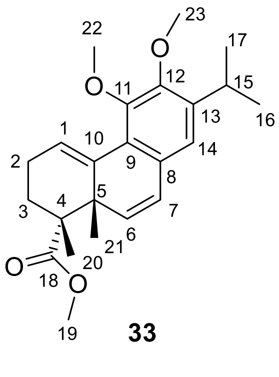

33

^{1}H NMR (400 MHz, Chloroform-*d*) δ 6.64 (s, 1H, H-14), 6.58 (br, 1H, H-1), 6.25 (d, *J* = 9.7 Hz, 1H, H-6), 5.97 (d, *J* = 9.7 Hz, 1H, H-7), 3.86 (s, 3H, H-22), 3.75 (s, 3H, H-23), 3.64 (s, 3H, H-19), 3.28 (hept, *J* = 6.9 Hz, 1H, H-15), 2.31 (ddt, *J* = 13.4, 5.2, 3.2 Hz, 2H, H-2), 2.07 – 1.89 (m, 1H, H-3), 1.59 (m, 1H, H-3), 1.37 (s, 3H, H-21), 1.24 (d, *J* = 6.9 Hz, 3H, H-16), 1.20 (d, *J* = 6.9 Hz, 3H, H-17), 1.17 (s, 3H, H-20). ^{13}C NMR (101 MHz, Chloroform-*d*) δ

176.38 (C=O, C-18), 151.51 (C, C-11), 150.52 (C, C-12), 140.79 (C, C-13), 134.64 (C, C-9), 134.27 (CH, C-14), 129.28 (C, C-8), 127.08 (CH, C-1), 124.37 (C, C-10), 123.48 (CH, C-6), 119.64 (CH, C-7), 61.13 (CH_3, C-22), 59.70 (CH_3, C-23), 51.50 (CH_3, C-19), 47.45 (C, C-5), 41.30 (C, C-4), 29.91 (CH_2, C-2), 26.72 (CH, C-15), 24.27 (CH_2, C-2), 23.69 (CH_3, C-21), 23.23 (CH_3, C-20), 22.55 (CH_3, C-16), 20.83 (CH_3, C-17). IR (film): 2960, 1779, 1723, 1468, 1447, 1416, 1393, 1331, 1299, 1258, 1161,1118, 1057, 999, 878, 763 cm^{-1}. HRMS (FAB-TOF) m/z: $[M + H]^+$ calcd for $C_{23}H_{30}O_4$, 371.2222; found, 371.2223

Methyl (1R,10aS)-5,6-bis(benzyloxy)-7-isopropyl-1,10a-dimethyl-1,2,3,10a-tetrahydrophenanthrene-1-carboxylate (34)

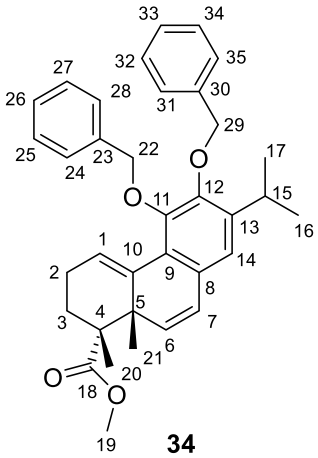

34

^{1}H NMR (500 MHz, Chloroform-*d*) δ 7.33 (d, *J* = 1.4 Hz, 2H, Har), 7.31 – 7.21 (m, 4H, Har), 7.19 (s, 2H, Har), 6.63 (dd, *J* = 5.1, 3.1 Hz, 1H, H-1), 6.60 (s, 1H, H-14), 6.18 (d, *J* = 9.7 Hz, 1H, H-6), 5.90 (d, *J* = 9.7 Hz, 1H, H-7), 5.21 (d, *J* = 11.0 Hz, 1H, H-22), 4.85 (d, *J* = 10.6 Hz, 1H, H-29), 4.75 (d, *J* = 10.6 Hz, 1H, H-29), 4.70 (d, *J* = 11.0 Hz, 1H, H-22), 3.55 (s, 3H, H-19), 3.24 (hept, *J* = 6.9 Hz, 1H, H-15), 2.31 – 2.20 (m, 1H), 2.11 (dd, *J* = 19.0, 4.4 Hz, 1H), 1.92 – 1.83 (m, 2H), 1.28 (s, 3H, H-21), 1.14 (d, *J* = 6.9 Hz, 3H, H-16), 1.05 (d, *J* = 8.1 Hz, 3H, H-17), 1.04 (s, 3H, H-20). ^{13}C NMR (126 MHz, Chloroform-*d*) δ 175.30 (C=O, C-18), 149.44 (C, C-11), 148.36 (C, C-12), 140.24 (C, C-13), 136.97 (C, C-9), 136.91 (C, C-8), 133.47 (CH, C-14), 133.38 (C, C-30), 128.53 (C, C-23), 127.50 (2 × CH, C-25, C-27), 127.35 (2 × CH, C-32, C-34), 127.24 (2 × CH, C-24, C-28), 127.22 (2 × CH, C-26, C-33) , C-31, C-35, 127.12 (CH, C-6), 126.81 (CH, C-7), 126.79 (CH, C-1), 124.02 (C, C-10), 122.33 (CH), 118.86 (CH, C-1), 74.40 (CH_2, C-22), 73.89 (CH_2, C-29), 50.51 (CH_3, C-19), 46.42 (C), 40.29 (C), 28.95 (CH_2), 25.56 (CH_3, C-21), 23.21 (CH_2), 22.52 (CH_3, C-20), 22.51 (CH_3, C-16), 21.39 (CH, C-15), 19.81 (CH_3, C-17). IR (film): 2960, 1720, 1454, 1426, 1360, 1328,

1294, 1258, 1160, 1117, 1079, 1028, 972, 801, 733, 696 cm^{-1}. HRMS (FAB-TOF) m/z: $[M + H]^+$ calcd for $C_{35}H_{38}O_4$, 523.2848; found, 523.2847

General procedure for the synthesis of compounds 30, and 31.

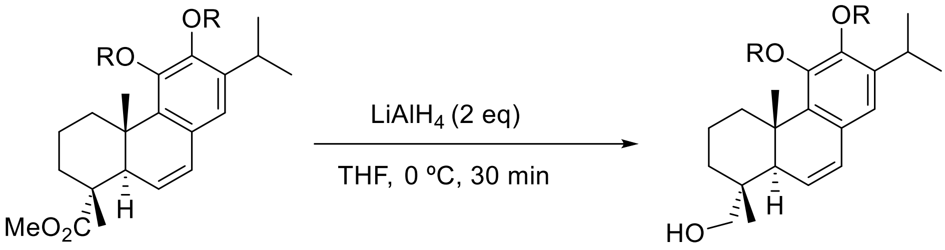

28 R = Me
29 R = Bn

30 R = Me (98%)
31 R = Bn (97%)

Dissolve the compound **28** (120 mg, 0.322 mmol) or **29** (120 mg, 0.229 mmol) in dry THF (10 ml) and cool to 0°C. Two equivalents of $LiAlH_4$ were added gradually, and the mixture was stirred for 30 minutes. After confirming by TLC that no starting material remained, the reaction mixture was slowly poured over ice, and ethyl ether was added and stirred for ten minutes. Afterward, the solution was cooled again to 0°C, and concentrated HCl was added until the pH became acidic, stirring for an additional fifteen minutes. After this time, the phases were separated, and the organic phase was washed with water (3 x 5 ml) and brine (2 x 6 ml) before being dried over Na_2SO_4. Filter and evaporate the solvent to obtain product of **30** (108.7 mg ,98%) or (**31** (110.3 mg, 97%)) as an oily substance.

((1R,4aS,10aR)-7-isopropyl-5,6-dimethoxy-1,4a-dimethyl-1,2,3,4,4a,10a-hexahydrophenanthren-1-yl) methanol (30)

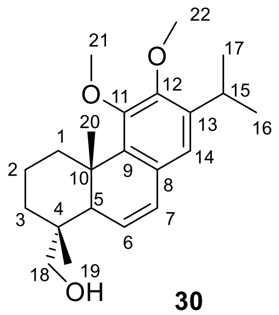

30

^{1}H NMR (500 MHz, Chloroform-*d*) δ 6.68 (s, 1H, H-14), 6.47 (dd, *J* = 9.6, 3.1 Hz, 1H, H-7), 5.87 (dd, *J* = 9.6, 3.1 Hz, 1H, H-6), 3.82 (s, 3H, H-22), 3.81 (s, 3H, H-21), 3.51 (d, *J* = 11.1 Hz, 1H, H-18), 3.26 (d, *J* = 11.1Hz, 1H, H-18), 3.24 (hept, *J* = 6.9 Hz, 1H, H-15), 3.09 – 2.66 (m, 2H), 2.45 (t, *J* = 3.1 Hz, 1H, 1.85 (s, 3H, H-20), 1.89 – 1.46 (m, 4H), 1.24 (d, *J* = 6.9 Hz,

3H, H-17), 1.18 (d, *J* = 6.9 Hz, 3H, H-16), 1.01 (s, 3H, H-19). ^{13}C NMR (126 MHz, Chloroform-*d*) δ 150.85 (C, C-11), 150.78 (C, C-12), 140.12 (C, C-13), 137.76 (C, C-9), 130.33 (C, C-8), 128.75 (CH, C-14), 127.18 (CH, C-6), 120.21 (CH, C-7), 71.69 (CH_2), 60.39 (O-CH_3, C-21), 60.12 (O-CH_3, C-22), 45.28 (CH, C-5), 40.86 (C), 37.60 (C), 36.26 (CH_2), 34.18 (CH_2), 26.51 (CH, C-15), 23.75 (CH_3, C-19), 23.17 (CH_3, C-17), 19.73 (CH_3, C-16), 18.74 (CH_2), 18.25 (CH_3, C-20). IR (film): 3396; 2958; 2925; 2865, 1726, 1461, 1445, 1378, 1297, 1227, 1065, 1020, 986, 876, 630 cm^{-1}. HRMS (FAB-TOF) m/z: $[M + H]^+$ calcd for $C_{22}H_{32}O_3$, 345.2430; found, 345.2423

((1R,4aS,10aR)-5,6-bis(benzyloxy)-7-isopropyl-1,4a-dimethyl-1,2,3,4,4a,10a-hexahydrophenanthren-1-yl) methanol (31)

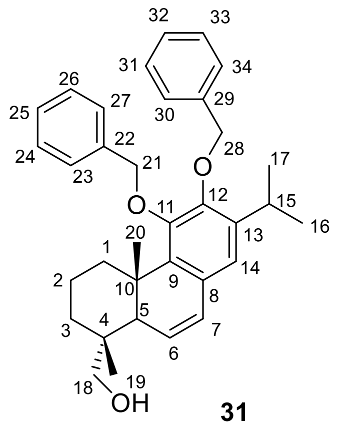

31

^{1}H NMR (500 MHz, Chloroform-*d*) δ 7.45 – 7.41 (m, 2H, H-30 , H-34), 7.38 – 7.33 (m, 4H), 7.32 – 7.30 (m, 4H), 6.75 (s, 1H, H-14), 6.52 (dd, *J* = 9.6, 3.1 Hz, 2H, H-6), 5.92 (dd, *J* = 9.6, 2.9 Hz, 2H, H-7), 5.08 (s, 2H, H-21), 5.03 (d, *J* = 11.0 Hz, 1H, , H-28), 4.90 (d, *J* = 11.0 Hz, 1H, H-28), 3.52 (s, 1H), 3.34 (hept, *J* = 6.9 Hz, 1H, H-15), 3.28 (d, *J* = 11.2 Hz, 1H, H-18), 3.08 (dt, *J* = 13.3, 3.7 Hz, 1H, H-18), 2.51 (t, *J* = 3.0 Hz, 1H), 1.94 – 1.85 (m, 1H), 1.71 (m, 1H), 1.61 (s, 1H), 1.52 (td, *J* = 13.1, 4.1 Hz, 1H), 1.44 – 1.35 (m, 1H), 1.24 (s, 3H, H-20), 1.22 (d, *J* = 6.9 Hz, 3H, H-17), 1.16 (d, *J* = 6.9 Hz, 3H, H-16), 1.02 (s, 3H, H-19). ^{13}C NMR (125 MHz, Chloroform-*d*) δ 149.62 (C), 140.70 (C), 138.24 (C), 137.96 (C), 137.72 (C), 130.85 (C), 128.85 (C), 128.37 (2 × CH), 128.33 (2 × CH), 128.15 (2 × CH), 127.94 (2 × CH), 127.80 (CH), 127.62 (CH), 127.43 (CH), 120.64 (CH), 75.07 (O-CH_2), 74.84 (O-CH_2, C-28), 71.69 (O-CH_2, C-21), 45.32 (CH), 40.95 (C), 37.64 (C), 36.52 (CH_2), 34.18 (CH_2), 26.43 (CH, C-15), 23.73 (CH_3), 23.30 (CH_3), 19.93 (CH_3), 18.62 (CH_2), 18.32 (CH_3). IR (film): 1371, 3033, 2960, 2925, 2866, 1497, 1454, 1421, 1363, 1294, 1217, 1058, 1008, 750, 696 cm^{-1}. HRMS (FAB-TOF) m/z: $[M + H]^+$ calcd for $C_{34}H_{40}O_3$, 497.3056; found, 497.3053

Treatment of orthoquinone 25 with Ac_2O and cat. $Sc(OTf)_3$.

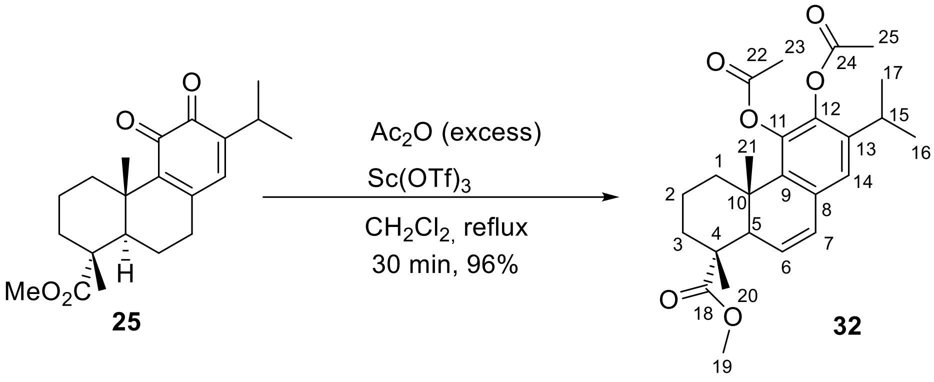

Compound 32: ^{1}H NMR (500 MHz, Chloroform-*d*) δ 6.81 (s, 1H, H-16), 6.41 (dd, *J* = 9.5, 3.1 Hz, 1H, H-7), 5.59 (dd, *J* = 9.5, 2.8 Hz, 1H, H-6), 3.58 (s, 3H, H-19), 2.81 (hept, *J* = 6.9 Hz, 1H, H-15), 2.22 (s, 3H, H-25), 2.21 (s, 3H, H-23), 1.71 (td, *J* = 8.7, 3.1 Hz, 2H), 1.66 (d, *J* = 3.1 Hz, 1H), 1.65 – 1.59 (m, 2H), 1.58 – 1.47 (m, 2H), 1.29 (s, 3H, H-21), 1.13 (d, *J* = 6.9 Hz, 3H, H-17), 1.09 (d, *J* = 6.9 Hz, 3H, H-16), 1.08 (s, 3H, H-20). ^{13}C NMR (125 MHz, Chloroform-*d*) δ 178.23 (C=O, C-18), 168.96 (C=O, C-22), 168.53 (C=O, C-24), 139.64 (C, C-9), 139.36 (C, C-13), 139.21 (C, C-11), 136.17 (C, C-12), 133.05 (C, C-8), 130.07 (CH, C-14), 128.12 (CH, C-7), 122.86 (CH, C-6), 52.11 (CH, C-5), 46.86 (C, C-4), 45.91 (CH_3), 40.21 (C, C-10), 35.02 (CH_2, C-1), 27.36 (2 × CH_3, C-16, C-17), 23.09 (CH_3, C-21), 22.64 (CH, C-15), 21.02 (CH_3, C-25), 20.43 (CH_3, C-23), 18.67 (2×CH_2, C-2, C-21), 17.89 (CH_3). IR (film): 2964, 1772, 1725, 1370, 1248, 1207, 1178, 1141, 1123, 1013, 882 cm^{-1}. HRMS (FAB-TOF) m/z: $[M + H]^+$ calcd for $C_{25}H_{32}O_6$, 429.2277; found, 429.2268

$Sc(OTf)_3$ (10 mg) was introduced into a solution containing quinone **25** (130 mg, 0.377 mmol) in anhydrous CH_2Cl_2 (5 mL) and acetic anhydride (1 mL, 10.6 mmol). The mixture underwent reflux for 30 minutes, during which TLC indicated the absence of any remaining starting material. Subsequently, the reaction was allowed to cool to room temperature, CH_2Cl_2 (15 mL) was introduced, and the organic phase underwent washing with H_2O (3 × 10 mL). After drying over anhydrous Na_2SO_4 and evaporation, a crude product was obtained. Column chromatography on silica gel with a 20% EtOAc/hexane mixture yielded product **32** (155 mg, 96%).

Synthesis of 35.

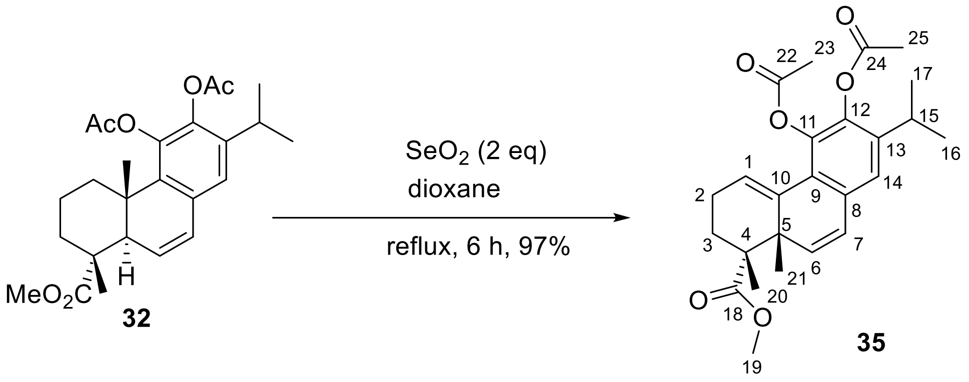

The compound **35** was synthesized from compound **32** (100 mg, 0.233 mmol) through the action of SeO_2 (51.7 mg, 0.446 mmol) following the same procedure as the synthesis of compounds **33** and **34**, with a significant yield (96.4 mg, 97%).

Compound 35: ^{1}H NMR (500 MHz, Chloroform-*d*) δ 6.75 (s, 1H, H-14), 6.19 (d, *J* = 9.7 Hz, 1H, H-7), 6.15 (s, 1H, H-1), 5.94 (d, *J* = 9.7 Hz, 1H, H-6), 3.53 (s, 3H, H-19), 2.85 (hept, *J* = 6.9 Hz, 1H, H-15), 2.23 (s, 3H, H-25), 2.22 – 2.18 (m, 1H), 2.13 (s, 3H, H-23), 2.11 – 1.94 (m, 2H), 1.86 – 1.78 (m, 2H), 1.71 – 1.53 (m, 1H), 1.25 (s, 3H, H-20), 1.19 (d, *J* = 1.4 Hz, 1H), 1.15 (d, *J* = 6.9 Hz, 3H, H-17), 1.09 (d, *J* = 6.9 Hz, 3H, H-16), 1.03 (s, 3H, H-21). ^{13}C NMR (126 MHz, Chloroform-*d*) δ 176.23 (C=O, C-18), 168.63 (C=O, C-22), 168.02 (C=O, C-24), 140.11 (C, C-9), 139.91 (C, C-12), 139.76 (C, C-11), 136.02 (CH, C-6), 134.78 (C, C-10), 131.58 (C, C-13), 126.89 (CH, C-7), 125.19 (C, C-8), 122.66 (CH, C-1), 121.62 (CH, C-14), 51.56 (CH_3, C-19), 47.23 (C, C-4), 41.50 (C, C-5), 29.74 (CH_2, C-3), 27.48 (CH, C-15), 24.35 (CH_2, C-2), 23.03 (CH_3, C-16), 22.71 (CH_3, C-17), 22.16 (CH_3, C-21), 20.88 (CH_3, C-25), 20.75 (CH_3, C-23), 20.44 (CH_3, C-20). IR (film): 2965, 1770, 1736, 1430, 1368, 1203, 1183, 1117, 1034, 880, 796, 744 cm^{-1}. HRMS (FAB-TOF) m/z: $[M + H]^+$ calcd for $C_{25}H_{31}O_6$, 427.2121; found, 427.2119

Synthesis of derivative of Prattinin A (37)

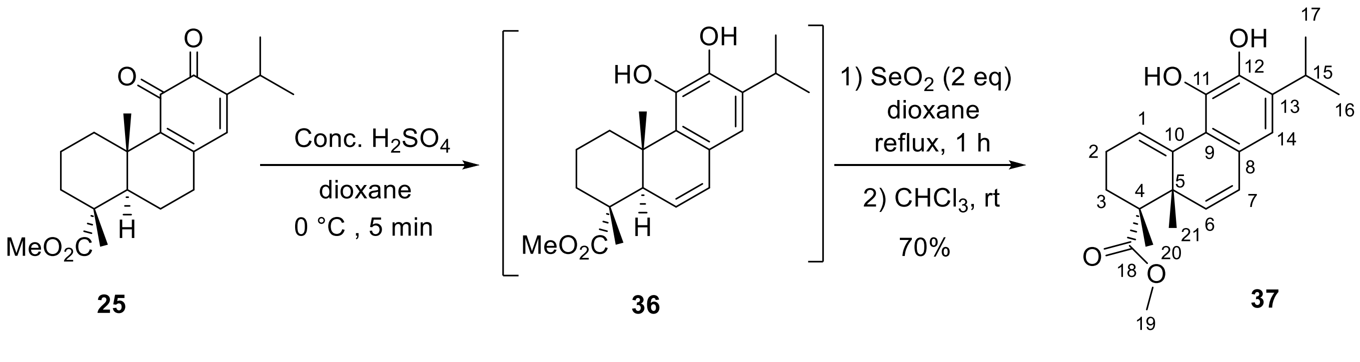

The synthesis of the intermediate **36** (unstable) from quinone **25** (120 mg, 0.348 mmol) was achieved through the action of sulfuric acid in dioxane, quantitatively, following the same protocol described in our previous work (ref. 2). This intermediate was immediately transformed in the subsequent reaction by the action of SeO_2 (77.3 mg, 0.697 mmol) in dry dioxane at reflux for 2 hours. The reaction resulted in the complete transformation of the product, and the reaction mixture was treated with saline water and ethyl acetate. After decantation and drying with Na_2SO_4, filtration and solvent concentration yielded a crude product. The crude product was dissolved in $CHCl_3$ and stirred for 2 hours at room temperature. Solvent concentration and separation by column chromatography filled with silica gel using a 15% AcOEt/hexane mixture allowed the isolation of product **37** (83.4 mg, 70%) in its pure state.

Derivative of Prattinin A 37: ^{1}H NMR (400 MHz, Chloroform-*d*) δ 1.10 (s, 3H, H-21), 1.21 (dd, *J* = 13.4, 7.0 Hz, 6H, H-16, H-17), 1.33 (s, 3H, H-20), 1.98 – 1.87 (m, 2H), 2.39 – 2.19 (m, 2H), 3.19 (hept, *J* = 6.9 Hz, 1H, H-15), 3.59 (s, 3H, H-19), 5.79 (d, *J* = 9.7 Hz, 1H, H-7), 6.12 (t, *J* = 3.9 Hz, 1H, H-1), 6.20 (d, *J* = 9.7 Hz, 1H, H-6), 6.44 (s, 1H, H-14). ^{13}C NMR (101 MHz, Chloroform-*d*) δ 20.74 (CH_3, C-16), 22.18 (CH_3, C-17), 22.29 (CH_3, C-21), 22.55 (CH_3, C-20), 24.06 (CH_2, C-3), 26.99 (CH, C-15), 29.88 (CH_2, C-2), 41.68 (C, C-4), 47.10 (C, C-5), 51.49 (CH_3, C-19), 116.17 (CH, C-14), 116.42 (C, C-9), 123.79 (CH, C-1), 123.86 (CH, C-7), 125.14 (C, C-8), 132.01 (CH, C-6), 133.13 (C, C-13), 138.28 (C, C-10), 140.27 (C, C-11), 141.37 (C, C-12), 176.04 (C, C-18). IR (film): 3463, 2957, 2926, 2870, 1722, 1439, 1326, 1341, 1264, 1221, 1190, 1164, 1119, 1026, 760, 746 cm^{-1}. HRMS (ESI) m/z: calcd for $C_{21}H_{27}O_4$ (M+H+) 343.1909, found: 343.1908.

Synthesis of Prattinin A (9)

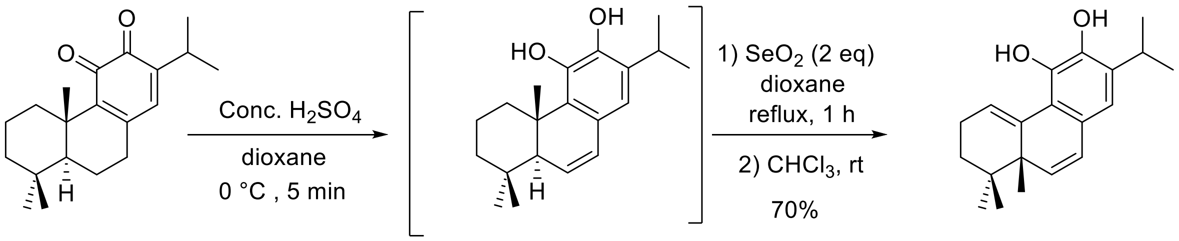

39 40 9

The synthesis of Prattinin A (**9**)[5] from quinone **39** (150 mg, 0.50 mmol), with a good yield (112 mg, 75%), was carried out using the same synthesis protocol as its analog **37** described above.

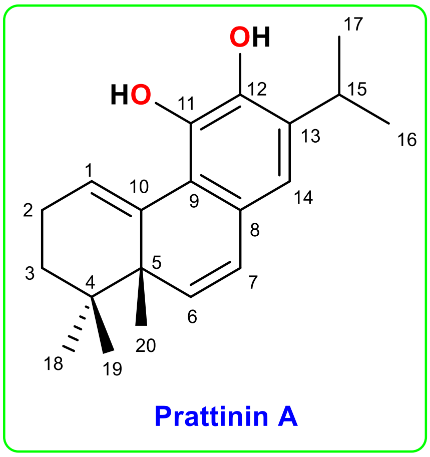

Table S2. ^{1}**H NMR data for the synthetic and natural Prattinin A (9)**

Position	Natural **Prattinin A** (**9**) (Ref. 6, 500 MHz, CDCl3)	Synthetic **Prattinin A** (**9**) (500 MHz, CDCl3)
1	6.08 (dd, 6.3, 2.9)	6.11 – 6.09 (m, 1H)
2α	.34 (dddd, 19.5, 13.5, 6.3, 2.9)	2.39 – 2.17 (m, 1H)
2β	2.20 (br dt, 19.5, 6.3)	2.21 (dt, *J* = 19.1, 5.0 Hz, 1H)
3α	1.31 (br dd, 13.5, 6.3)	1.31 (dd, *J* = 13.3, 6.4 Hz, 1H)
3β	1.88 (td, 13.5, 6.3)	1.88 (td, *J* = 12.6, 6.3 Hz, 1H)
4	-	-
5	-	-
6	6.22 (d, 9.5)	6.23 (d, *J* = 9.7 Hz, 1H)
7	5.49 (d, 9.5)	5.81 (d, *J* = 9.7 Hz, 1H)
8	-	-
9	-	-
10	-	-
11	-	-
12	-	-
13	-	-

14	6.45, s	6.45 (s, 1H)
15	3.23 (septet, 6.9)	3.23 (sept, *J* = 6,9 Hz, 1H)
16	1.22 (d, 6.9)	1.22 (d, *J* = 6.9 Hz, 3H)
17	1.25 (d, 6.9)	1.25 (d, *J* = 7.0 Hz, 3H)
18	0.95 (s)	0.96 (s, 3H)
19	1.03 (s)	1.04 (s, 3H)
20	1.06 (s)	1.06 (s, 3H)
OH-11	5.72 (br s)	5.75 (s, 1H)
OH-12	5.53 (br s)	5.56 (s, 1H)

Table S3. ^{13}C NMR data for the synthetic and natural Prattinin A (9)

Position	Natural **Prattinin A** (**9**) Ref 6, 125 MHz, $CDCl_3$)	Synthetic **Prattinin A** (**9**) (125 MHz, $CDCl_3$)
1	124.6	124.58
2	23.6	23.54
3	32.3	32.32
4	33.0	32.96
5	43.1	43.07
6	132.1	132.05
7	124.8	124.80
8	125.1	125.08
9	116.5	116.56
10	138.6	138.52
11	140.2	140.23
12	141.5	141.52
13	133.1	133.10
14	115.8	115.79
15	27.0	27.00
16	22.2	22.22
17	22.6	22.59
18	24.7	24.71
19	24.9	24.91
20	21.8	21.80

Note: The assignment of the molecules is based on the NMR analyses we conducted, as well as the NMR data of molecules similar to those we synthesized, cited in the bibliography, including those presented below:

[1] Ait El Had, M.; Guardia, J.J.; Ramos, J.M.; Taourirte, M.; Chahboun, R.; Alvarez-Manzaneda, E. Bioinspired Synthesis of Pygmaeocins and Related Rearranged Abietane Diterpenes: Synthesis of Viridoquinone. *Org. Lett*. **2018**, 20, 5666–5670.

[2] Machumi, F.; Samoylenko, V.; Yenesew, A.; Derese, S.; Midiwo, J.O.; Wiggers, F.T.; Ilias, M. Antimicrobial and Antiparasitic Abietane Diterpenoids from the Roots of *Clerodendrum eriophyllum*. *Nat. Prod. Commun.* **2010**, *5*, 853–858.

[3] González, M.A. Synthetic Derivatives of Aromatic Abietane Diterpenoids and Their Biological Activities. *Eur. J. Med. Chem.* **2014**, *87*, 834–842.

[4] Kawabe, H.; Suzuki, R.; Hirota, H.; Matsuzaki, K.; Gong, X.; Ohsaki, A. A New Diterpenoid with a Rearranged Skeleton from *Salvia prattii*. *Nat. Prod. Commun*. **2017**, *12*, 1177–1179.

References :

1. Tanaka, N.; Yamada, K.; Shimomoto, Y.; Tsuji, D.; Itoh, K.; Kawazoe, K.; Kashiwada, Y. Lophachinins A–E, Abietane Diterpenes from a Mongolian Traditional Herbal Medicine *Lophanthus chinensis*. *Fitoterapia* **2020**, *146*, 104702.

2. Ait El Had, M.; Guardia, J.J.; Ramos, J.M.; Taourirte, M.; Chahboun, R.; Alvarez-Manzaneda, E. Bioinspired Synthesis of Pygmaeocins and Related Rearranged Abietane Diterpenes: Synthesis of Viridoquinone. *Org. Lett*. **2018**, 20, 5666–5670.

3. Wu, W.M.; Liu, Y.; Chen, X.; Jin, A.; Zhou, M.; Tian, T.; Ruan, H.L. Diterpenoids from the Branch and Leaf of *Abies fargesii*. *Fitoterapia* **2016**, *110*, 123–128.

4. González-Cardenete, M.A.; Hamulic, D.; Miquel-Leal, F.J.; González-Zapata, N.; Jimenez-Jarava, O.J.; Brand, Y.M.; Marín, M.L. Antiviral Profiling of C-18-or C-19-Functionalized Semisynthetic Abietane Diterpenoids. *J. Nat. Prod*. **2022**, *85*, 2044–2051.

5. Domingo, V.; Prieto, C.; Silva, L.; Rodilla, J.M.; Quilez del Moral, J.F.; Barrero, A.F. Iodine, a Mild Reagent for the Aromatization of Terpenoids. *J. Nat. Prod*. **2016**, *79*, 831–837.

6. Kawabe, H.; Suzuki, R.; Hirota, H.; Matsuzaki, K.; Gong, X.; Ohsaki, A. A New Diterpenoid with a Rearranged Skeleton from *Salvia prattii*. *Nat. Prod. Commun*. **2017**, *12*, 1177–1179.

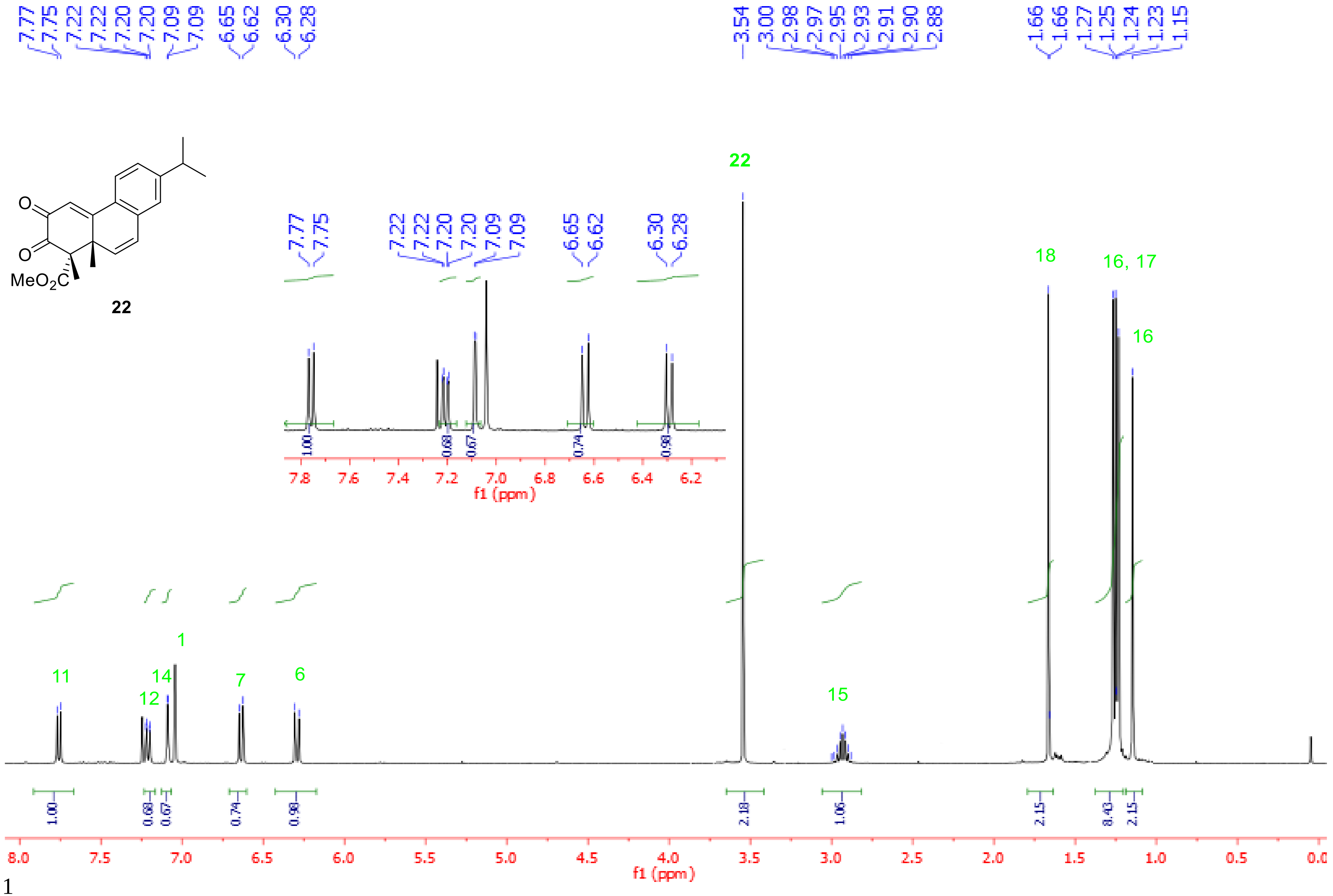

7.77
7.75
7.22
7.22
7.20
7.20
7.09
7.09
6.65
6.62
6.30
6.28
3.54
3.00
2.98
2.97
2.95
2.93
2.91
2.90
2.88
1.66
1.66
1.27
1.25
1.24
1.23
1.15
MeO2C
22
11
12
14
1
7
6
15
18
16, 17
16
1.00
0.68
0.67
0.74
0.98
2.18
1.06
2.15
8.43
2.15
f1 (ppm)

1

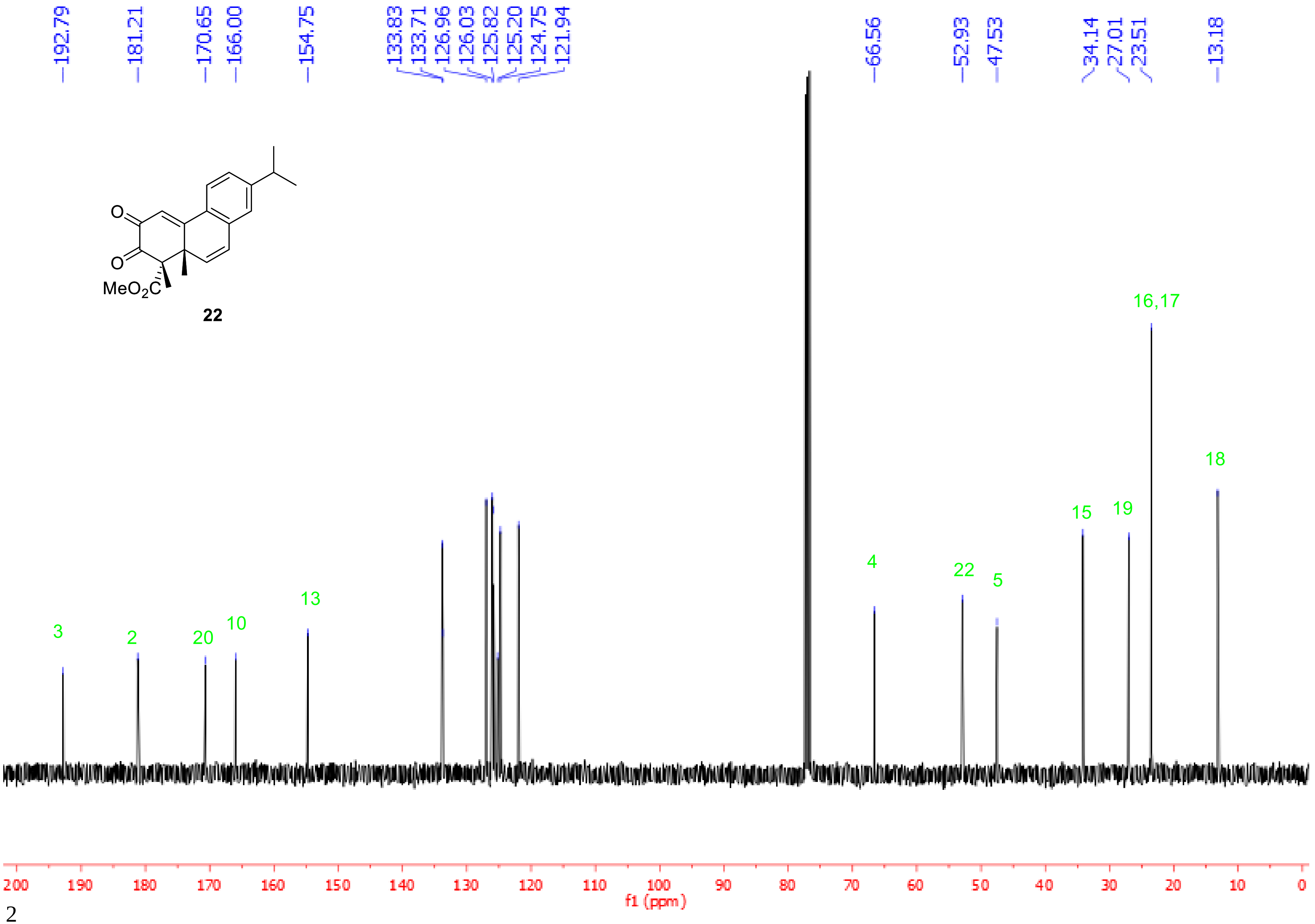
192.79
181.21
170.65
166.00
154.75
133.83
133.71
126.96
126.03
125.82
125.20
124.75
121.94
66.56
52.93
47.53
34.14
27.01
23.51
13.18
O
O
MeO2C
22
3
2
20
10
13
4
22
5
15
19
16,17
18
f1 (ppm)

2

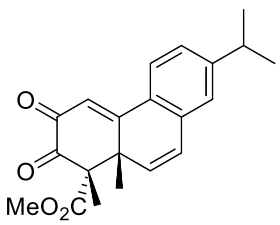

22

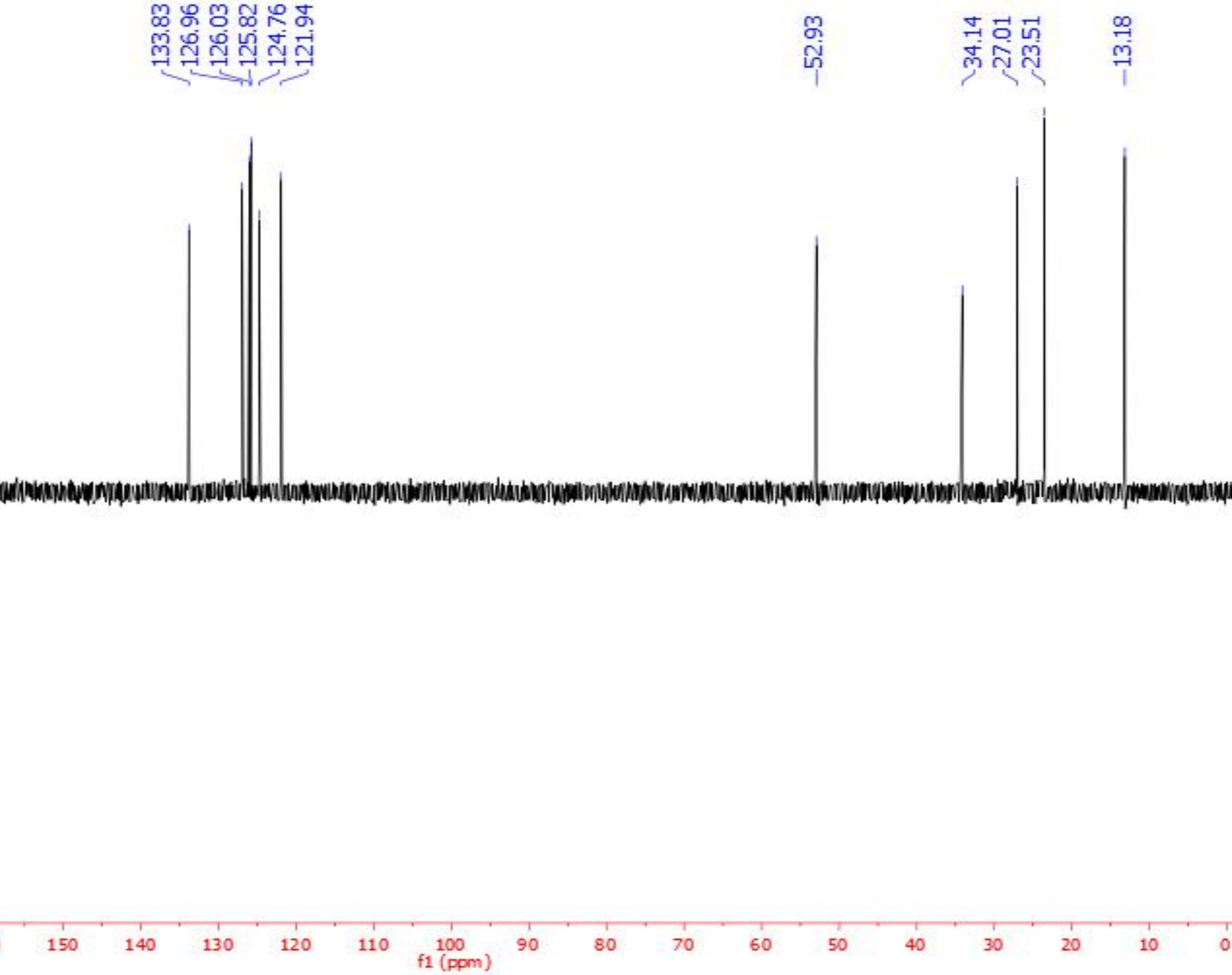
133.83
126.96
126.03
125.82
124.76
121.94
52.93
34.14
27.01
23.51
13.18
200 190 180 170 160 150 140 130 120 110 100 90 80 70 60 50 40 30 20 10 0
f1 (ppm)

3

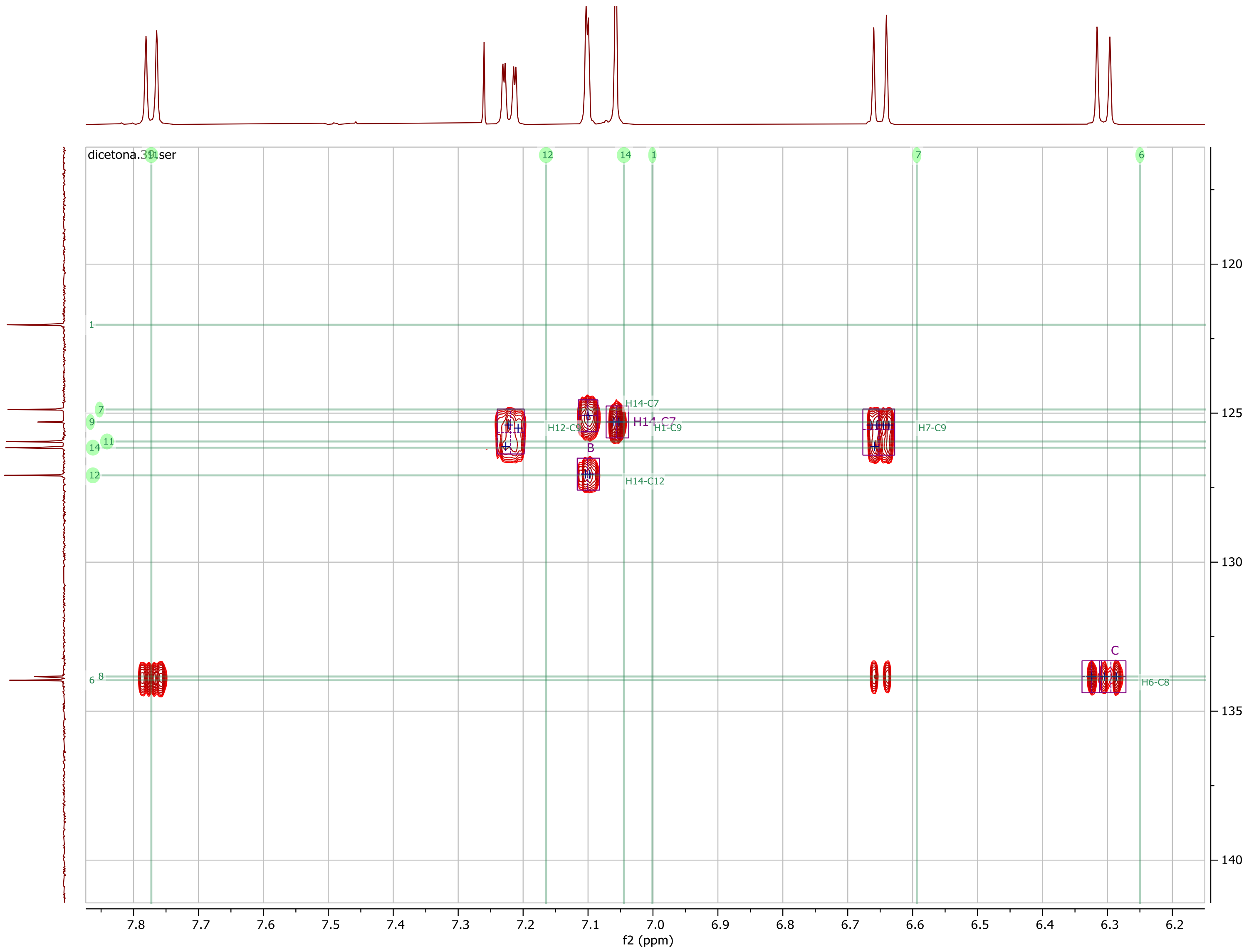
dicetona.3
f2 (ppm)
f1 (ppm)
H14-C7
H12-C9
H1-C9
H7-C9
H14-C12
H6-C8
B
C
7.8
7.7
7.6
7.5
7.4
7.3
7.2
7.1
7.0
6.9
6.8
6.7
6.6
6.5
6.4
6.3
6.2
120
125
130
135
140

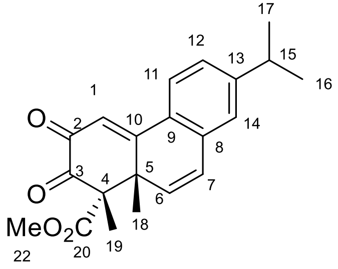
MeO2C
O
O

4

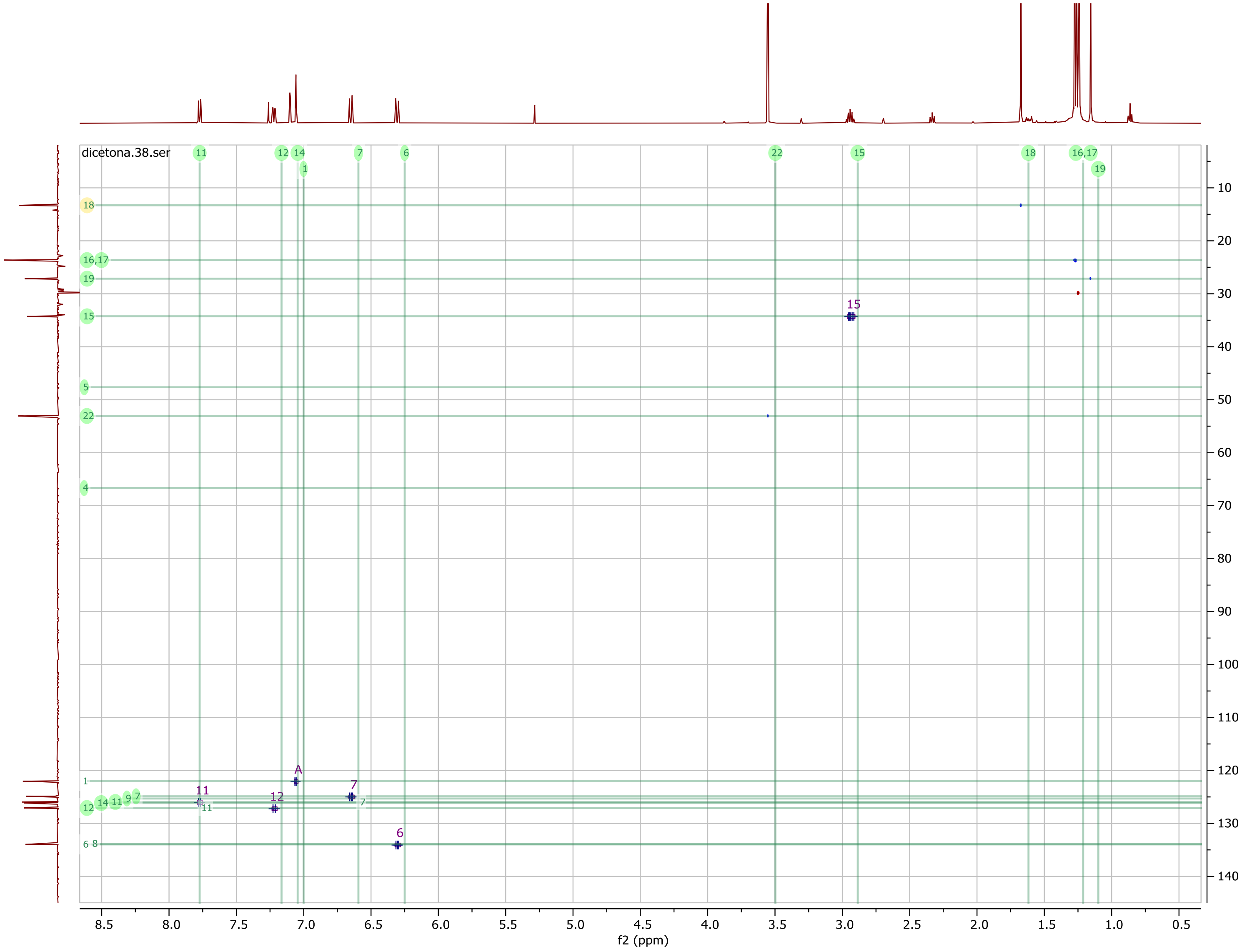

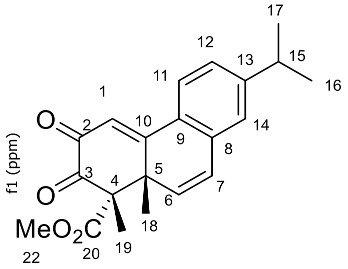

5

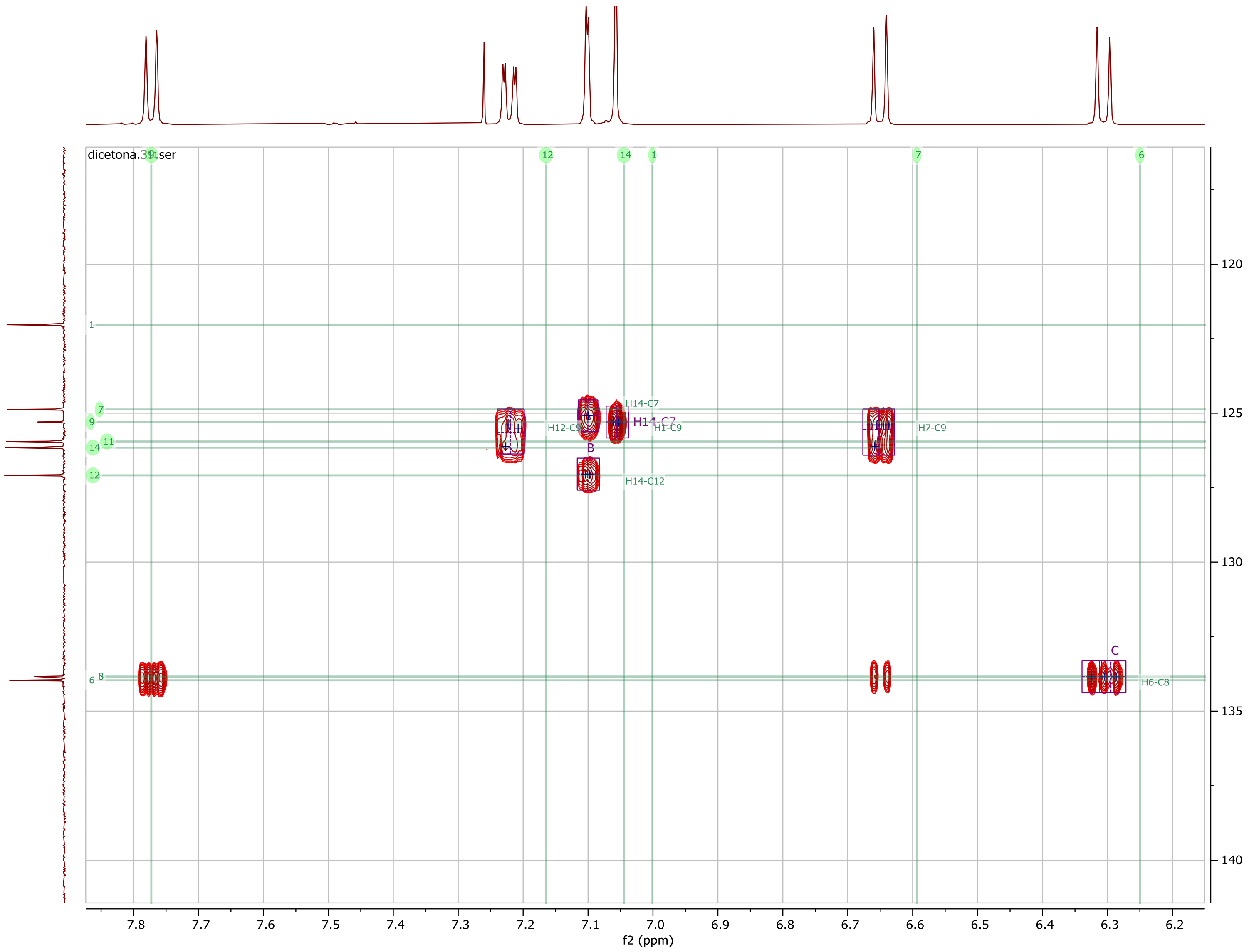
dicetona.3?.ser
H14-C7
H12-C9
H1-C9
H7-C9
H14-C12
H6-C8
f2 (ppm)
f1 (ppm)

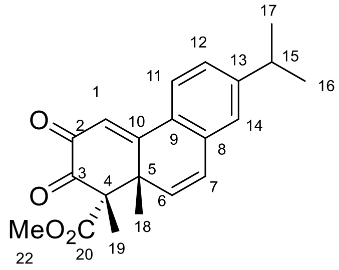
MeO2C

6

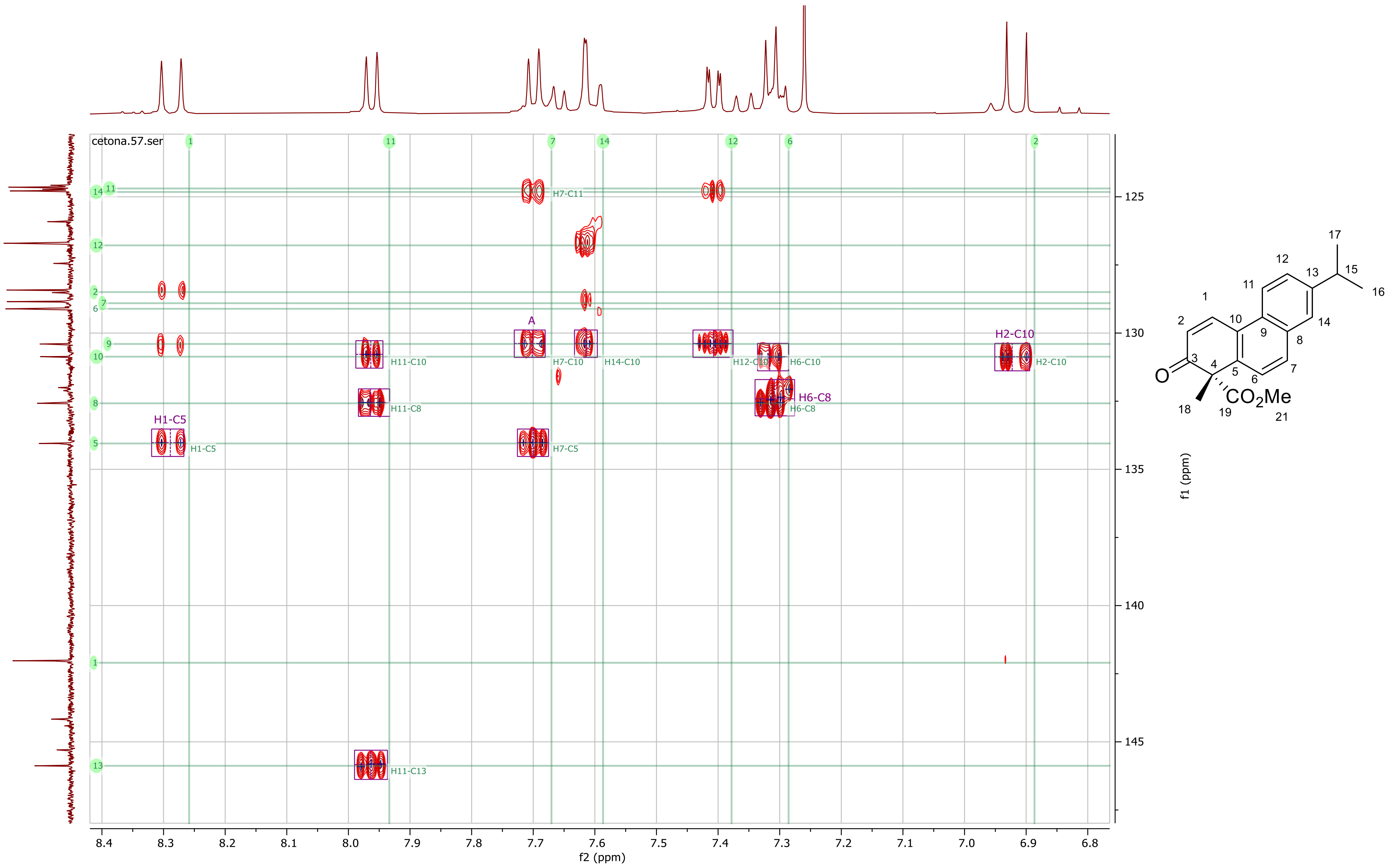
cetona.57.ser
H1-C5
H7-C11
H7-C10
H14-C10
H11-C10
H11-C8
H12-C10
H6-C10
H6-C8
H2-C10
H7-C5
H11-C13
A
f2 (ppm)
f1 (ppm)
CO2Me

7

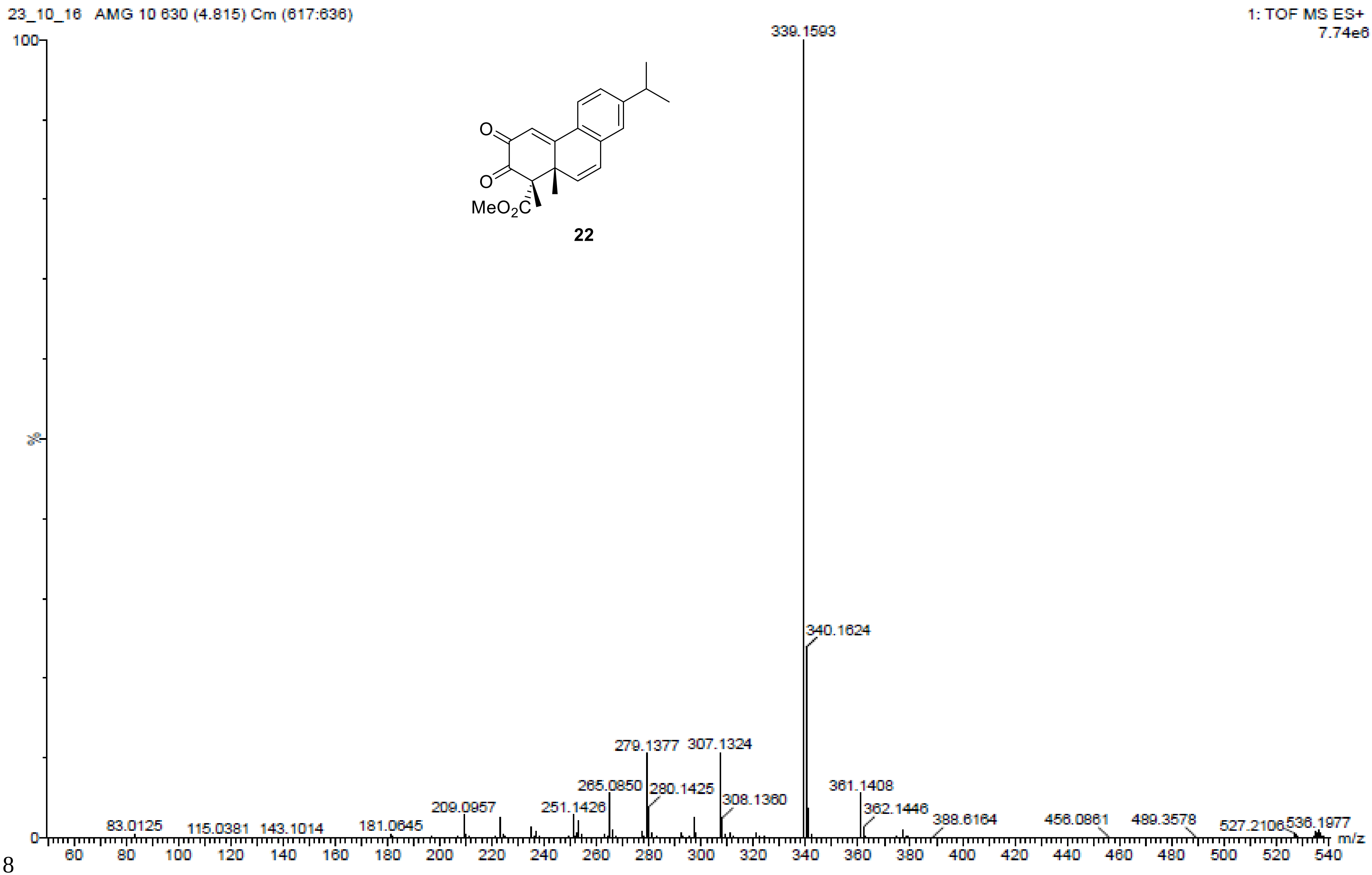

23_10_16 AMG 10 630 (4.815) Cm (617:636)
1: TOF MS ES+
7.74e6
100
%
0
339.1593
340.1624
279.1377
307.1324
265.0850
280.1425
308.1360
361.1408
362.1446
209.0957
251.1426
83.0125
115.0381
143.1014
181.0645
388.6164
456.0861
489.3578
527.2106
536.1977
MeO2C
O
O
22
m/z

8

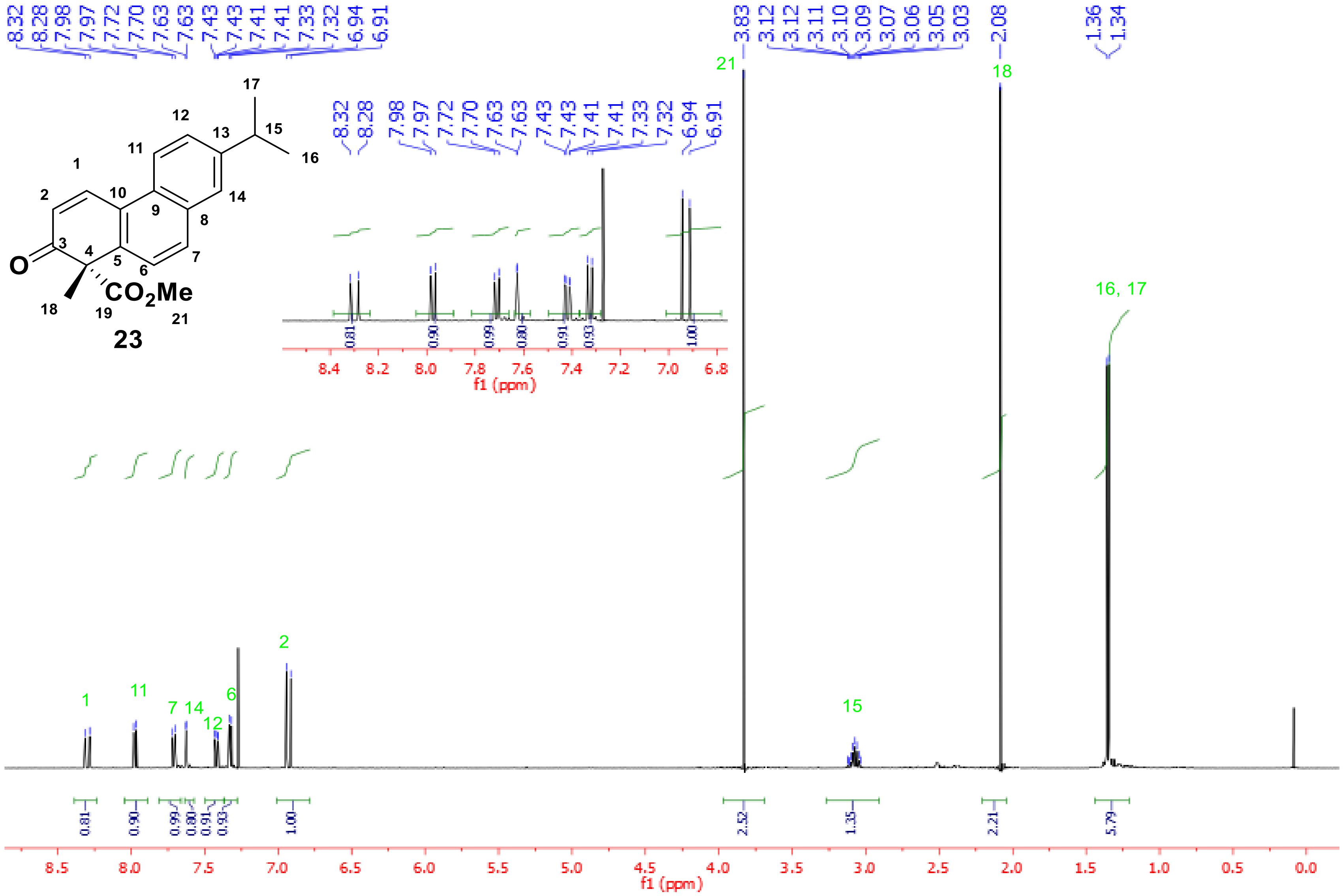

9

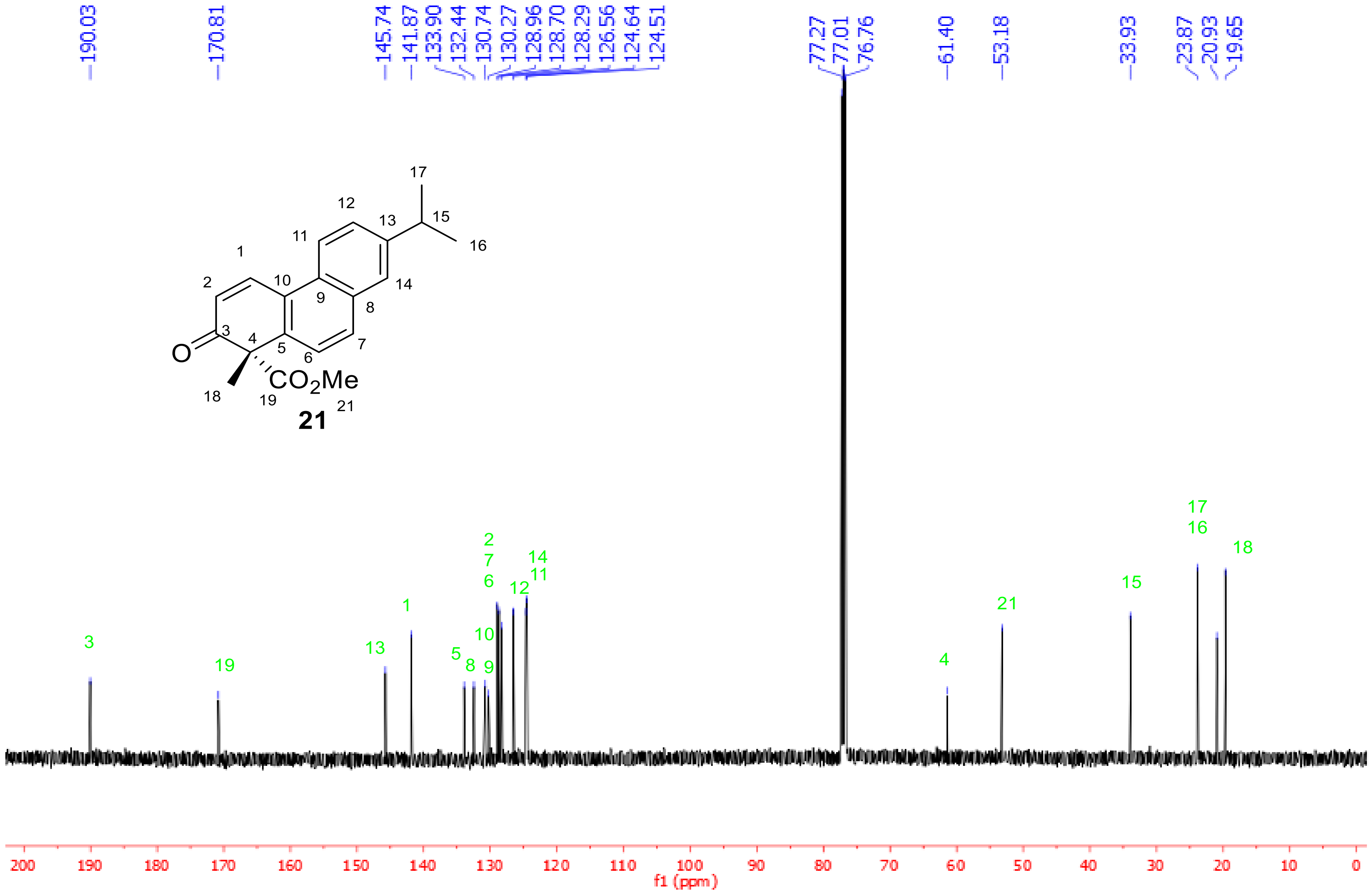

190.03
170.81
145.74
141.87
133.90
132.44
130.74
130.27
128.96
128.70
128.29
126.56
124.64
124.51
77.27
77.01
76.76
61.40
53.18
33.93
23.87
20.93
19.65
CO2Me
21
f1 (ppm)

10

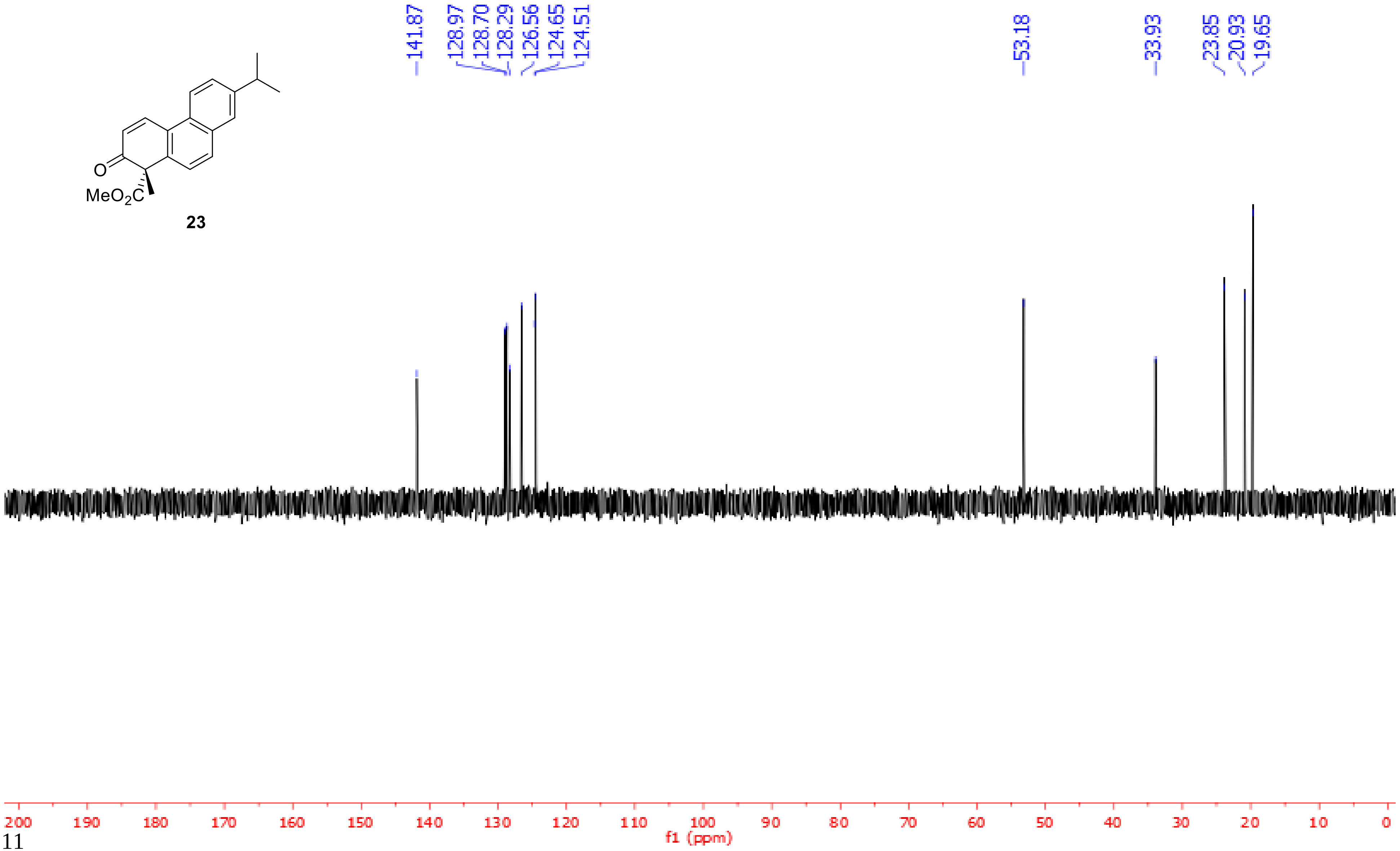
MeO2C
O
23
141.87
128.97
128.70
128.29
126.56
124.65
124.51
53.18
33.93
23.85
20.93
19.65
200 190 180 170 160 150 140 130 120 110 100 90 80 70 60 50 40 30 20 10 0
f1 (ppm)

11

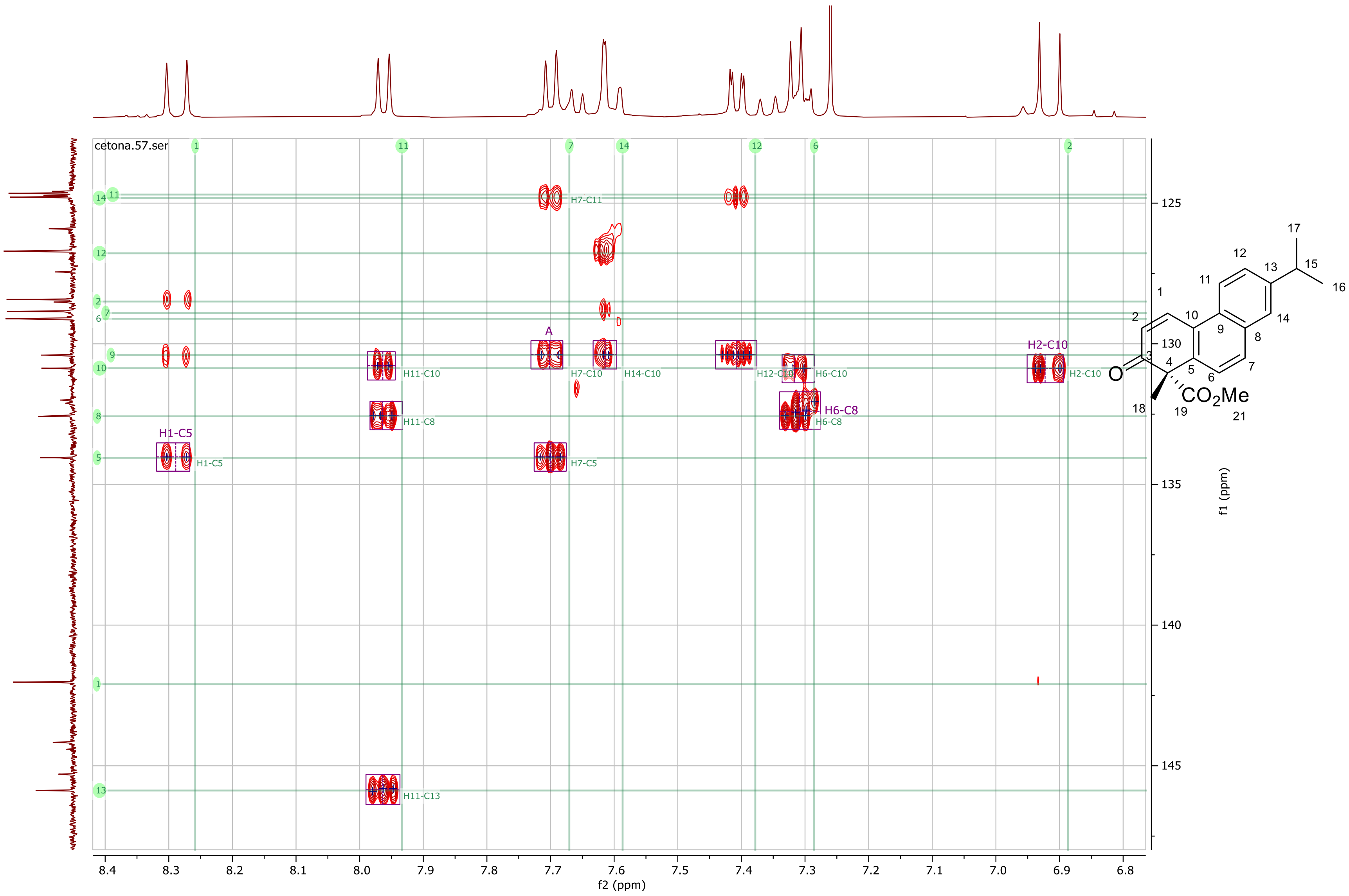
cetona.57.ser
H7-C11
H11-C10
H7-C10
H14-C10
H12-C10
H6-C10
H2-C10
H11-C8
H6-C8
H1-C5
H7-C5
H11-C13
f2 (ppm)
f1 (ppm)
CO2Me

12

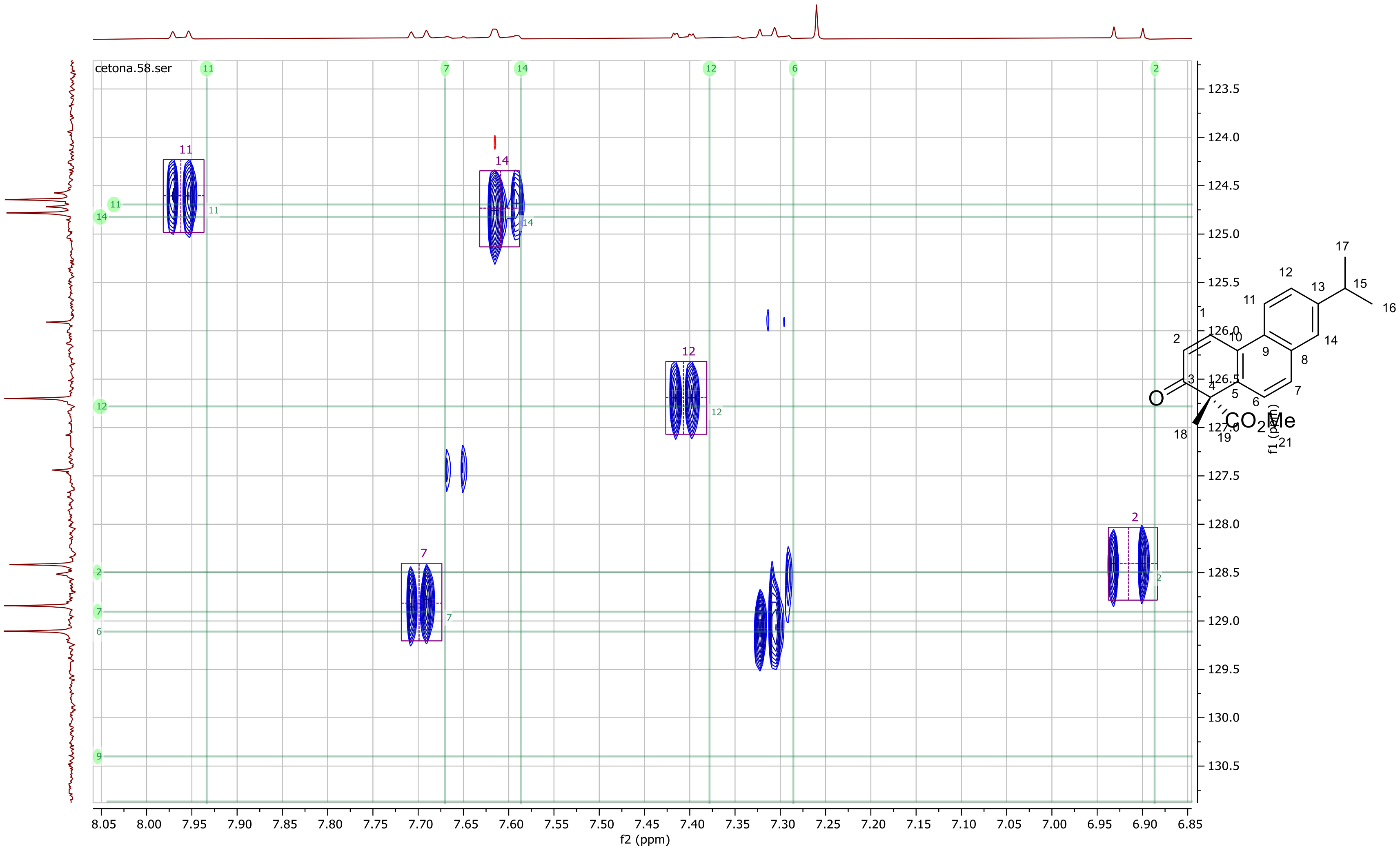

cetona.58.ser
f2 (ppm)
f1 (ppm)
CO2Me
O

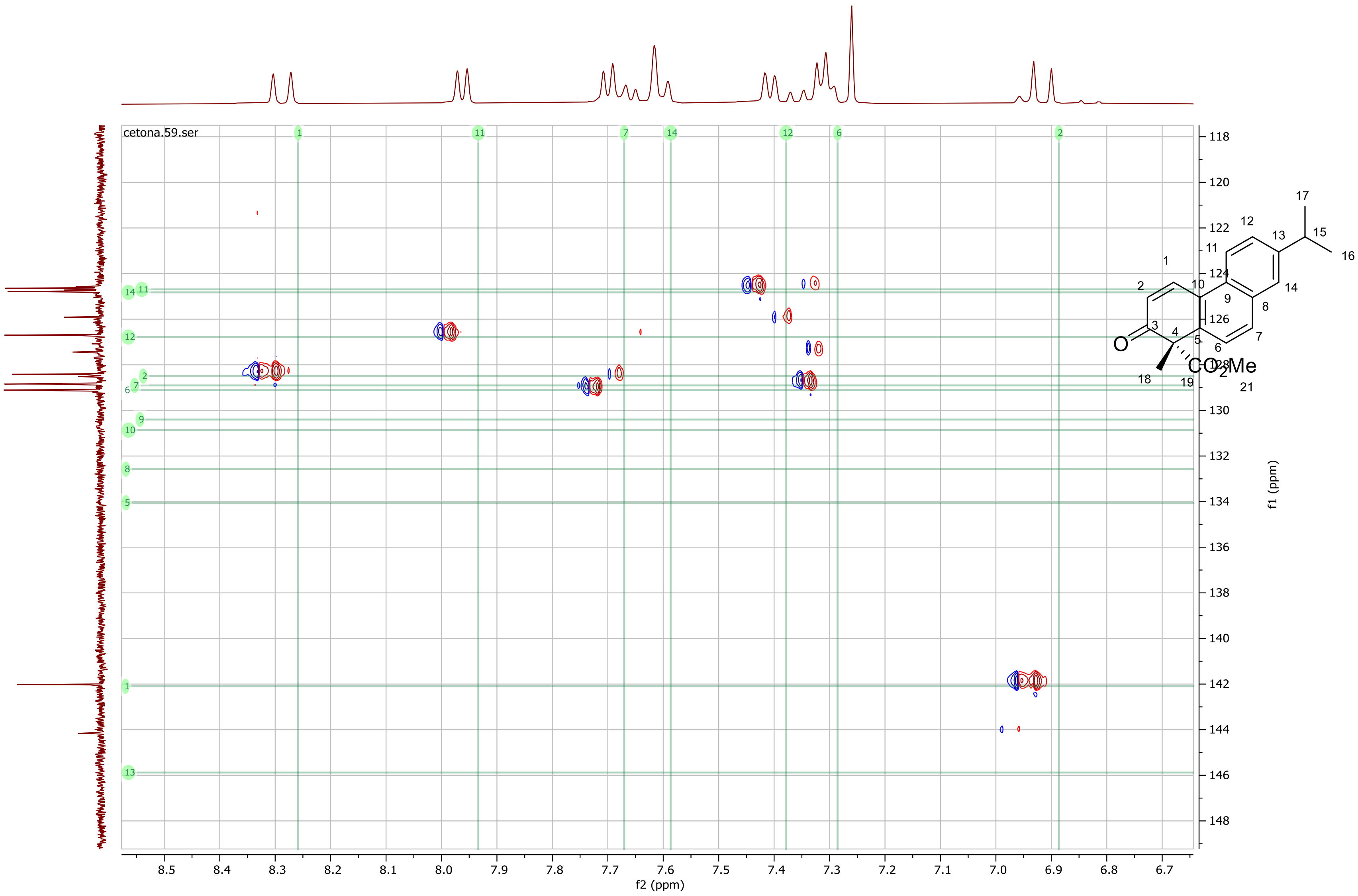

14

cetona.60.fid
PROTON CDCl3 {C:\Bruker\data} Ali 5

1D Selective Gradient TOCSY (with DIPSI and ZS)
freq: 8.319ppm

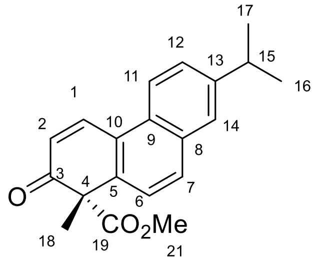

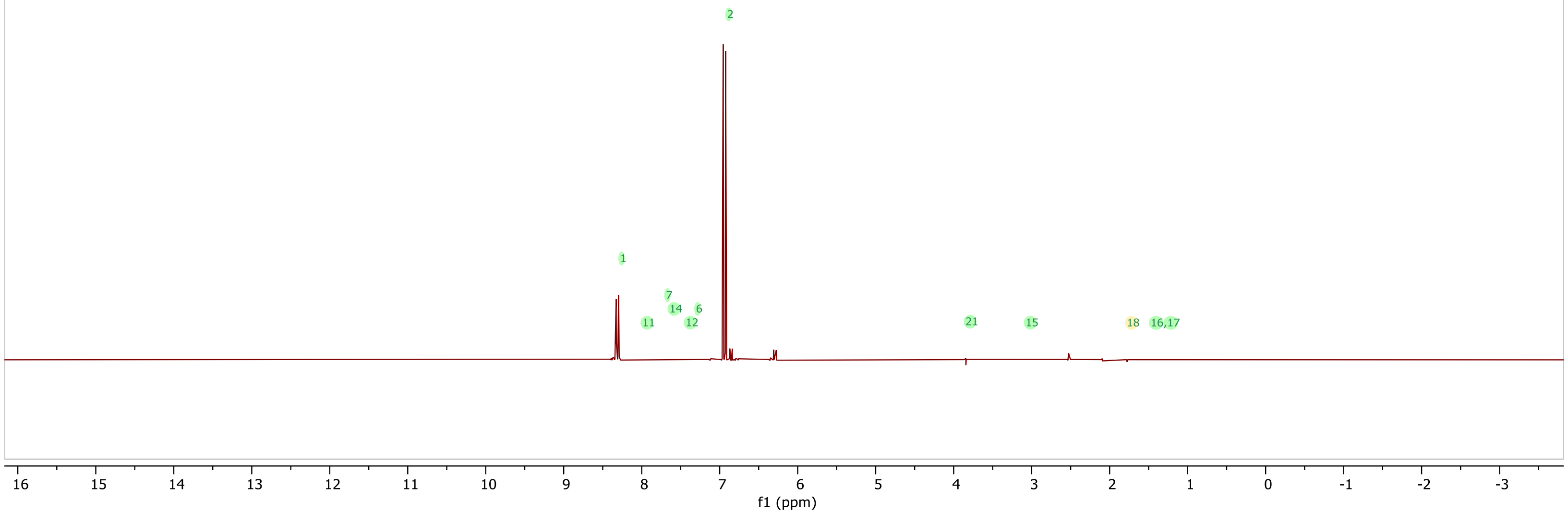

15

cetona.61.fid
PROTON CDCl3 {C:\Bruker\data} Ali 5

1D Selective Gradient TOCSY (with DIPSI and ZS)
freq: 7.991ppm

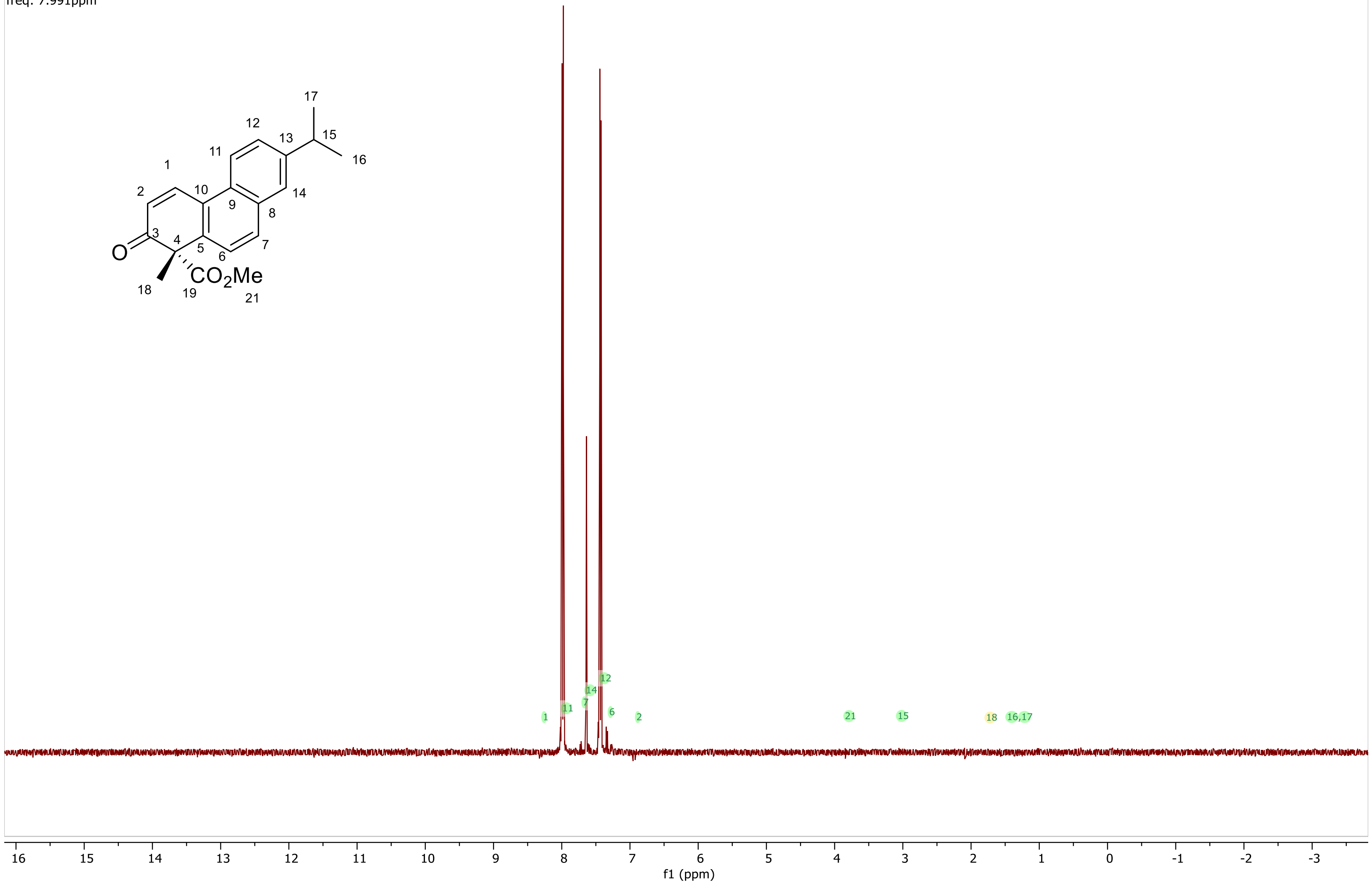

16

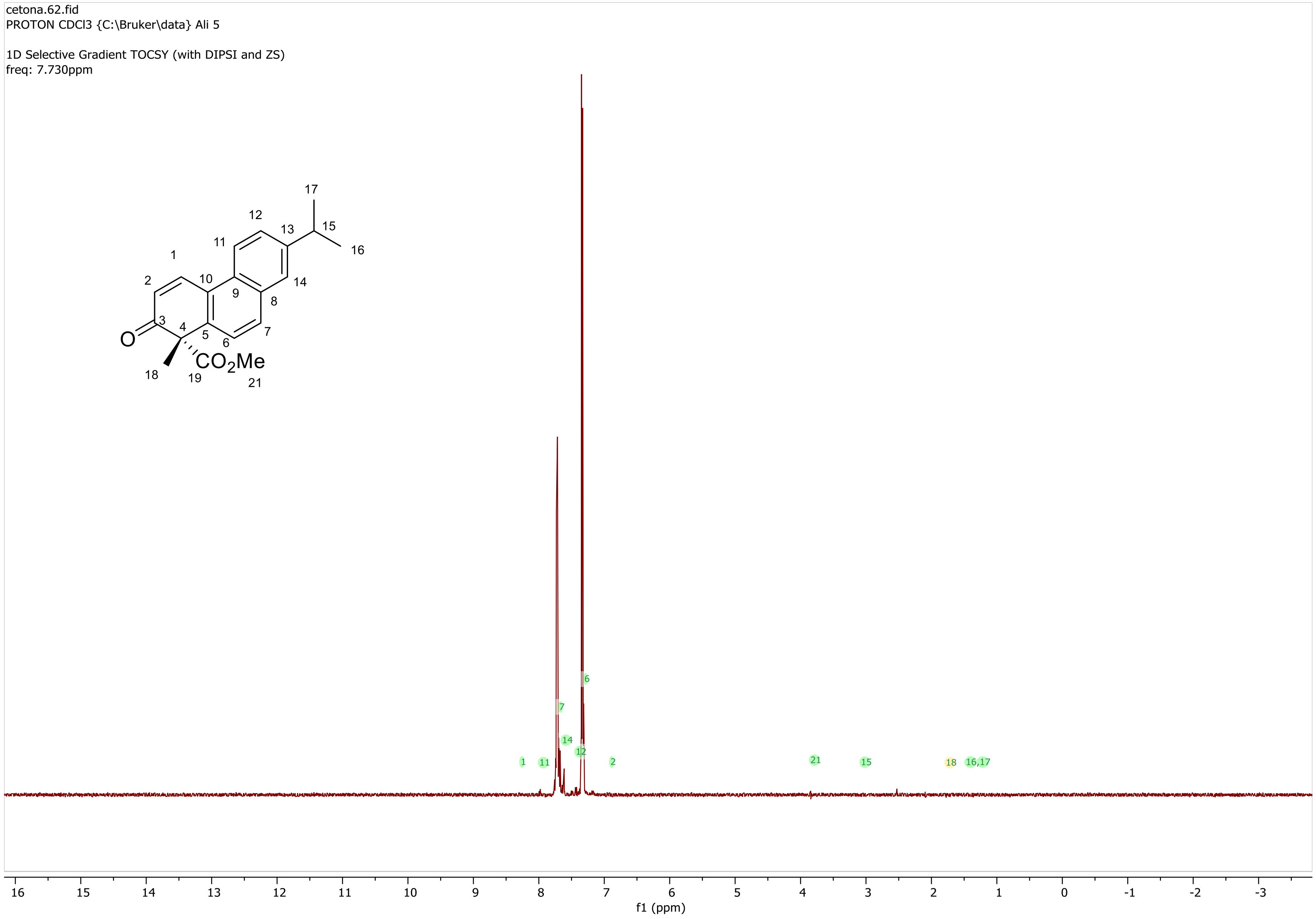
cetona.62.fid
PROTON CDCl3 {C:\Bruker\data} Ali 5
1D Selective Gradient TOCSY (with DIPSI and ZS)
freq: 7.730ppm
CO2Me
f1 (ppm)

17

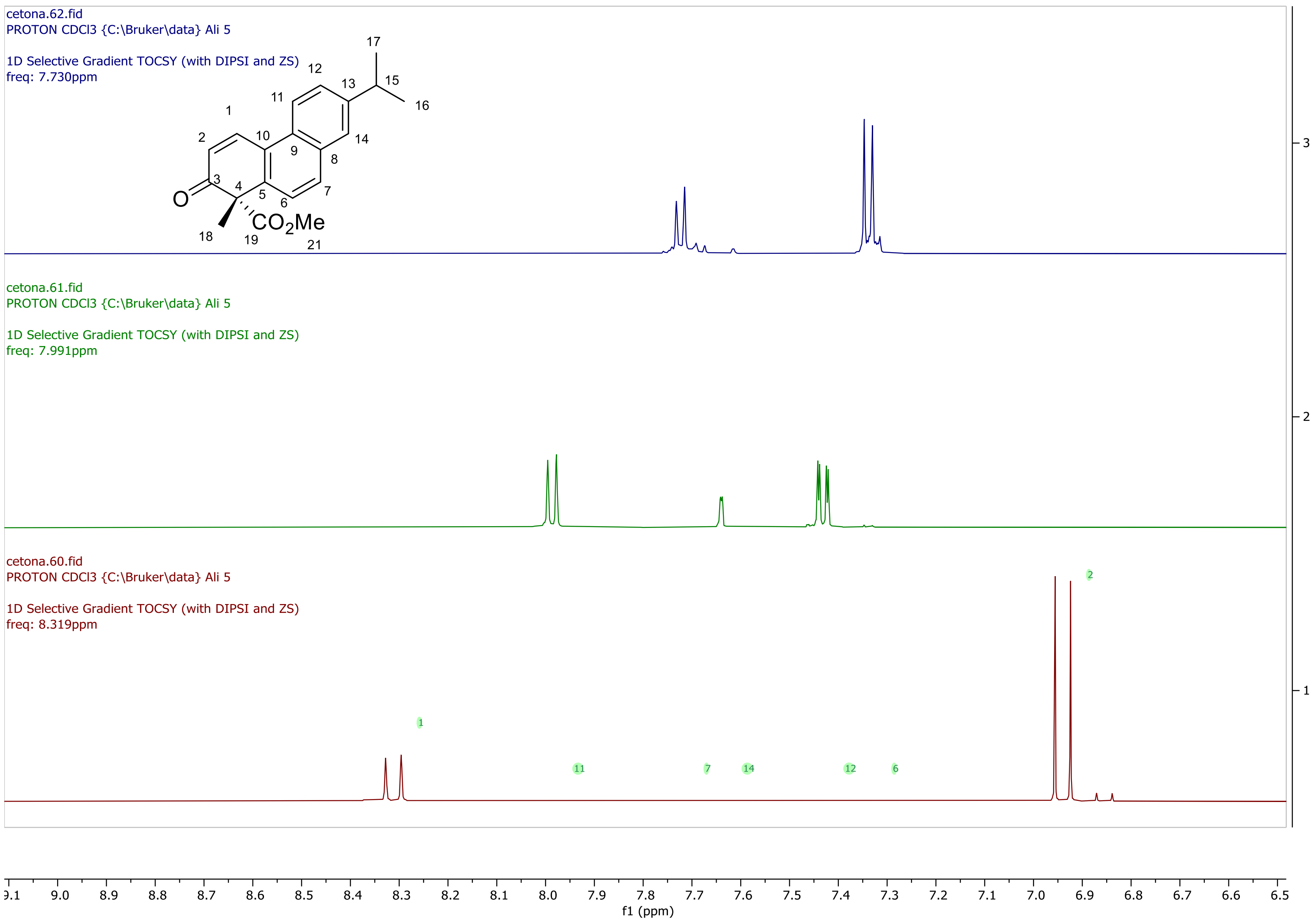

cetona.62.fid
PROTON CDCl3 {C:\Bruker\data} Ali 5
1D Selective Gradient TOCSY (with DIPSI and ZS)
freq: 7.730ppm
cetona.61.fid
PROTON CDCl3 {C:\Bruker\data} Ali 5
1D Selective Gradient TOCSY (with DIPSI and ZS)
freq: 7.991ppm
cetona.60.fid
PROTON CDCl3 {C:\Bruker\data} Ali 5
1D Selective Gradient TOCSY (with DIPSI and ZS)
freq: 8.319ppm
CO2Me
f1 (ppm)

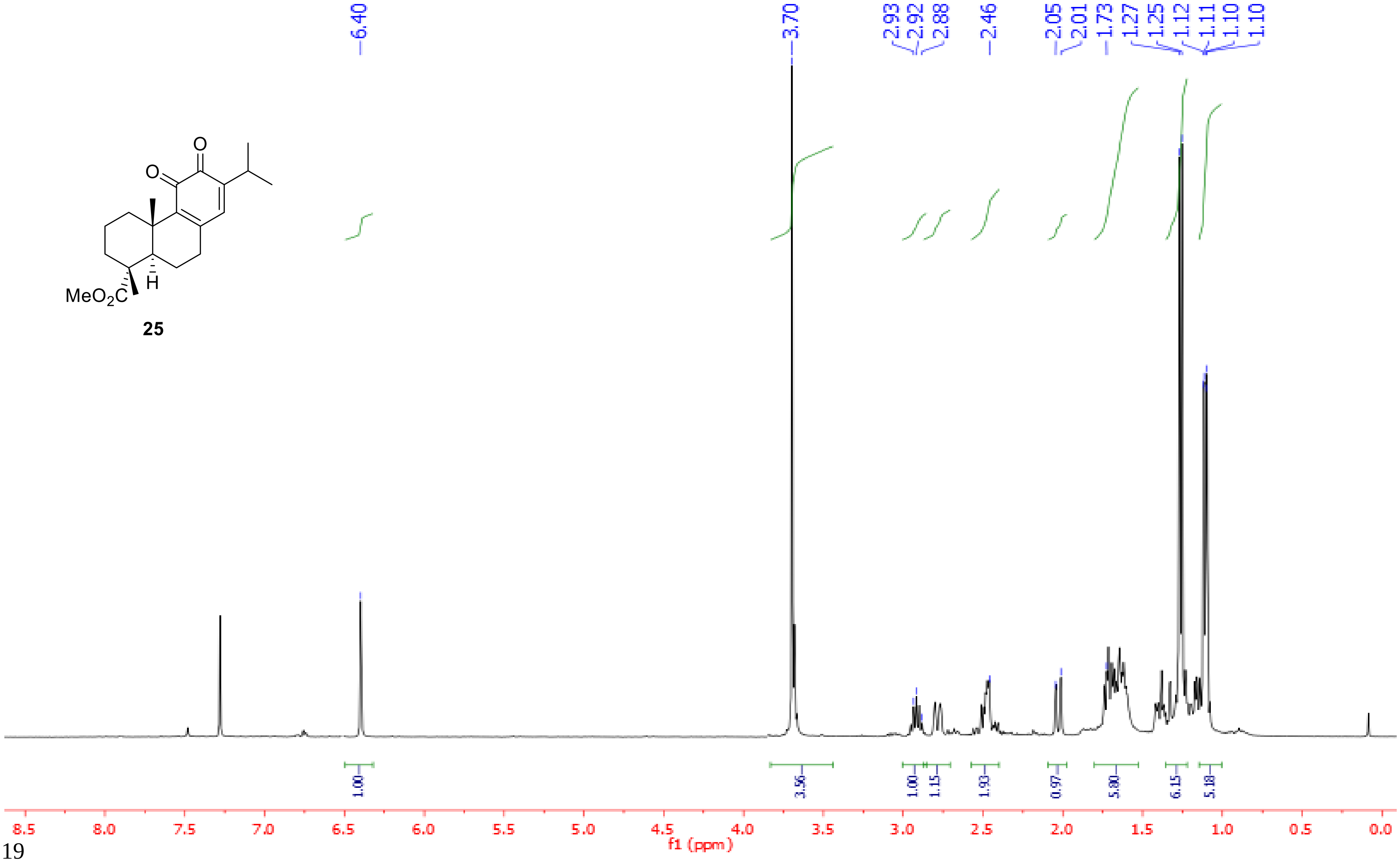
25
6.40
3.70
2.93
2.92
2.88
2.46
2.05
2.01
1.73
1.27
1.25
1.12
1.11
1.10
1.10
1.00
3.56
1.00
1.15
1.93
0.97
5.80
6.15
5.18
8.5
8.0
7.5
7.0
6.5
6.0
5.5
5.0
4.5
4.0
3.5
3.0
2.5
2.0
1.5
1.0
0.5
0.0
f1 (ppm)

19

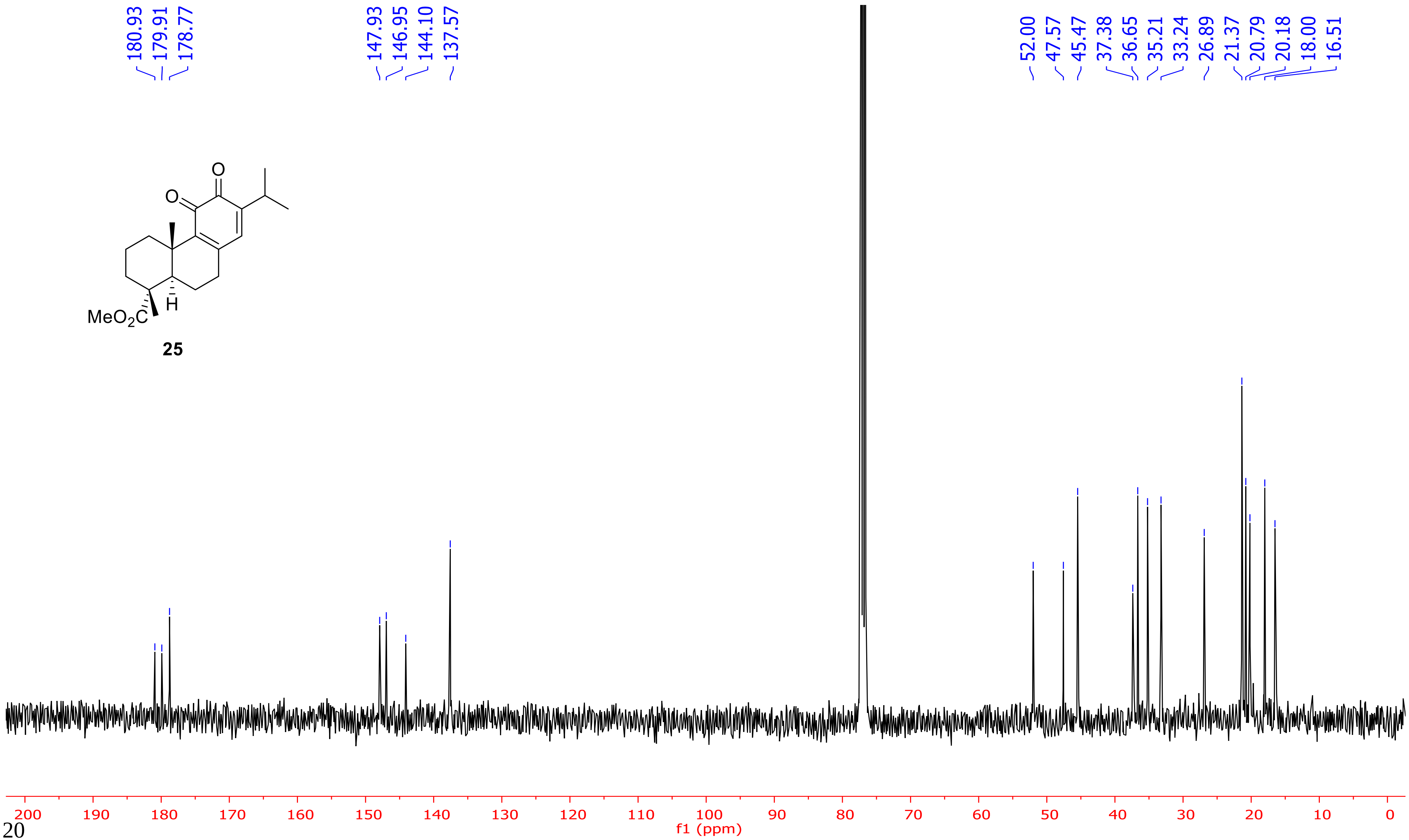
180.93
179.91
178.77
147.93
146.95
144.10
137.57
52.00
47.57
45.47
37.38
36.65
35.21
33.24
26.89
21.37
20.79
20.18
18.00
16.51
O
O
MeO_2C
H
25
200 190 180 170 160 150 140 130 120 110 100 90 80 70 60 50 40 30 20 10 0
f1 (ppm)

20

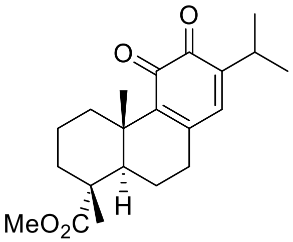

25

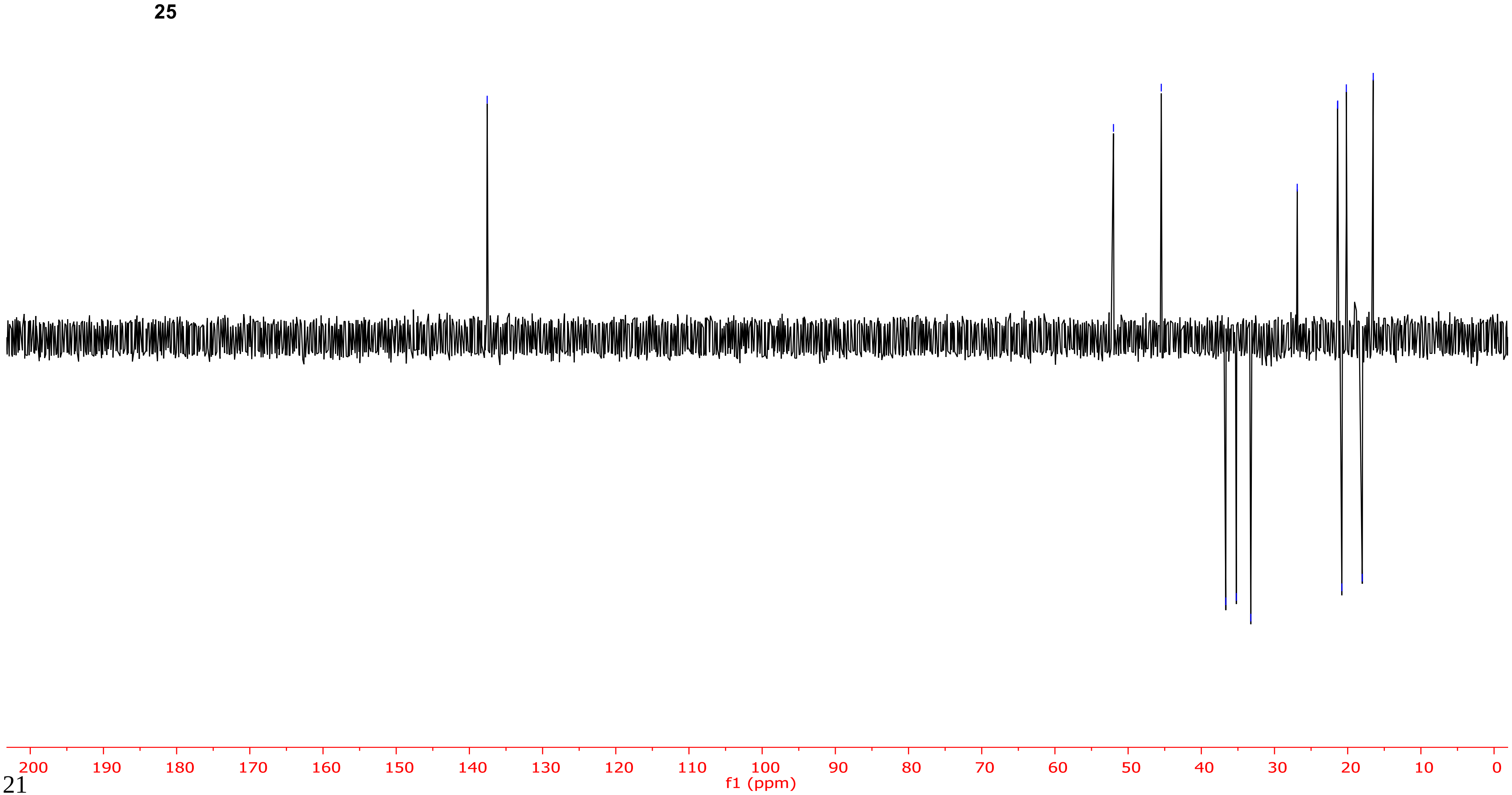

137.57
52.00
45.47
36.66
35.21
33.24
26.89
21.38
21.36
20.79
20.19
18.01
16.51
200
190
180
170
160
150
140
130
120
110
100
90
80
70
60
50
40
30
20
10
0
f1 (ppm)

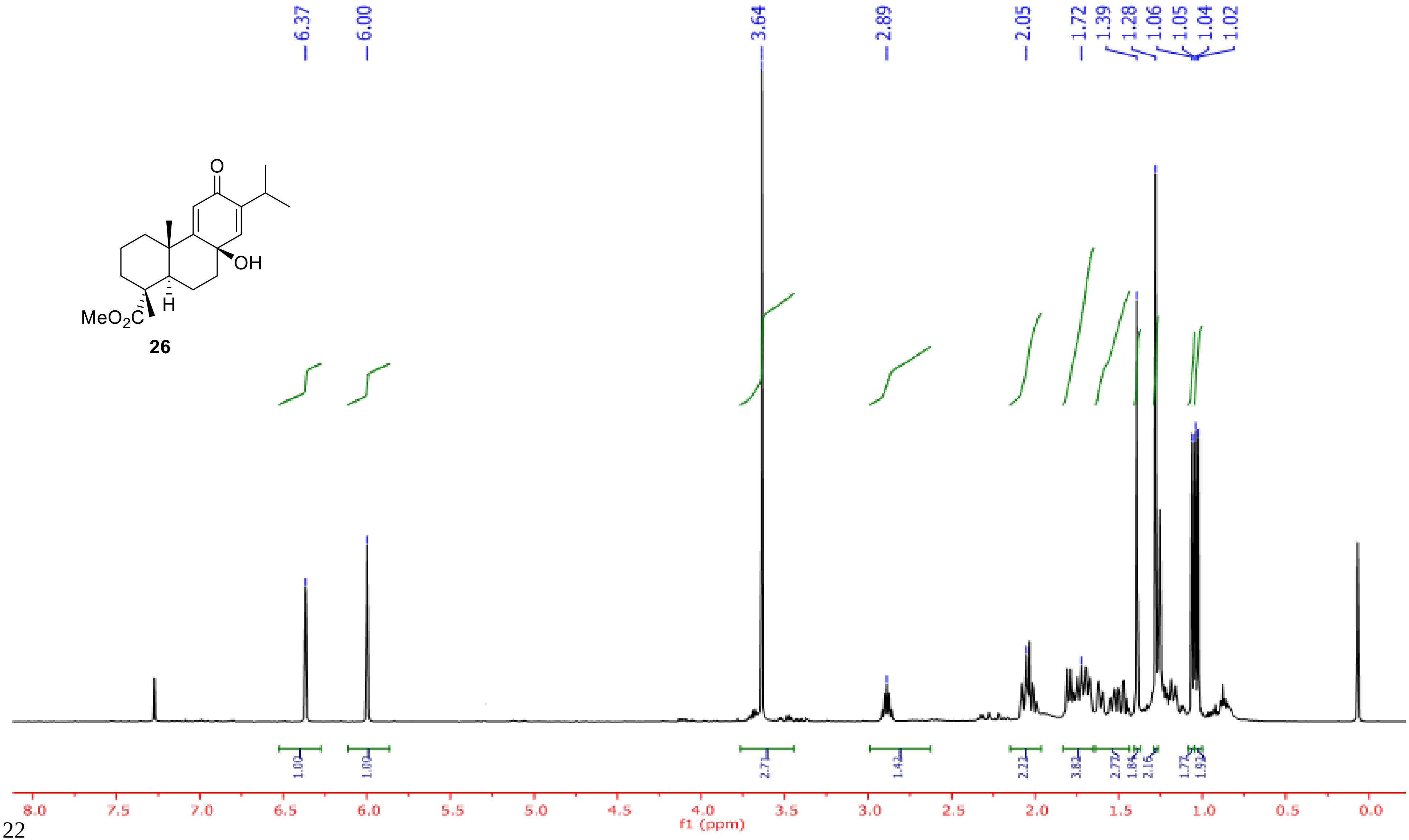
6.37
6.00
3.64
2.89
2.05
1.72
1.39
1.28
1.06
1.05
1.04
1.02
O
OH
H
MeO2C
26
1.00
1.00
2.71
1.42
2.21
3.83
2.72
1.84
2.16
1.77
1.92
8.0 7.5 7.0 6.5 6.0 5.5 5.0 4.5 4.0 3.5 3.0 2.5 2.0 1.5 1.0 0.5 0.0
f1 (ppm)

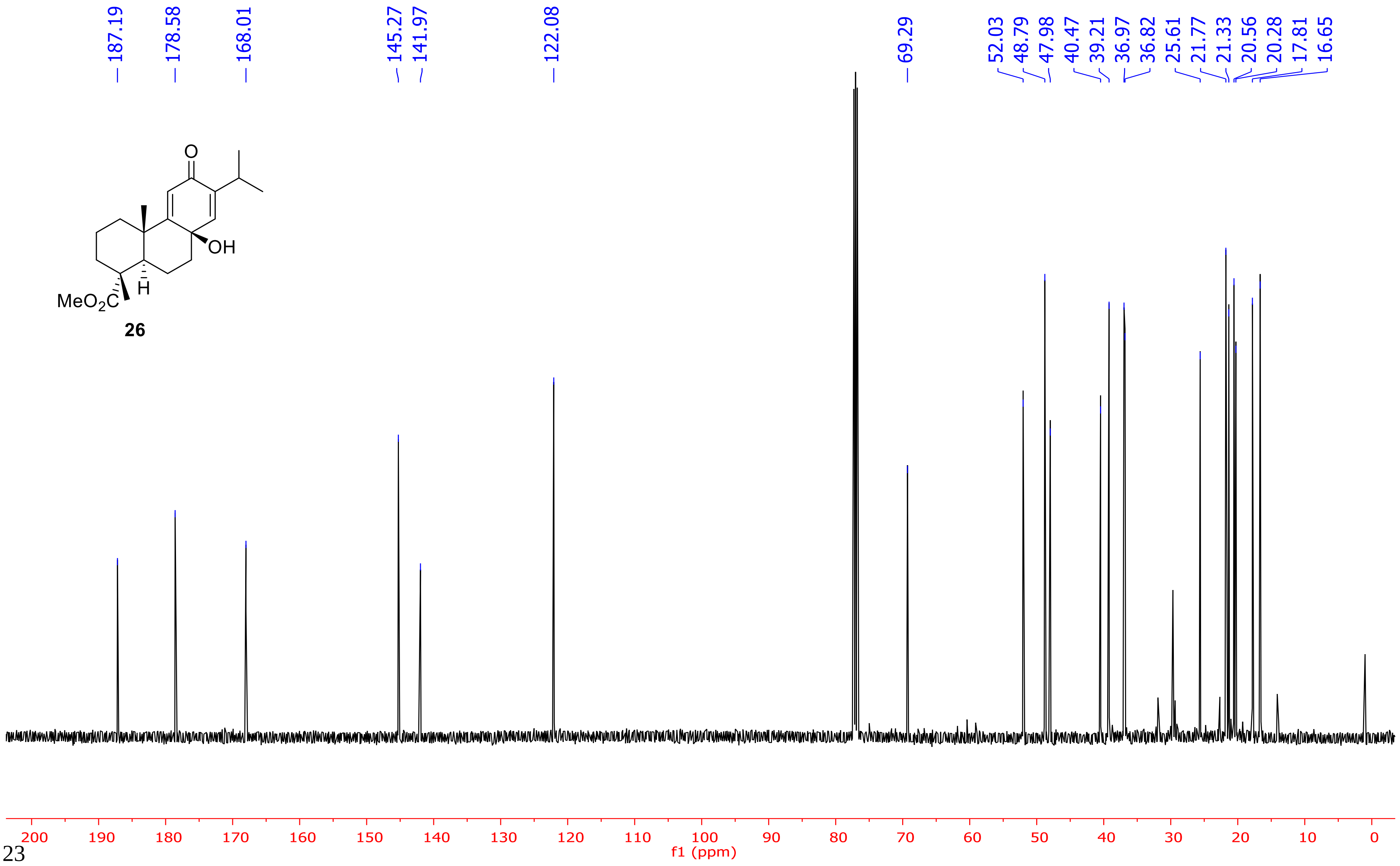

187.19
178.58
168.01
145.27
141.97
122.08
69.29
52.03
48.79
47.98
40.47
39.21
36.97
36.82
25.61
21.77
21.33
20.56
20.28
17.81
16.65
O
OH
H
MeO2C
26
200 190 180 170 160 150 140 130 120 110 100 90 80 70 60 50 40 30 20 10 0
f1 (ppm)

23

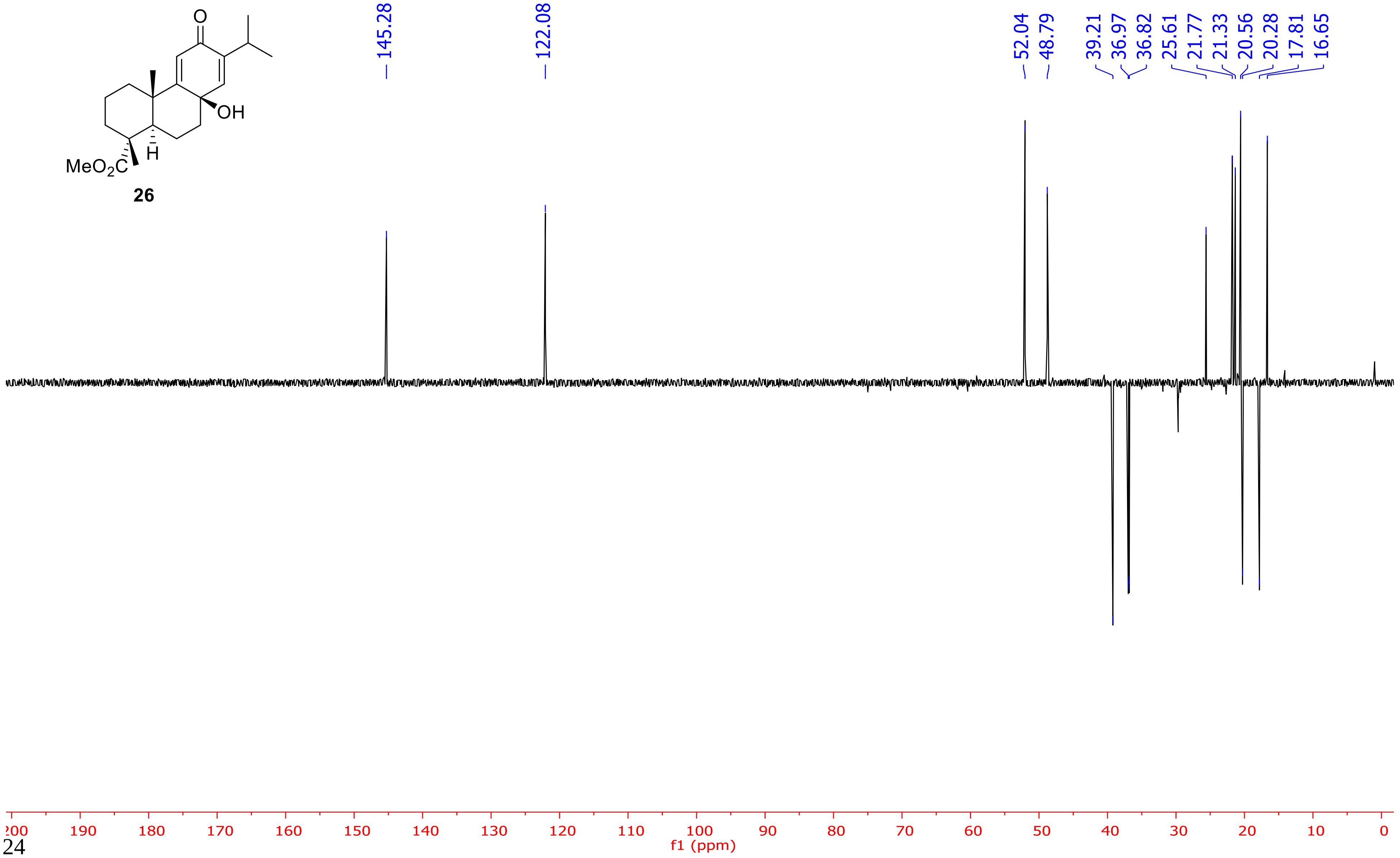
O
OH
H
MeO2C
26
145.28
122.08
52.04
48.79
39.21
36.97
36.82
25.61
21.77
21.33
20.56
20.28
17.81
16.65
200
190
180
170
160
150
140
130
120
110
100
90
80
70
60
50
40
30
20
10
0
f1 (ppm)
24

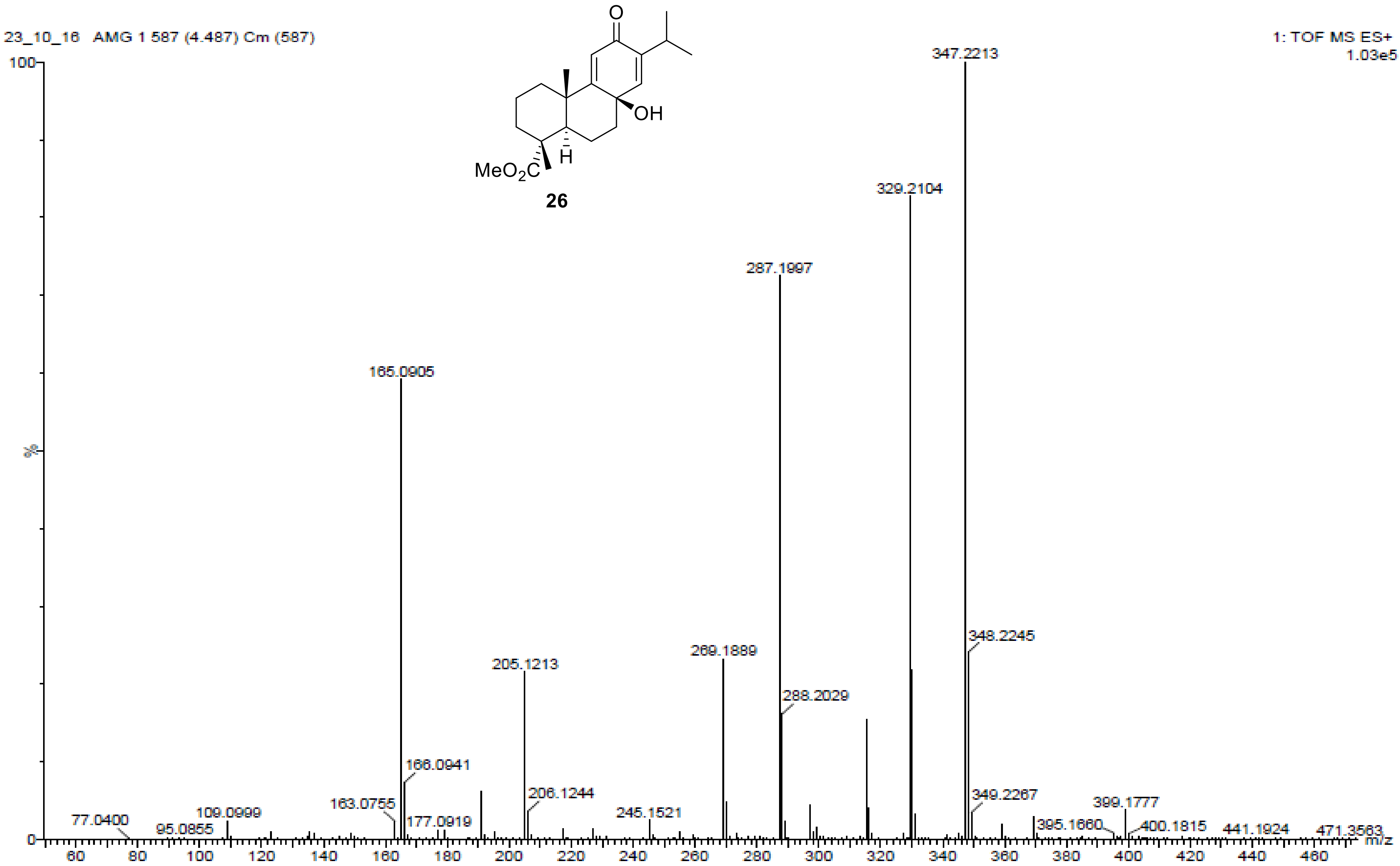
O
OH
H
MeO2C
26
23_10_16 AMG 1 587 (4.487) Cm (587)
1: TOF MS ES+
1.03e5
347.2213
329.2104
287.1997
165.0905
348.2245
269.1889
205.1213
288.2029
166.0941
206.1244
349.2267
399.1777
163.0755
245.1521
109.0999
77.0400
177.0919
395.1660
400.1815
95.0855
441.1924
471.3563
%
m/z

25

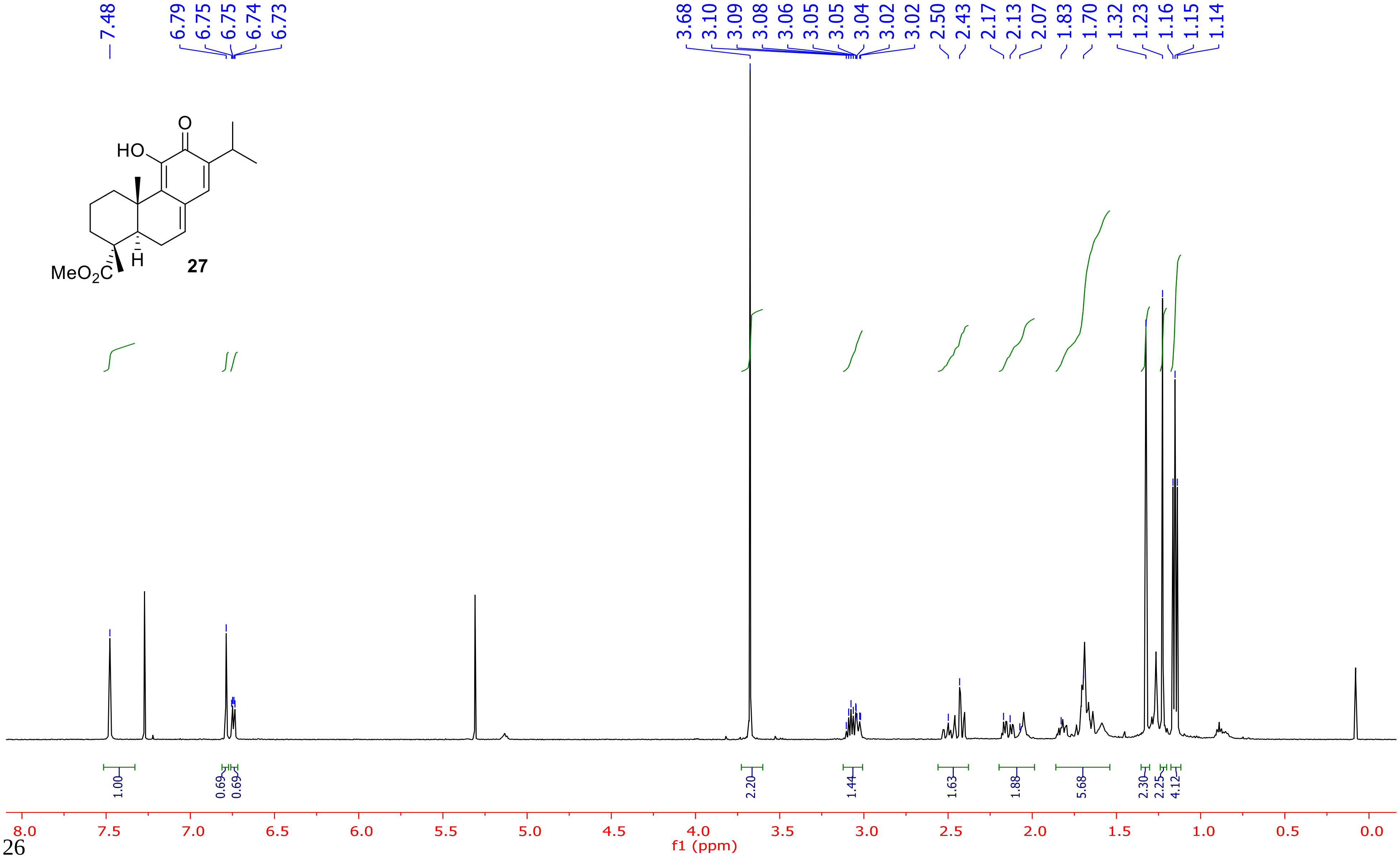
7.48
6.79 6.75 6.75 6.74 6.73
3.68 3.10 3.09 3.08 3.06 3.05 3.05 3.04 3.02 3.02 2.50 2.43 2.17 2.13 2.07 1.83 1.70 1.32 1.23 1.16 1.15 1.14
HO
O
MeO2C
H
27
1.00
0.69
0.69
2.20
1.44
1.63
1.88
5.68
2.30
2.25
4.12
8.0 7.5 7.0 6.5 6.0 5.5 5.0 4.5 4.0 3.5 3.0 2.5 2.0 1.5 1.0 0.5 0.0
f1 (ppm)

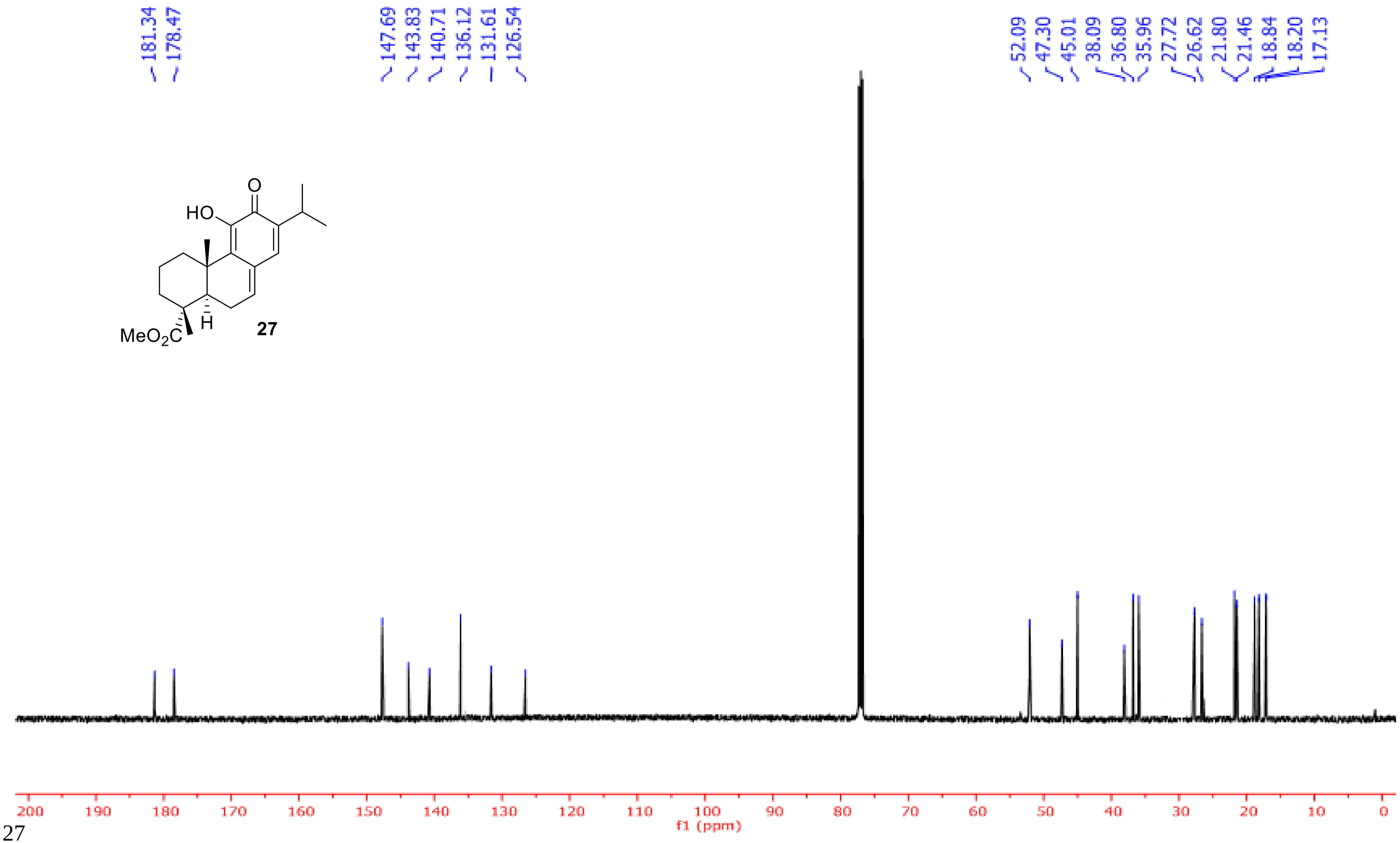
181.34
178.47
147.69
143.83
140.71
136.12
131.61
126.54
52.09
47.30
45.01
38.09
36.80
35.96
27.72
26.62
21.80
21.46
18.84
18.20
17.13
HO
O
MeO2C
H
27
200
190
180
170
160
150
140
130
120
110
100
90
80
70
60
50
40
30
20
10
0
f1 (ppm)

27

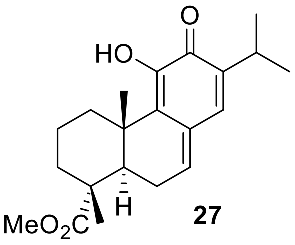

27

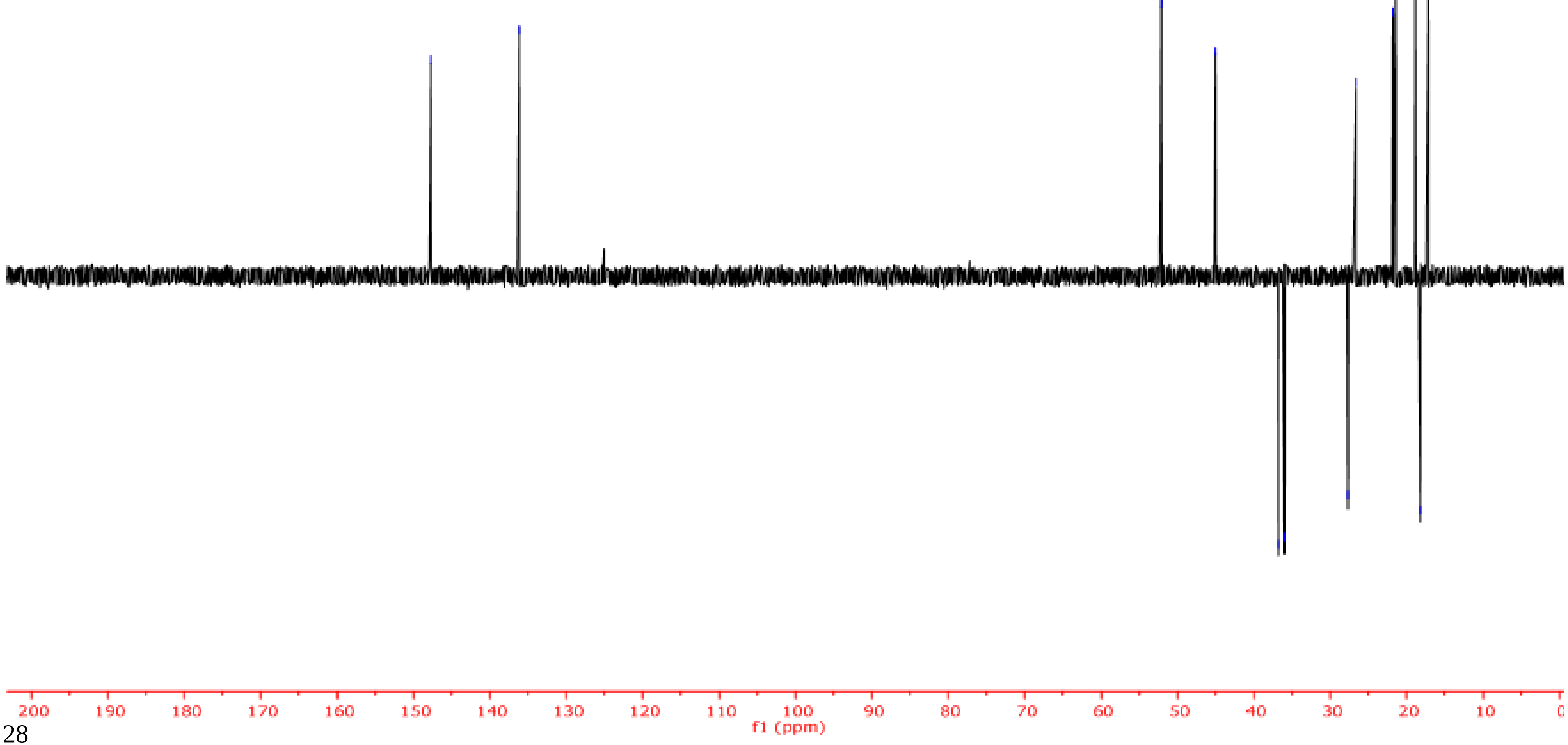

147.70
136.12
52.09
45.01
36.80
35.97
27.72
26.62
21.80
21.46
18.84
18.20
17.13
200 190 180 170 160 150 140 130 120 110 100 90 80 70 60 50 40 30 20 10 0
f1 (ppm)

28

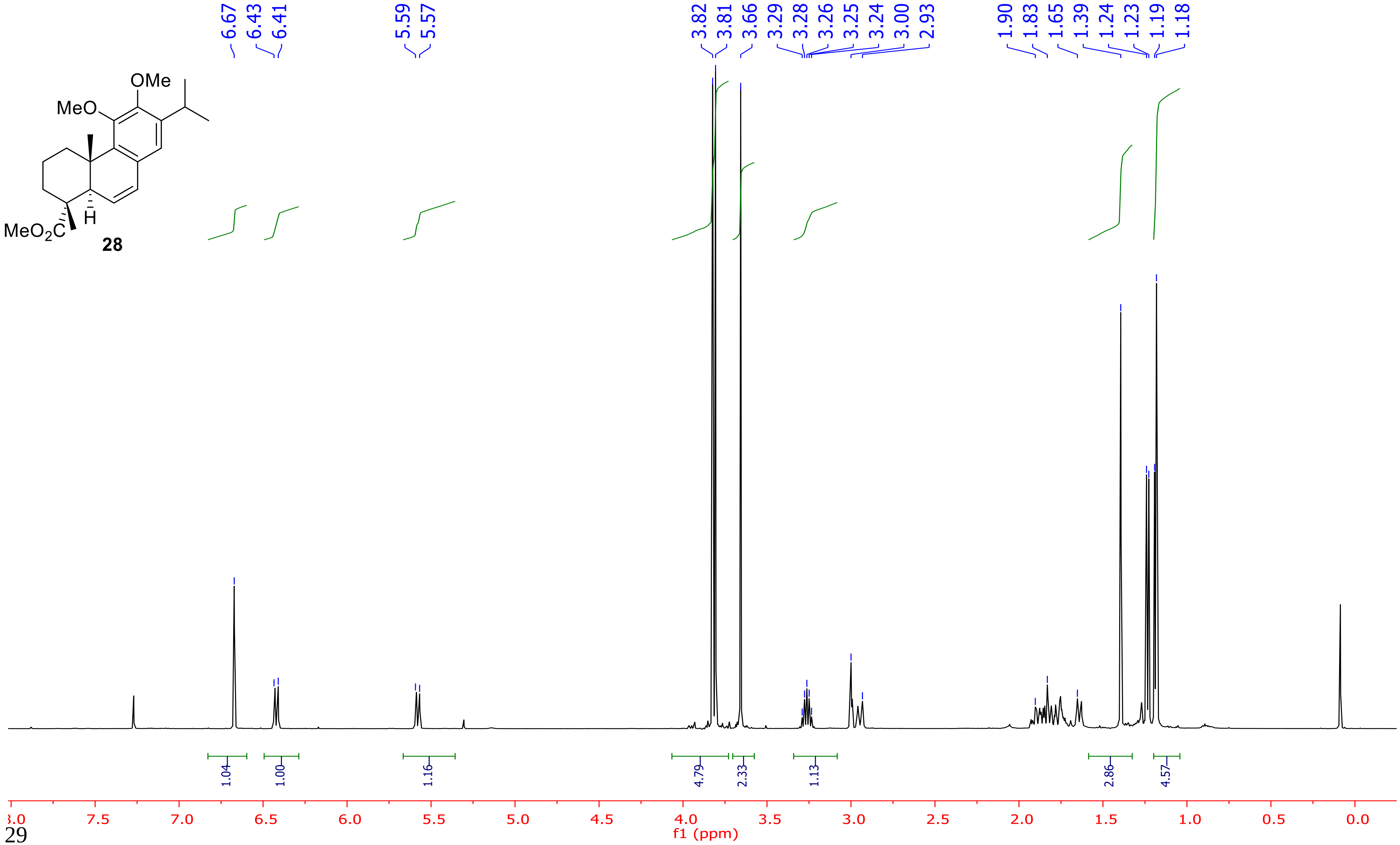
OMe
MeO
H
MeO2C
28
6.67
6.43
6.41
5.59
5.57
3.82
3.81
3.66
3.29
3.28
3.26
3.25
3.24
3.00
2.93
1.90
1.83
1.65
1.39
1.24
1.23
1.19
1.18
1.04
1.00
1.16
4.79
2.33
1.13
2.86
4.57
7.5
7.0
6.5
6.0
5.5
5.0
4.5
4.0
3.5
3.0
2.5
2.0
1.5
1.0
0.5
0.0
f1 (ppm)

29

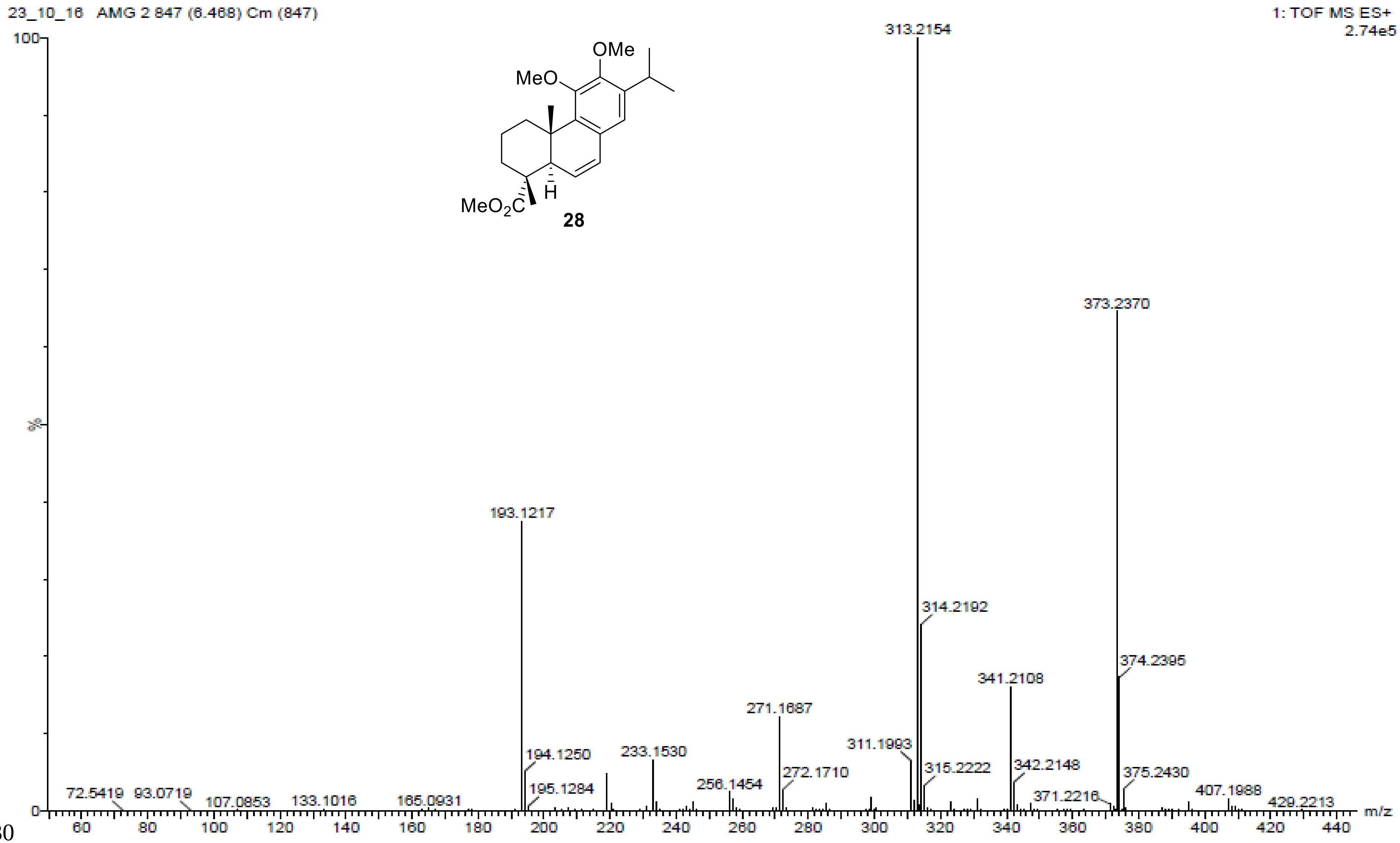
23_10_16 AMG 2 847 (6.468) Cm (847)
1: TOF MS ES+
2.74e5
OMe
MeO
MeO2C
H
28
313.2154
373.2370
193.1217
314.2192
374.2395
341.2108
271.1687
311.1993
233.1530
194.1250
315.2222
342.2148
272.1710
375.2430
256.1454
195.1284
407.1988
371.2216
429.2213
72.5419
93.0719
107.0853
133.1016
165.0931
100
%
0
m/z

30

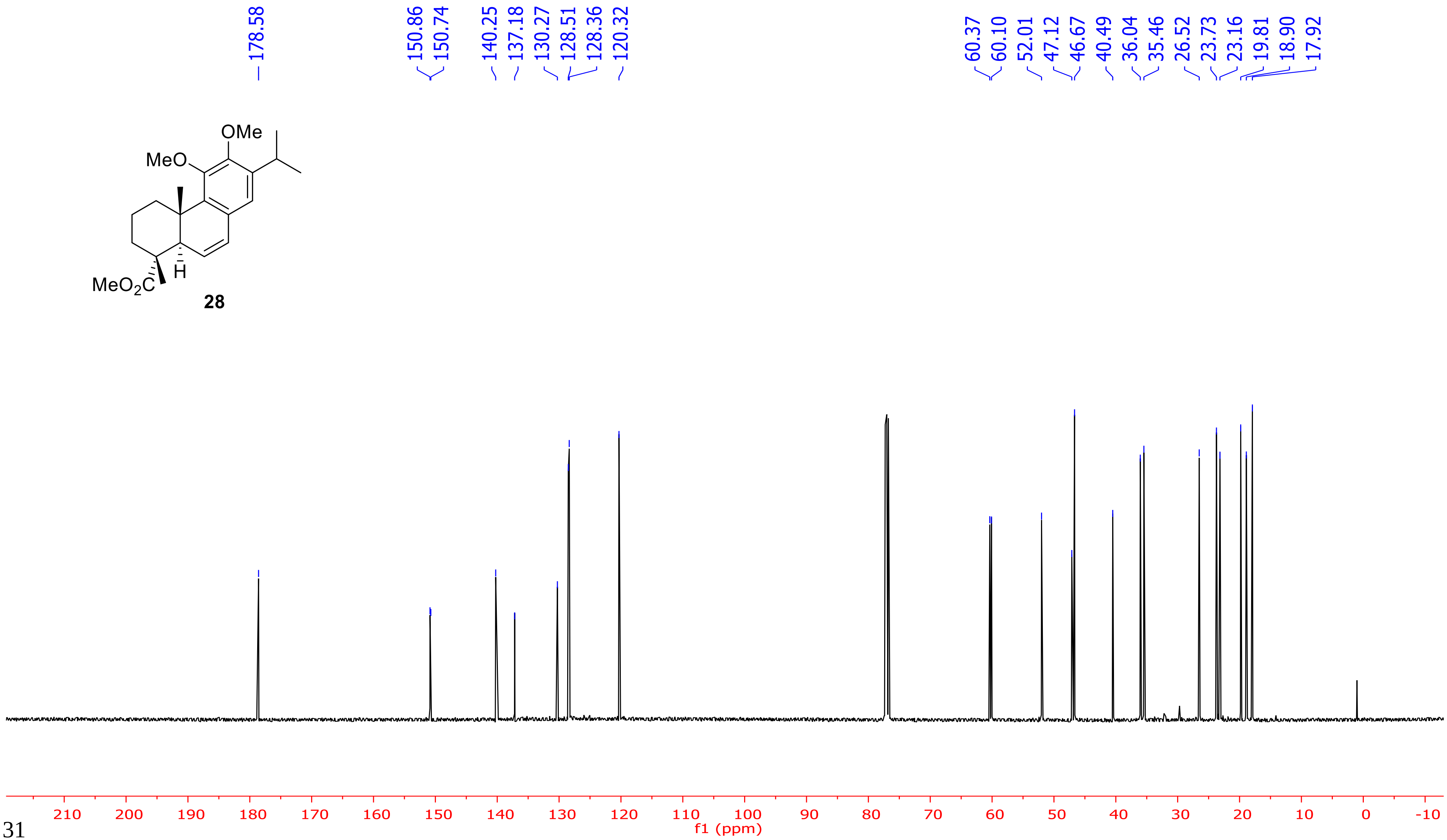
178.58
150.86
150.74
140.25
137.18
130.27
128.51
128.36
120.32
60.37
60.10
52.01
47.12
46.67
40.49
36.04
35.46
26.52
23.73
23.16
19.81
18.90
17.92
OMe
MeO
H
MeO_2C
28
210 200 190 180 170 160 150 140 130 120 110 100 90 80 70 60 50 40 30 20 10 0 -10
f1 (ppm)

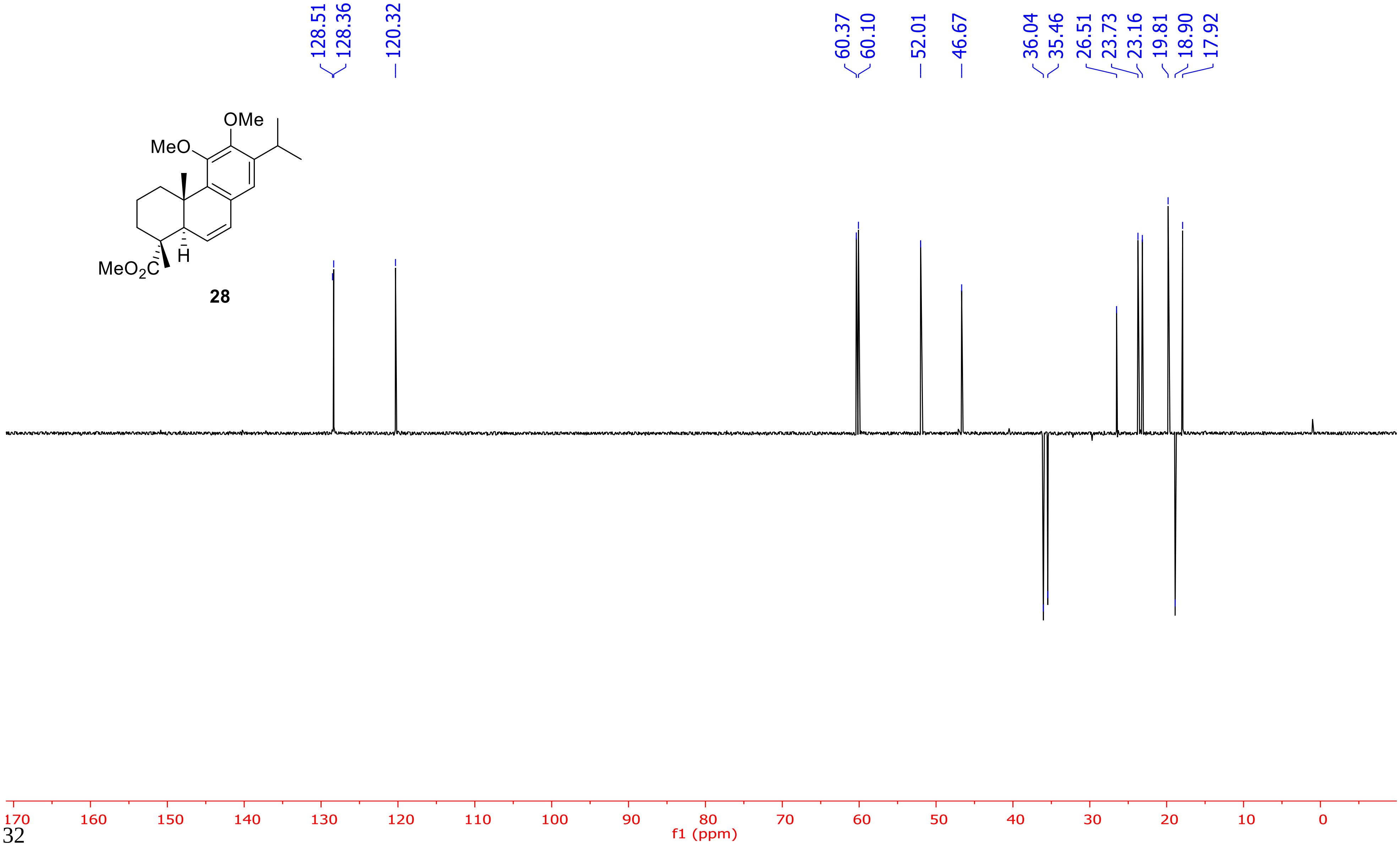
OMe
MeO
H
MeO2C
28
128.51
128.36
120.32
60.37
60.10
52.01
46.67
36.04
35.46
26.51
23.73
23.16
19.81
18.90
17.92
170
160
150
140
130
120
110
100
90
80
70
60
50
40
30
20
10
0
f1 (ppm)

32

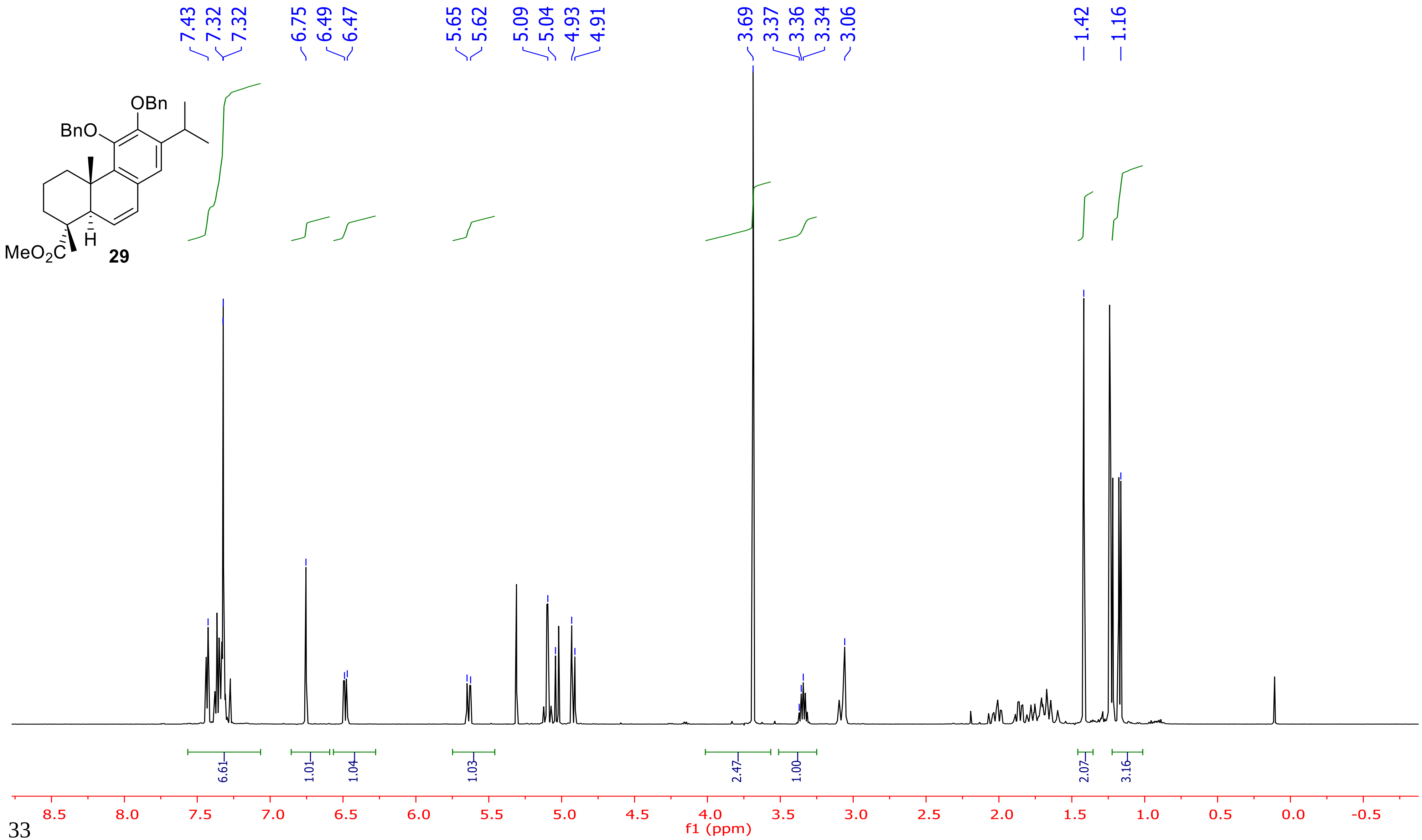
7.43
7.32
7.32
6.75
6.49
6.47
5.65
5.62
5.09
5.04
4.93
4.91
3.69
3.37
3.36
3.34
3.06
1.42
1.16
OBn
BnO
H
MeO_2C
29
6.61
1.01
1.04
1.03
2.47
1.00
2.07
3.16
8.5
8.0
7.5
7.0
6.5
6.0
5.5
5.0
4.5
4.0
3.5
3.0
2.5
2.0
1.5
1.0
0.5
0.0
-0.5
f1 (ppm)

33

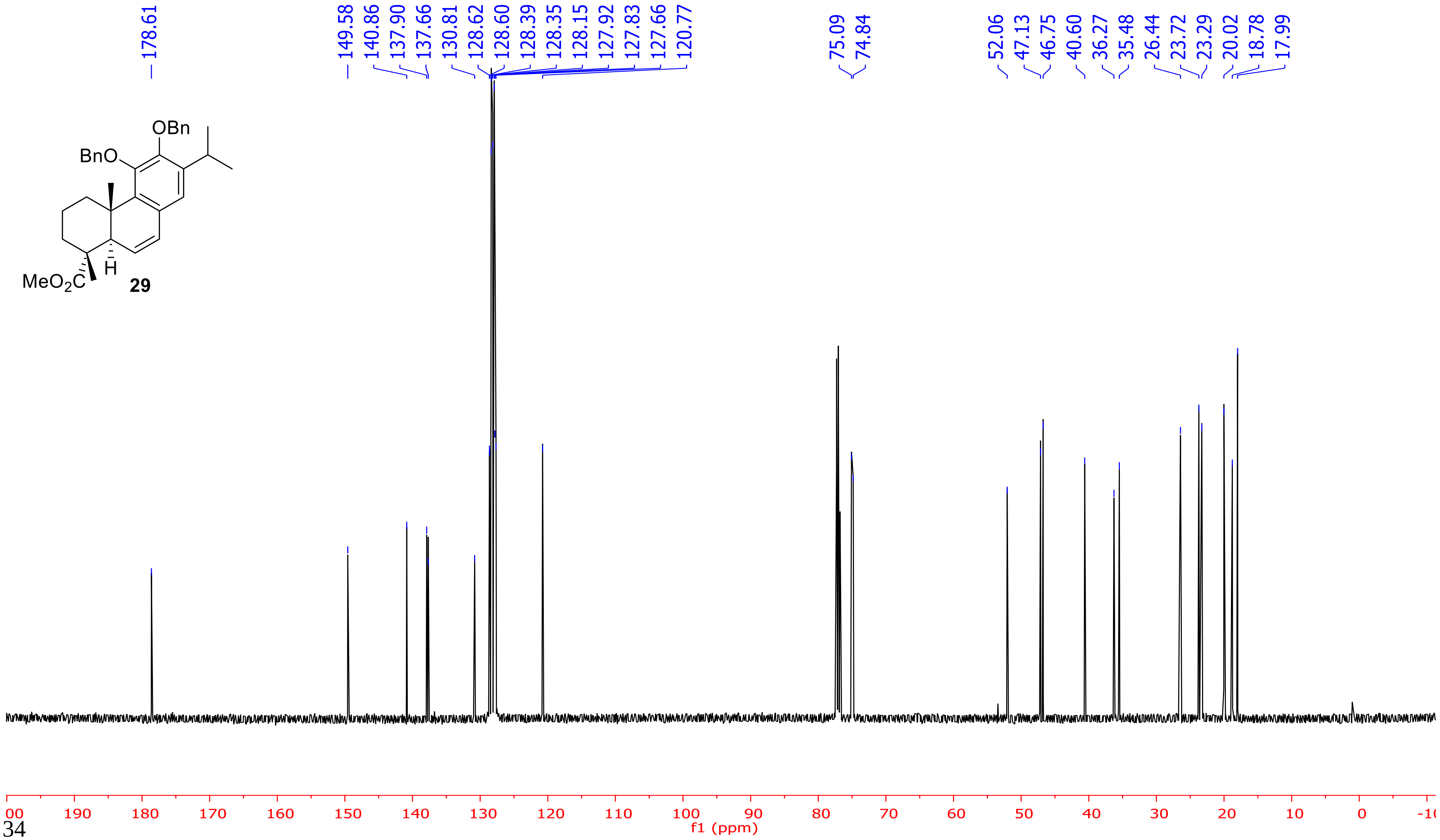
178.61
149.58
140.86
137.90
137.66
130.81
128.62
128.60
128.39
128.35
128.15
127.92
127.83
127.66
120.77
75.09
74.84
52.06
47.13
46.75
40.60
36.27
35.48
26.44
23.72
23.29
20.02
18.78
17.99
OBn
BnO
H
MeO2C
29
00 190 180 170 160 150 140 130 120 110 100 90 80 70 60 50 40 30 20 10 0
f1 (ppm)

34

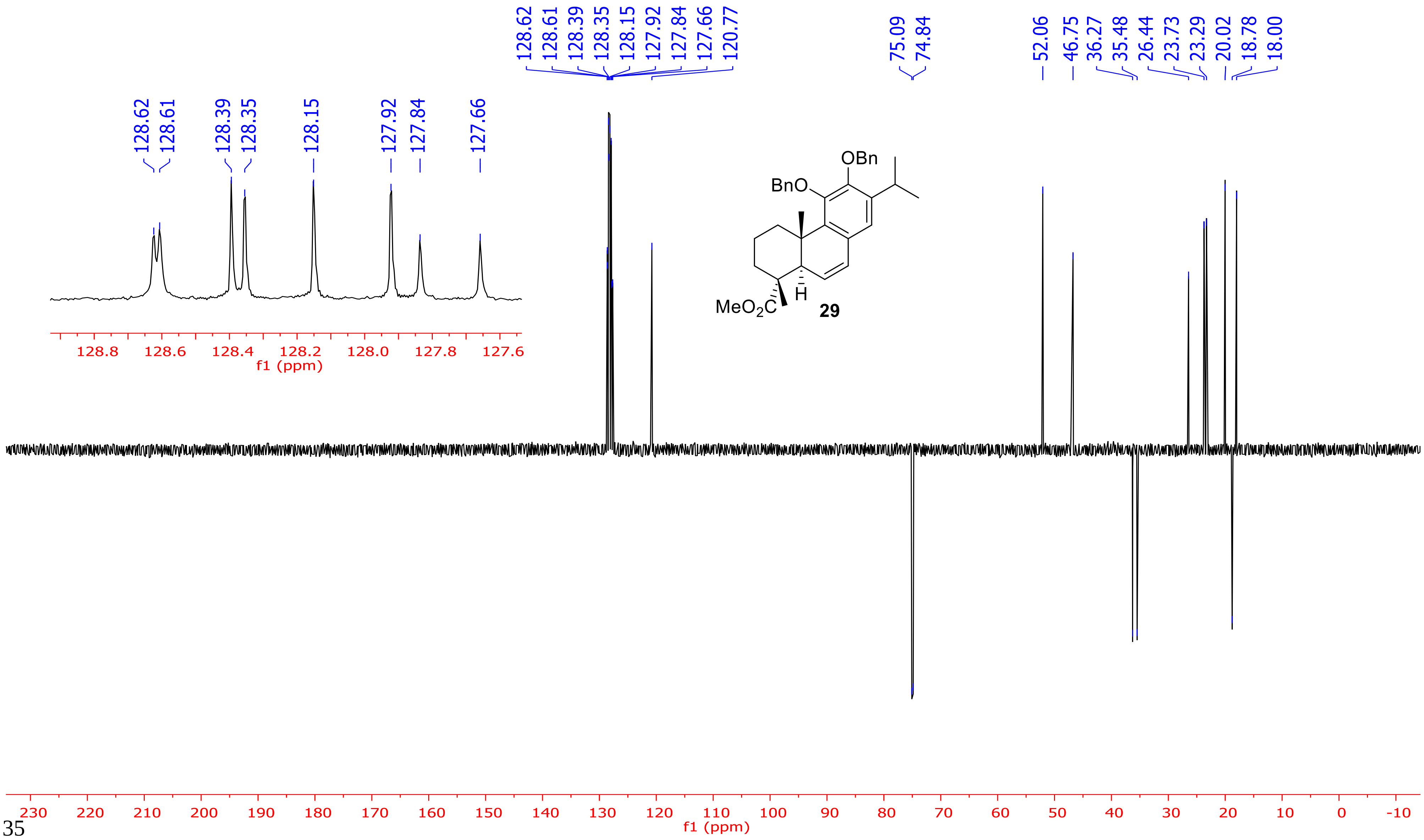
128.62
128.61
128.39
128.35
128.15
127.92
127.84
127.66
120.77
75.09
74.84
52.06
46.75
36.27
35.48
26.44
23.73
23.29
20.02
18.78
18.00
OBn
BnO
H
MeO2C
29
f1 (ppm)

35

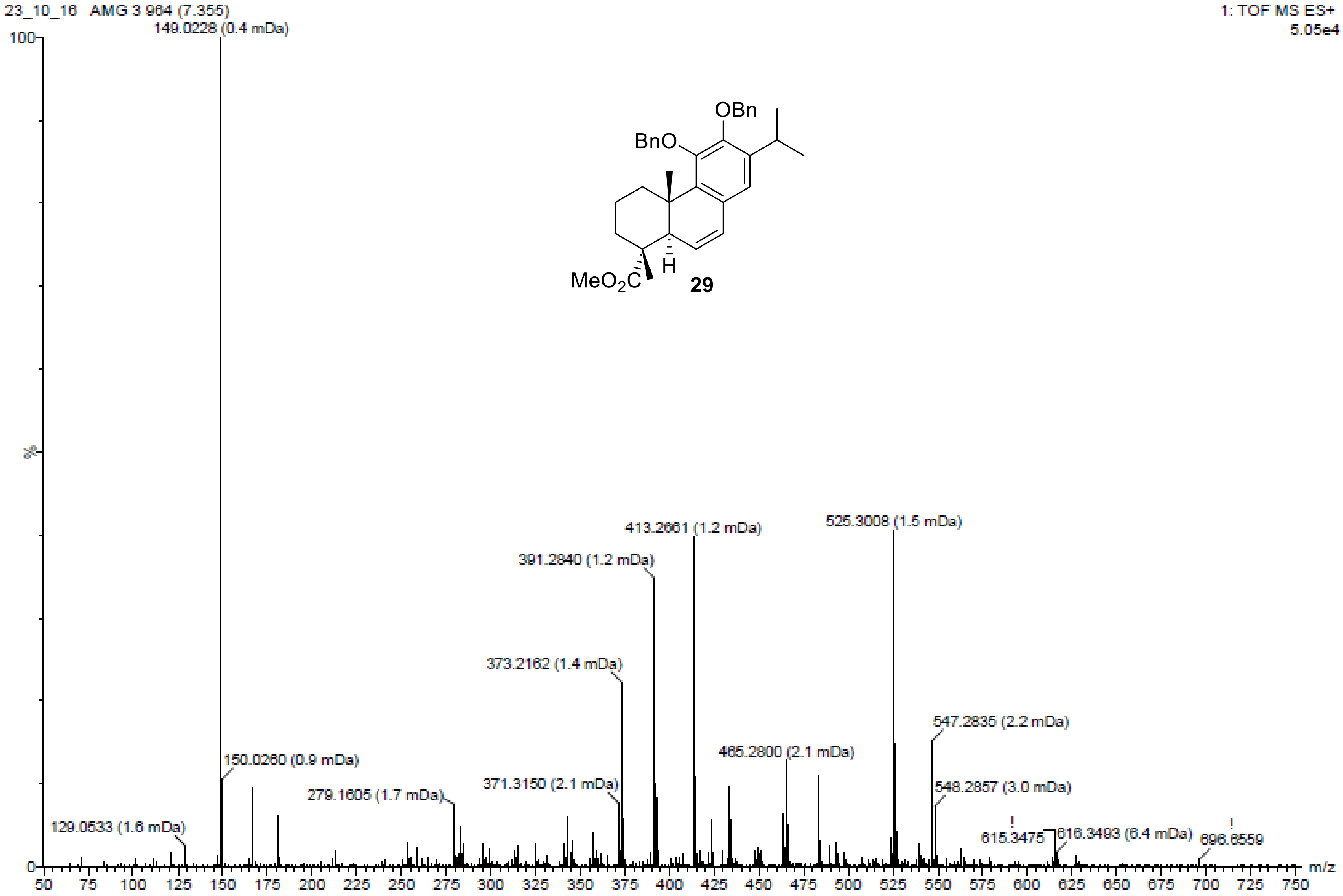

23_10_16 AMG 3 964 (7.355)
1: TOF MS ES+
5.05e4
149.0228 (0.4 mDa)
OBn
BnO
H
MeO2C
29
413.2661 (1.2 mDa)
525.3008 (1.5 mDa)
391.2840 (1.2 mDa)
373.2162 (1.4 mDa)
547.2835 (2.2 mDa)
465.2800 (2.1 mDa)
150.0260 (0.9 mDa)
548.2857 (3.0 mDa)
371.3150 (2.1 mDa)
279.1605 (1.7 mDa)
129.0533 (1.6 mDa)
615.3475
616.3493 (6.4 mDa)
696.6559
%
m/z

36

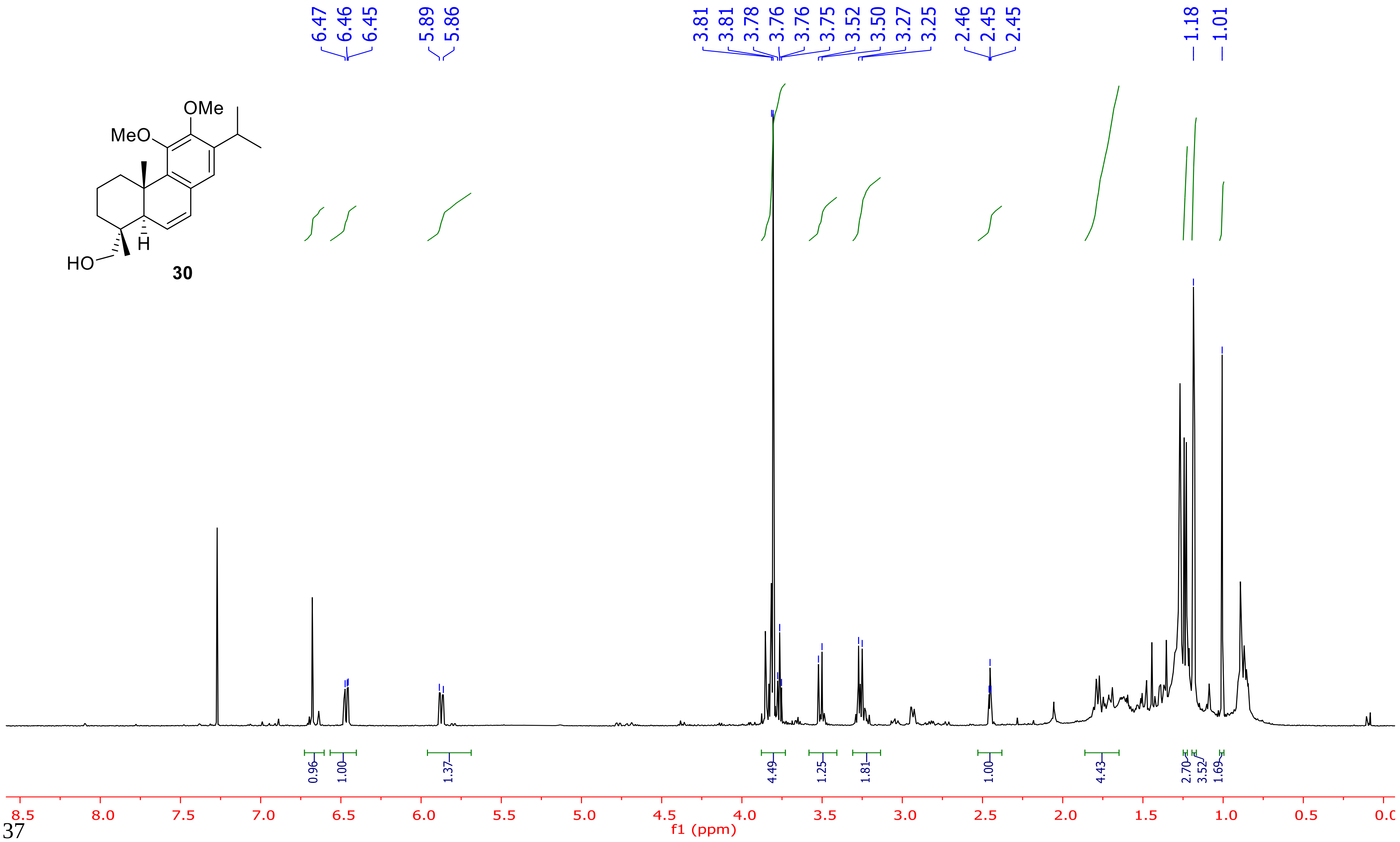
6.47
6.46
6.45
5.89
5.86
3.81
3.81
3.78
3.76
3.76
3.75
3.52
3.50
3.27
3.25
2.46
2.45
2.45
1.18
1.01
OMe
MeO
H
HO
30
0.96
1.00
1.37
4.49
1.25
1.81
1.00
4.43
2.70
3.52
1.69
8.5
8.0
7.5
7.0
6.5
6.0
5.5
5.0
4.5
4.0
3.5
3.0
2.5
2.0
1.5
1.0
0.5
f1 (ppm)

37

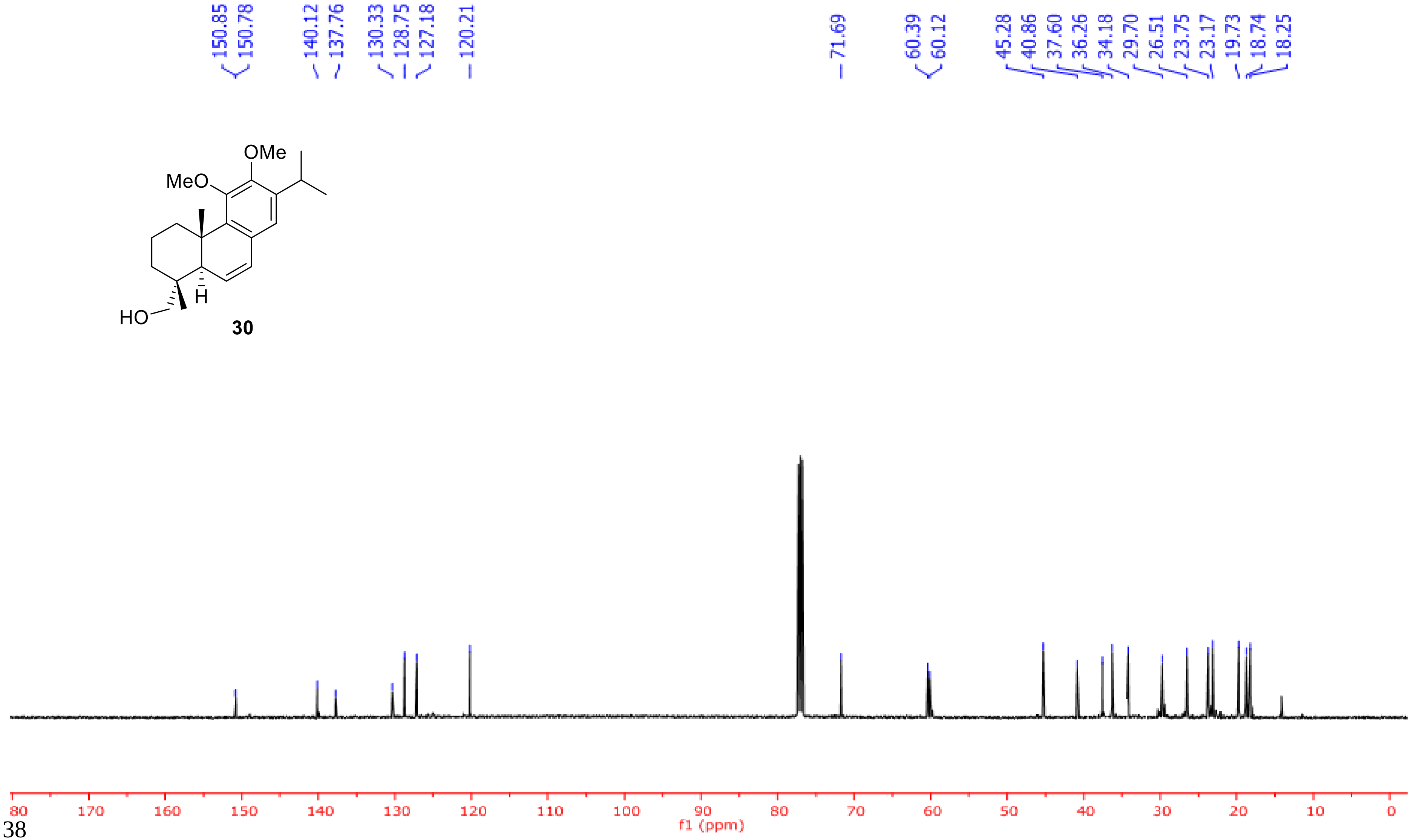
150.85
150.78
140.12
137.76
130.33
128.75
127.18
120.21
71.69
60.39
60.12
45.28
40.86
37.60
36.26
34.18
29.70
26.51
23.75
23.17
19.73
18.74
18.25
OMe
MeO
H
HO
30
f1 (ppm)

38

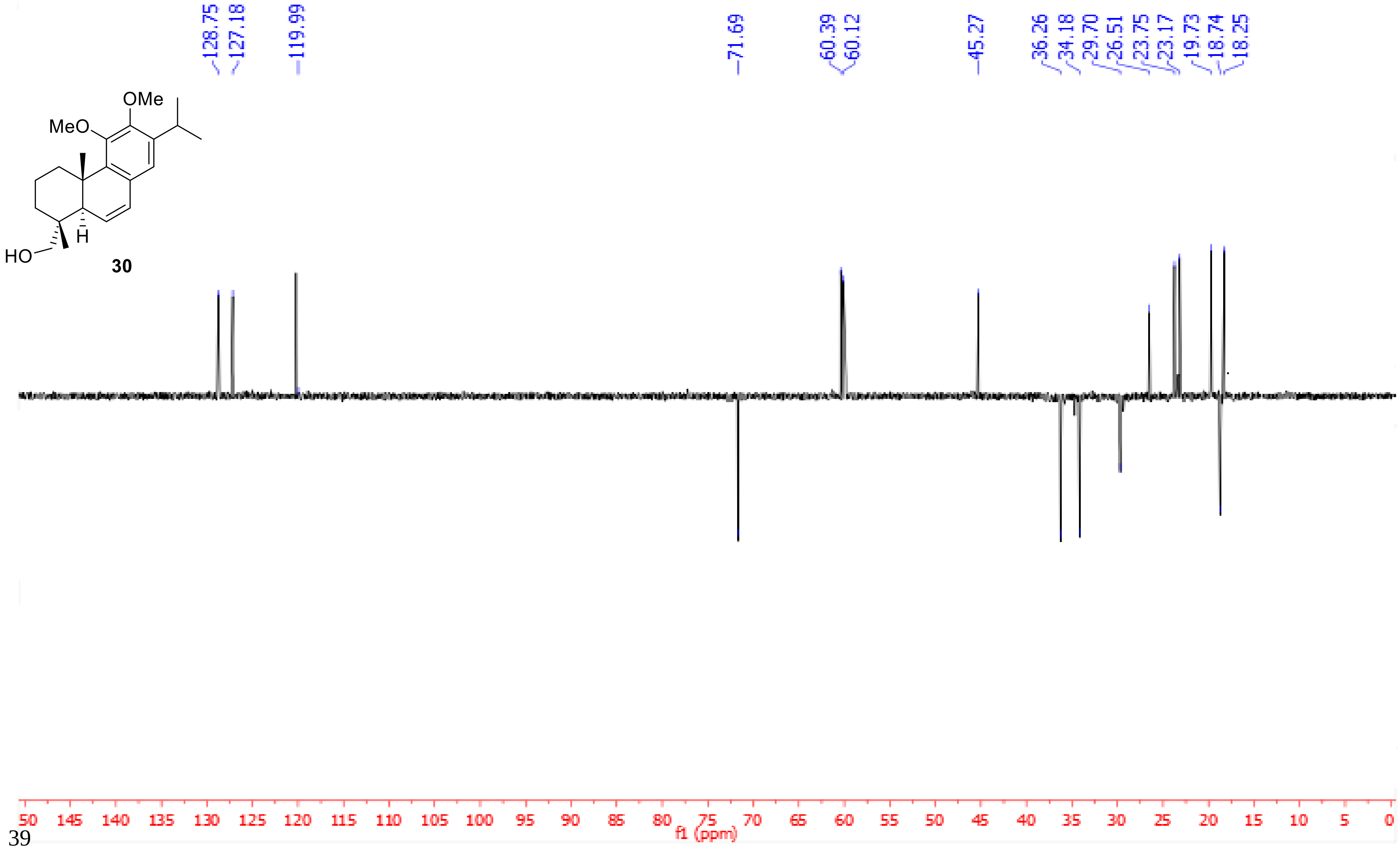
128.75
127.18
119.99
71.69
60.39
60.12
45.27
36.26
34.18
29.70
26.51
23.75
23.17
19.73
18.74
18.25
OMe
MeO
H
HO
30
50 145 140 135 130 125 120 115 110 105 100 95 90 85 80 75 70 65 60 55 50 45 40 35 30 25 20 15 10 5 0
f1 (ppm)

39

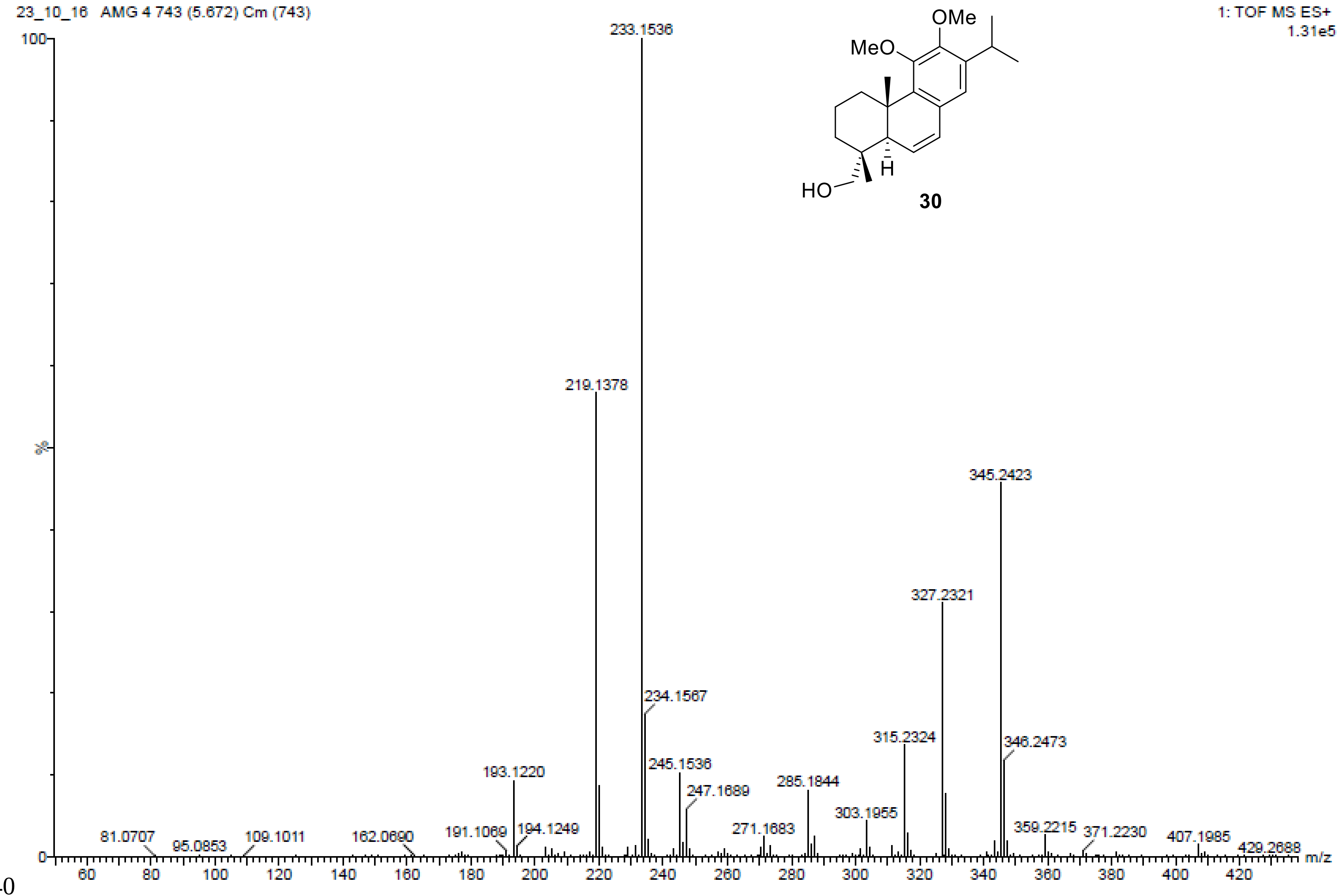

23_10_16 AMG 4 743 (5.672) Cm (743)
1: TOF MS ES+
1.31e5
OMe
MeO
HO
H
30
233.1536
219.1378
345.2423
327.2321
234.1567
315.2324
346.2473
245.1536
193.1220
247.1689
285.1844
303.1955
271.1683
81.0707
95.0853
109.1011
162.0690
191.1069
194.1249
359.2215
371.2230
407.1985
429.2688
%
m/z

40

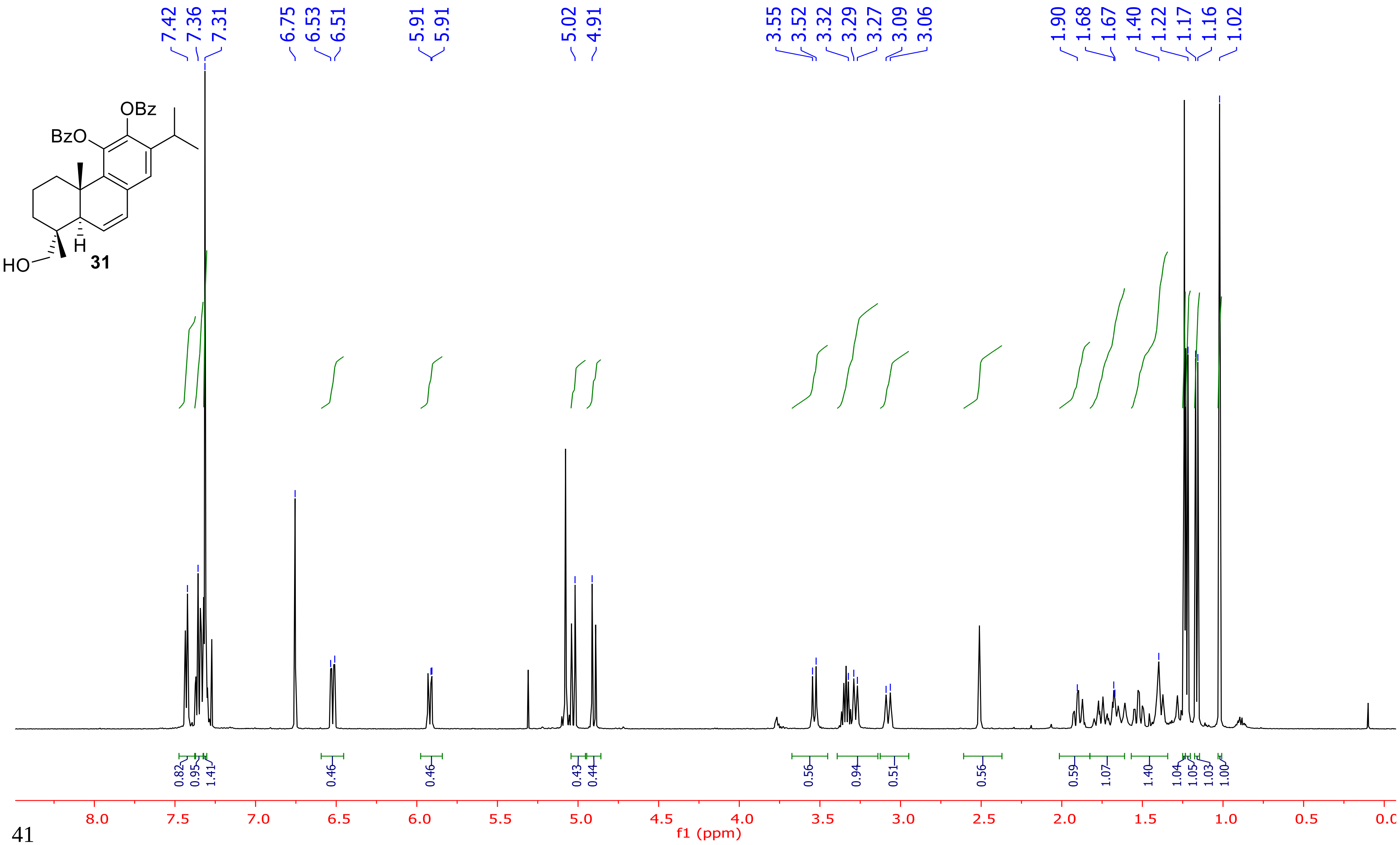
7.42
7.36
7.31
6.75
6.53
6.51
5.91
5.91
5.02
4.91
3.55
3.52
3.32
3.29
3.27
3.09
3.06
1.90
1.68
1.67
1.40
1.22
1.17
1.16
1.02
OBz
BzO
HO
H
31
0.82
0.95
1.41
0.46
0.46
0.43
0.44
0.56
0.94
0.51
0.56
0.59
1.07
1.40
1.04
1.05
1.03
1.00
8.0
7.5
7.0
6.5
6.0
5.5
5.0
4.5
4.0
3.5
3.0
2.5
2.0
1.5
1.0
0.5
0.0
f1 (ppm)

41

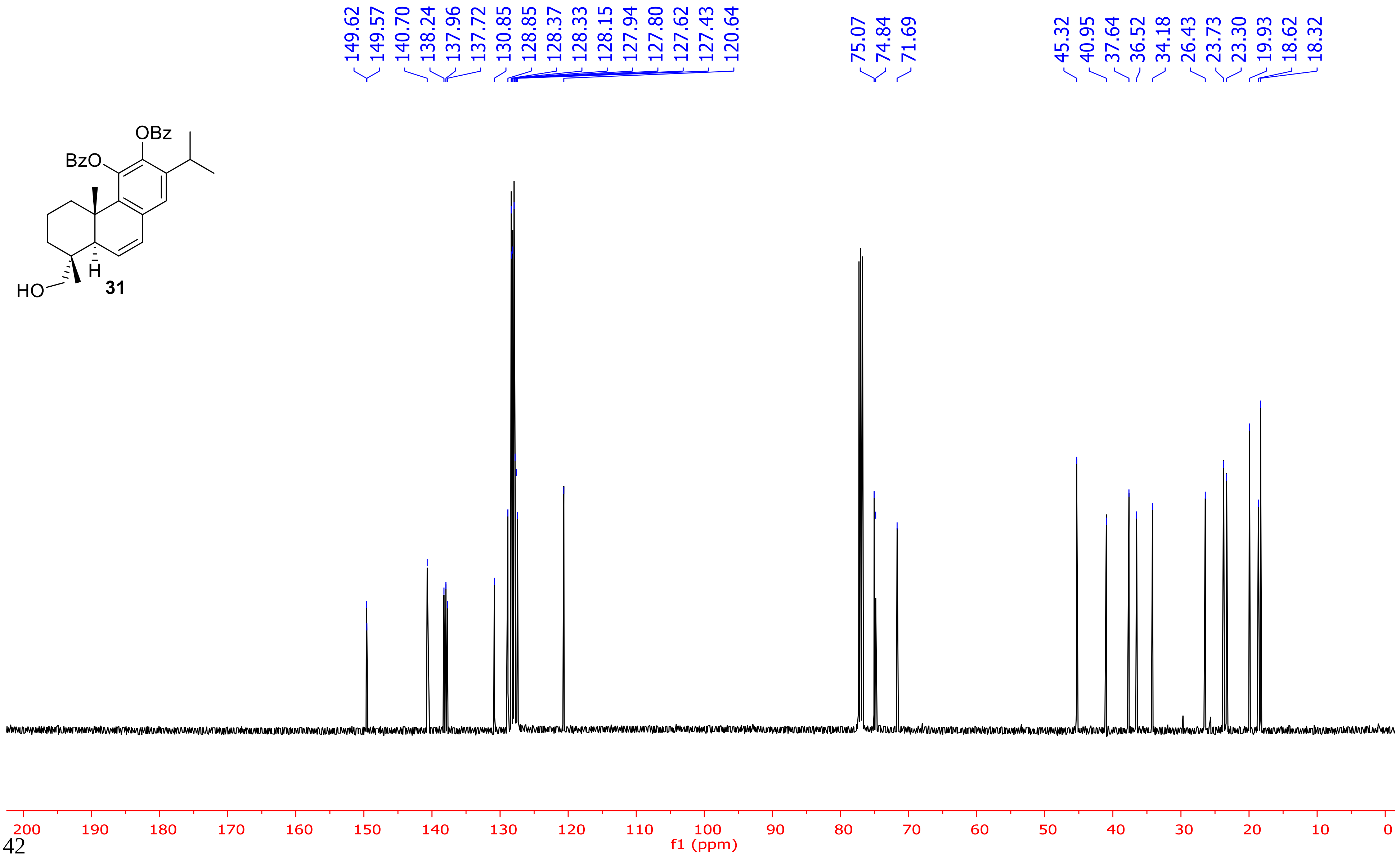
149.62
149.57
140.70
138.24
137.96
137.72
130.85
128.85
128.37
128.33
128.15
127.94
127.80
127.62
127.43
120.64
75.07
74.84
71.69
45.32
40.95
37.64
36.52
34.18
26.43
23.73
23.30
19.93
18.62
18.32
OBz
BzO
H
HO
31
f1 (ppm)

42

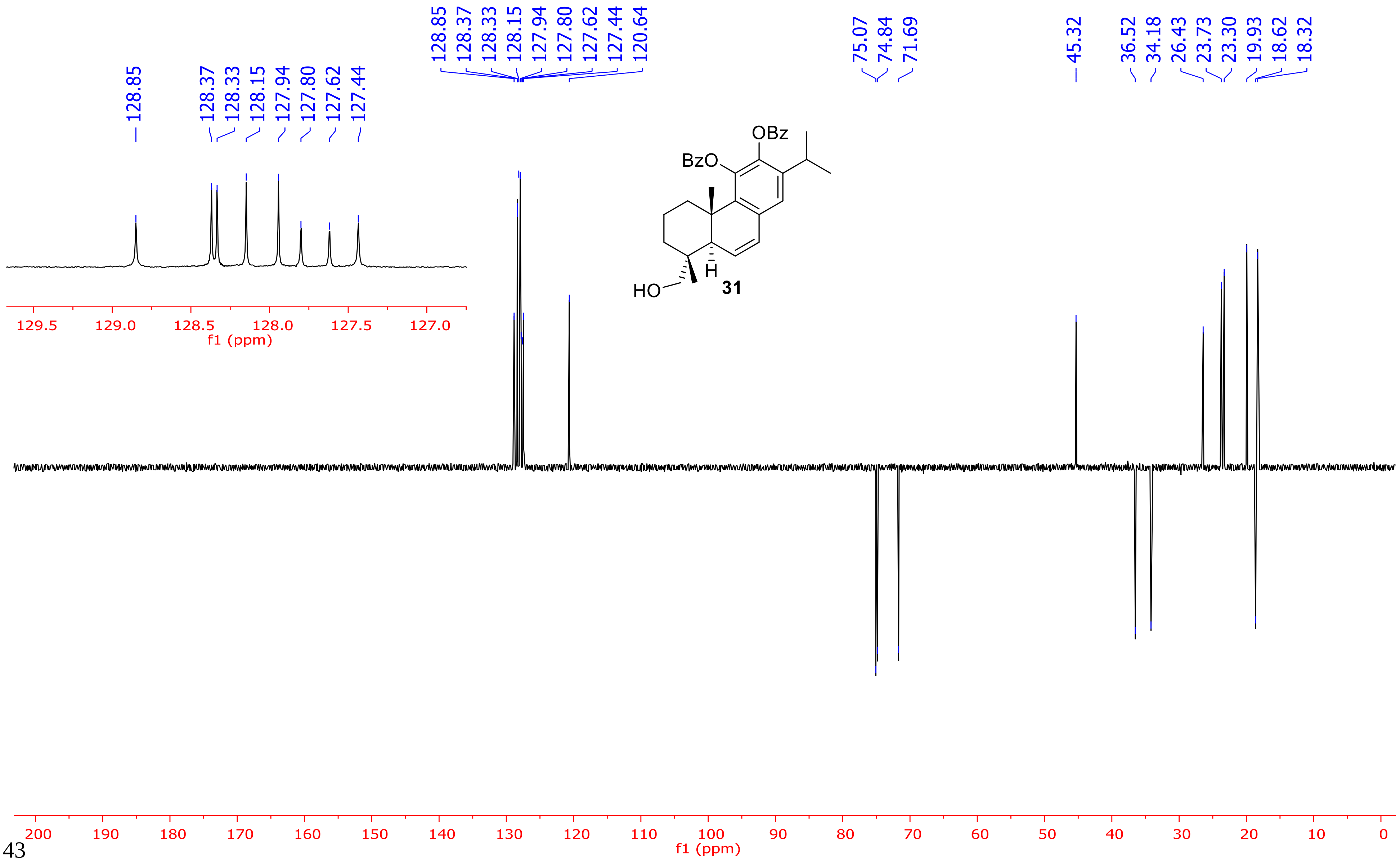
128.85
128.37
128.33
128.15
127.94
127.80
127.62
127.44
120.64
75.07
74.84
71.69
45.32
36.52
34.18
26.43
23.73
23.30
19.93
18.62
18.32
OBz
BzO
HO
H
31
129.5 129.0 128.5 128.0 127.5 127.0
f1 (ppm)
200 190 180 170 160 150 140 130 120 110 100 90 80 70 60 50 40 30 20 10 0
f1 (ppm)

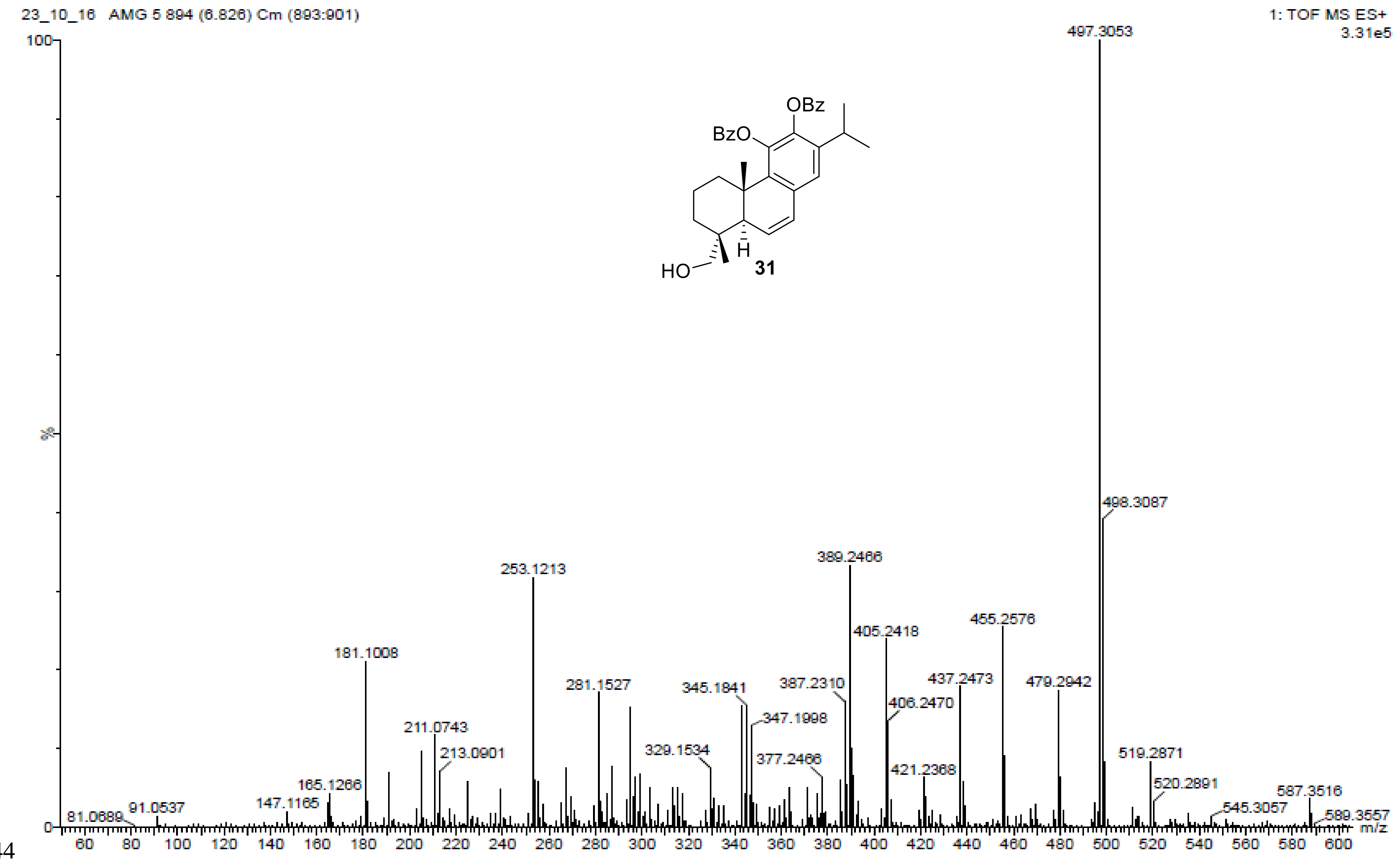

23_10_16 AMG 5 894 (6.826) Cm (893:901)
1: TOF MS ES+
3.31e5
497.3053
498.3087
389.2466
253.1213
455.2576
405.2418
181.1008
437.2473
479.2942
281.1527
345.1841
387.2310
406.2470
347.1998
211.0743
213.0901
329.1534
377.2466
519.2871
421.2368
165.1266
520.2891
587.3516
147.1165
91.0537
545.3057
81.0689
589.3557
OBz
BzO
H
HO
31
%
m/z

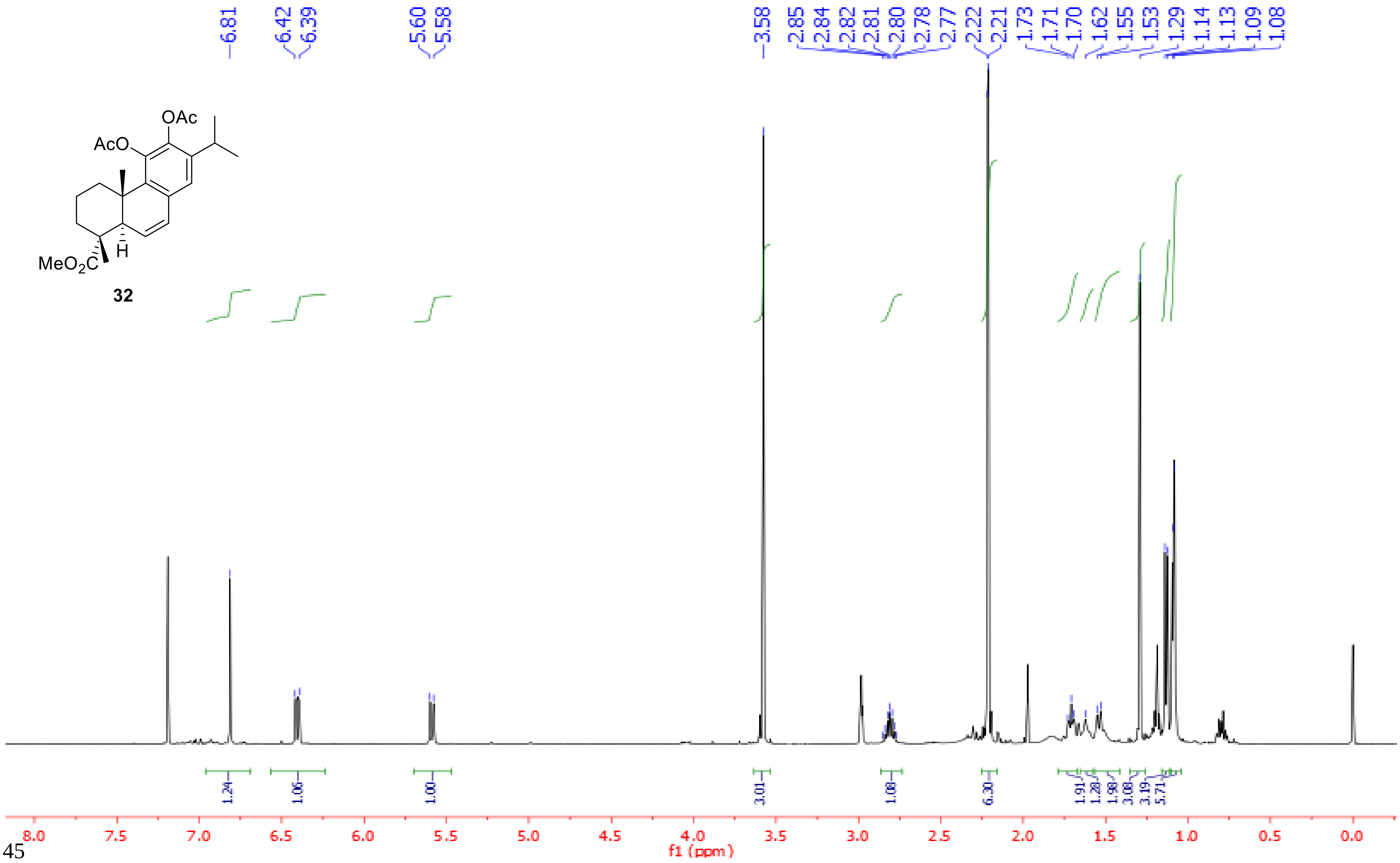
OAc
AcO
H
MeO2C
32
6.81
6.42
6.39
5.60
5.58
3.58
2.85
2.84
2.82
2.81
2.80
2.78
2.77
2.22
2.21
1.73
1.71
1.70
1.62
1.55
1.53
1.29
1.14
1.13
1.09
1.08
1.24
1.06
1.00
3.01
1.08
6.30
1.91
1.28
1.98
3.08
3.19
5.71
8.0
7.5
7.0
6.5
6.0
5.5
5.0
4.5
4.0
3.5
3.0
2.5
2.0
1.5
1.0
0.5
0.0
f1 (ppm)

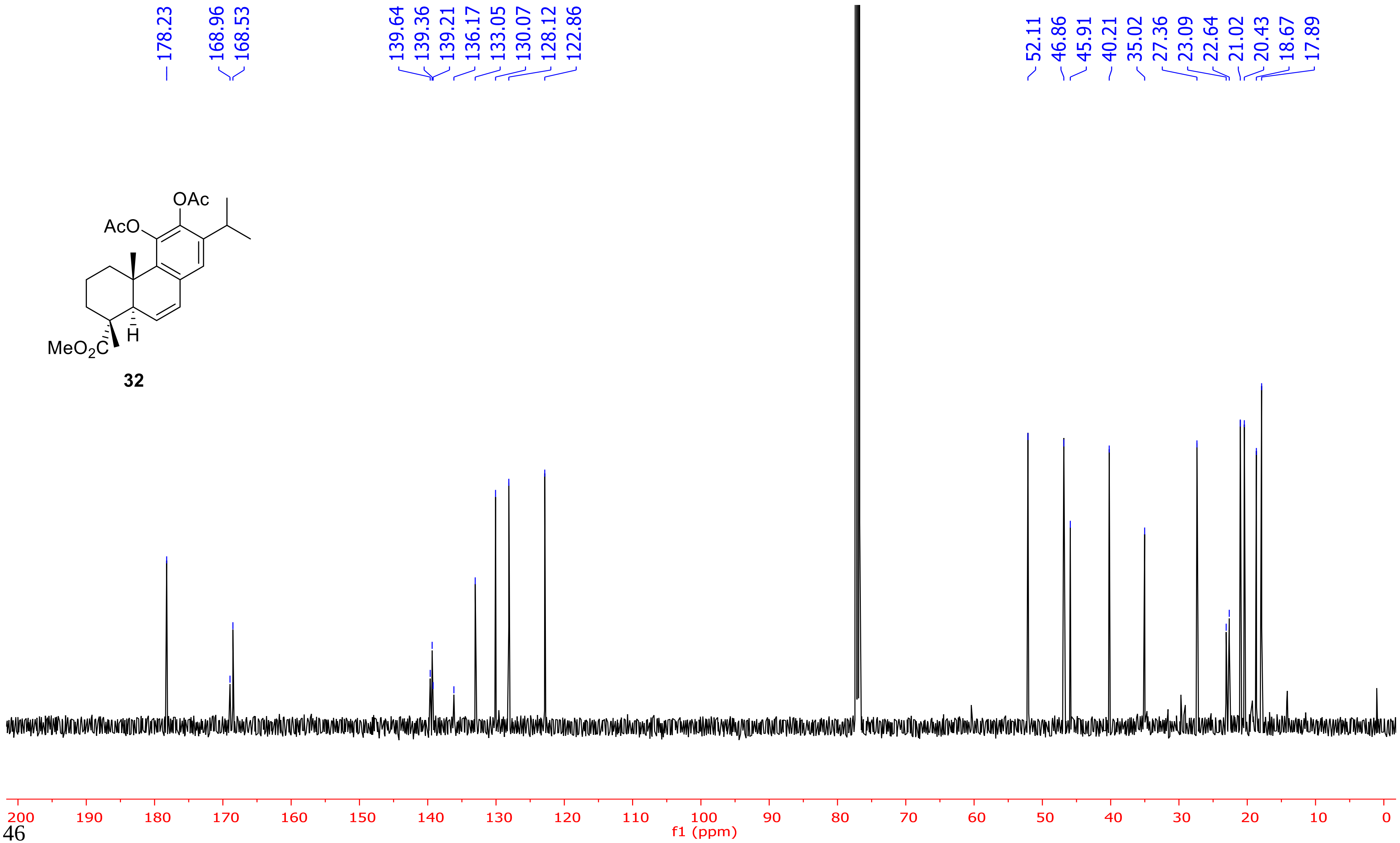
178.23
168.96
168.53
139.64
139.36
139.21
136.17
133.05
130.07
128.12
122.86
52.11
46.86
45.91
40.21
35.02
27.36
23.09
22.64
21.02
20.43
18.67
17.89
OAc
AcO
H
MeO2C
32
200 190 180 170 160 150 140 130 120 110 100 90 80 70 60 50 40 30 20 10 0
f1 (ppm)

46

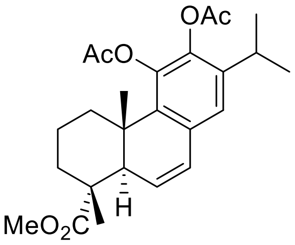
OAc
AcO
H
MeO2C

32

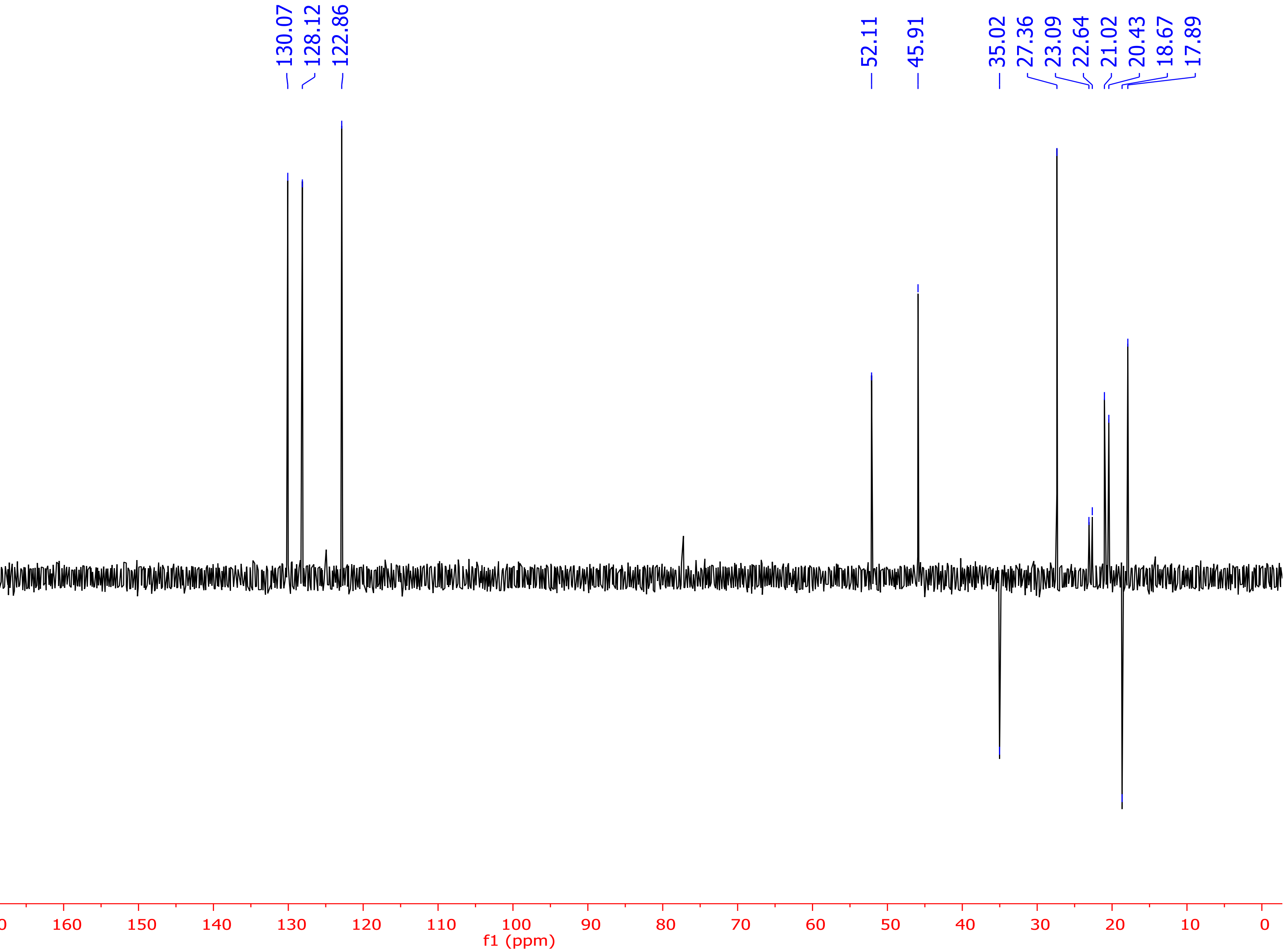
130.07
128.12
122.86
52.11
45.91
35.02
27.36
23.09
22.64
21.02
20.43
18.67
17.89
200 190 180 170 160 150 140 130 120 110 100 90 80 70 60 50 40 30 20 10 0
f1 (ppm)

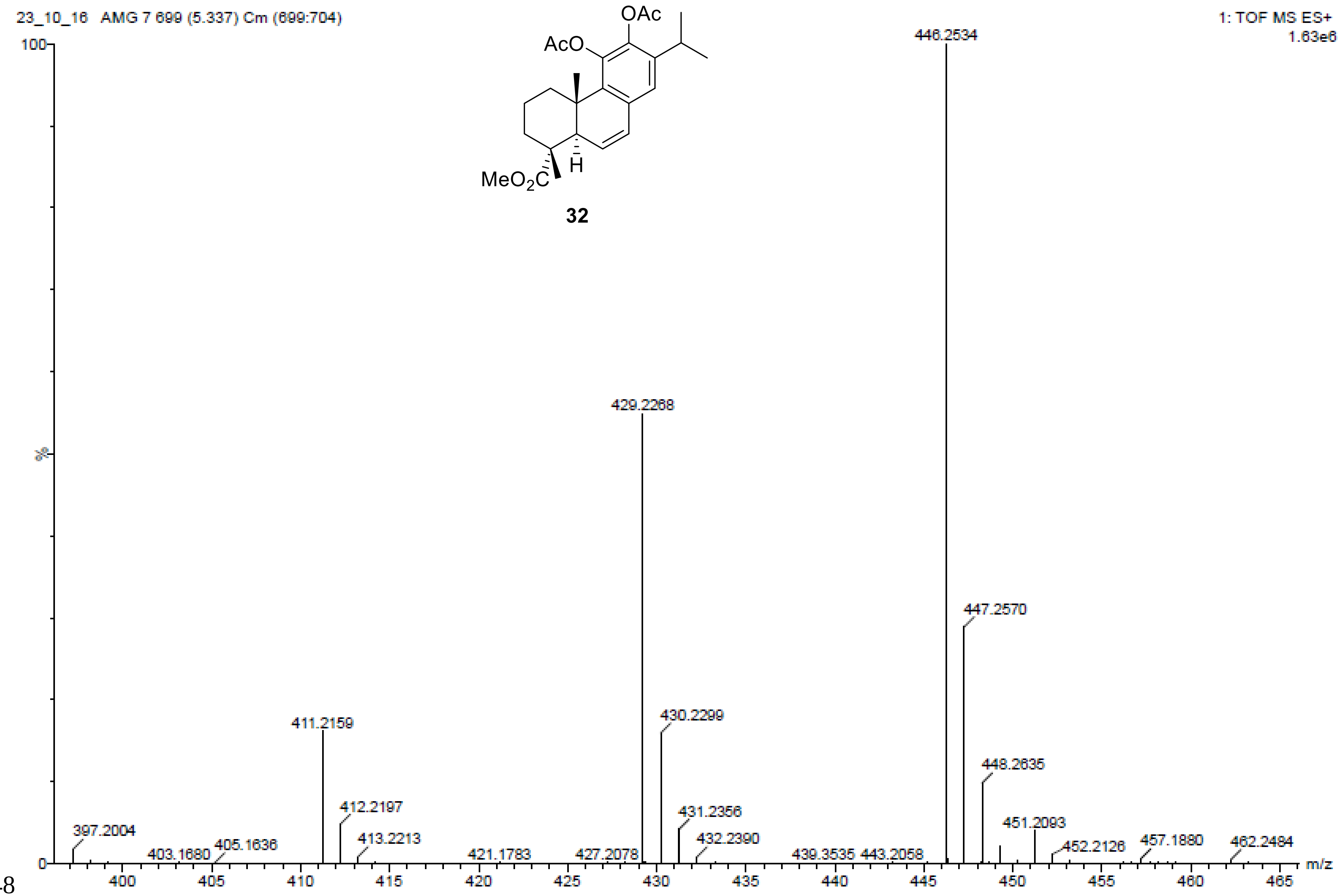
23_10_16 AMG 7 699 (5.337) Cm (699:704)
1: TOF MS ES+
1.63e6
OAc
AcO
H
MeO_2C
32
100
%
0
446.2534
429.2268
447.2570
411.2159
430.2299
448.2635
412.2197
431.2356
451.2093
397.2004
413.2213
432.2390
452.2126
457.1880
462.2484
403.1680
405.1636
421.1783
427.2078
439.3535
443.2058
400
405
410
415
420
425
430
435
440
445
450
455
460
465
m/z

48

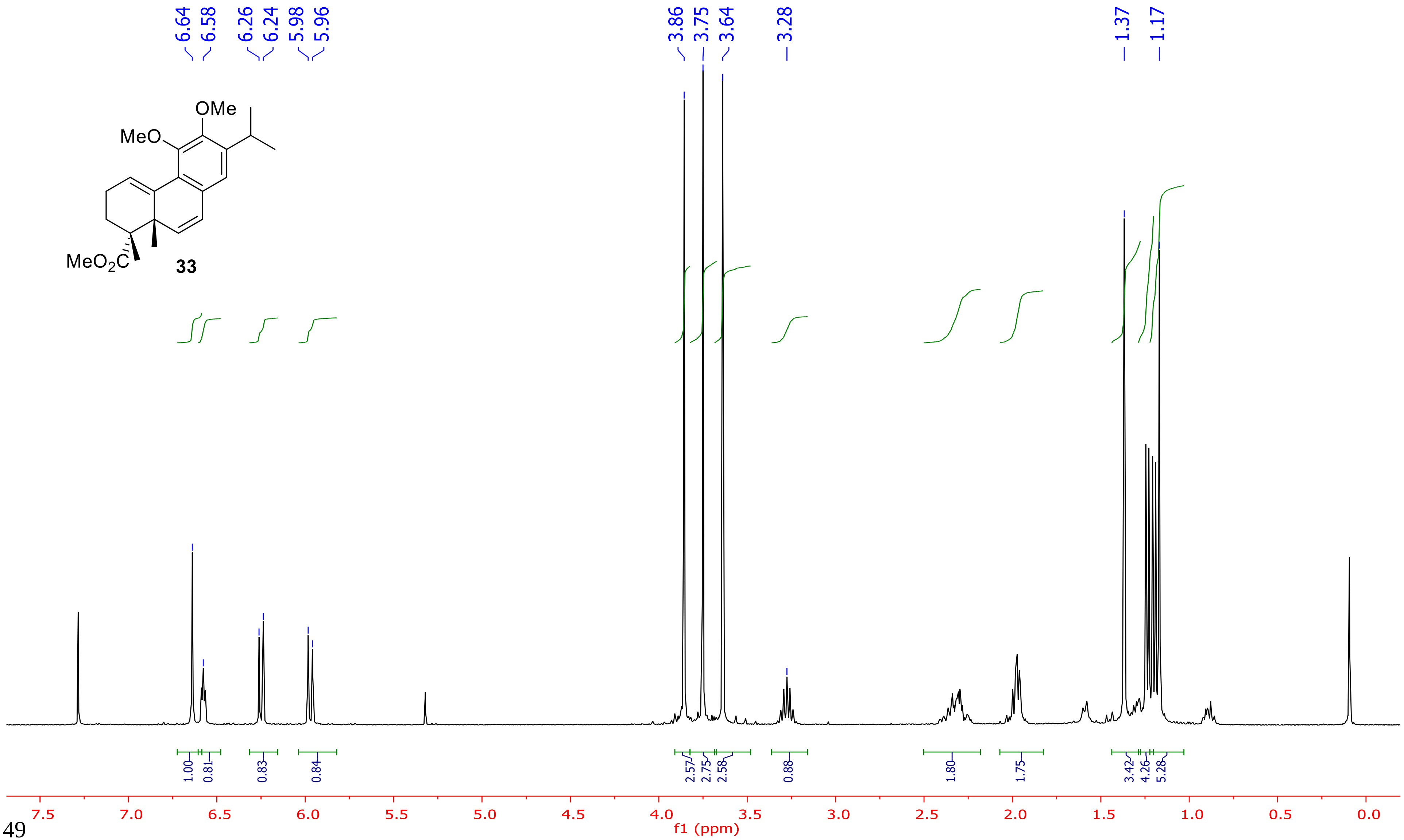
6.64
6.58
6.26
6.24
5.98
5.96
3.86
3.75
3.64
3.28
1.37
1.17
OMe
MeO
MeO_2C
33
1.00
0.81
0.83
0.84
2.57
2.75
2.58
0.88
1.80
1.75
3.42
4.26
5.28
7.5
7.0
6.5
6.0
5.5
5.0
4.5
4.0
3.5
3.0
2.5
2.0
1.5
1.0
0.5
0.0
f1 (ppm)

49

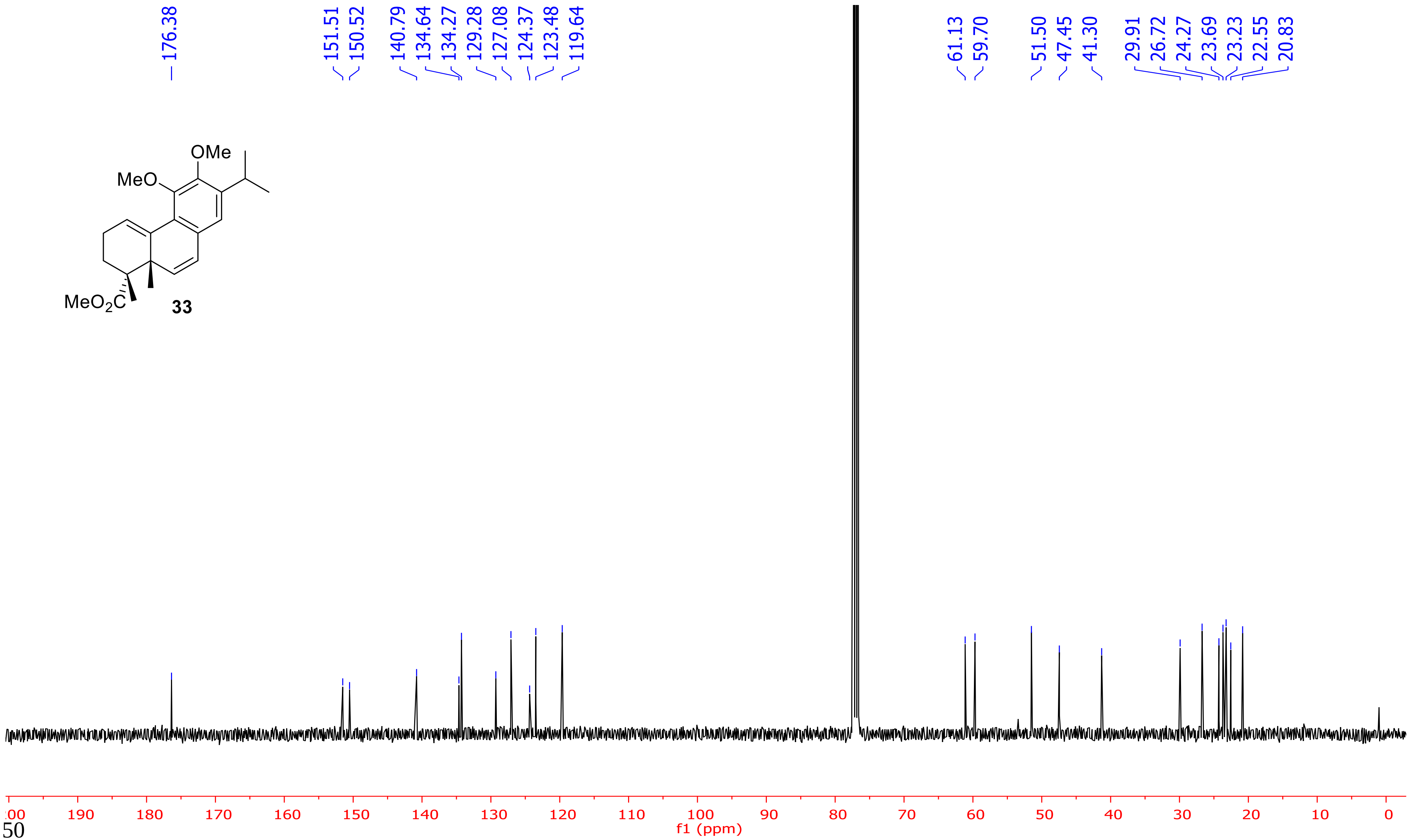
176.38
151.51
150.52
140.79
134.64
134.27
129.28
127.08
124.37
123.48
119.64
61.13
59.70
51.50
47.45
41.30
29.91
26.72
24.27
23.69
23.23
22.55
20.83
OMe
MeO
MeO_2C
33
f1 (ppm)

50

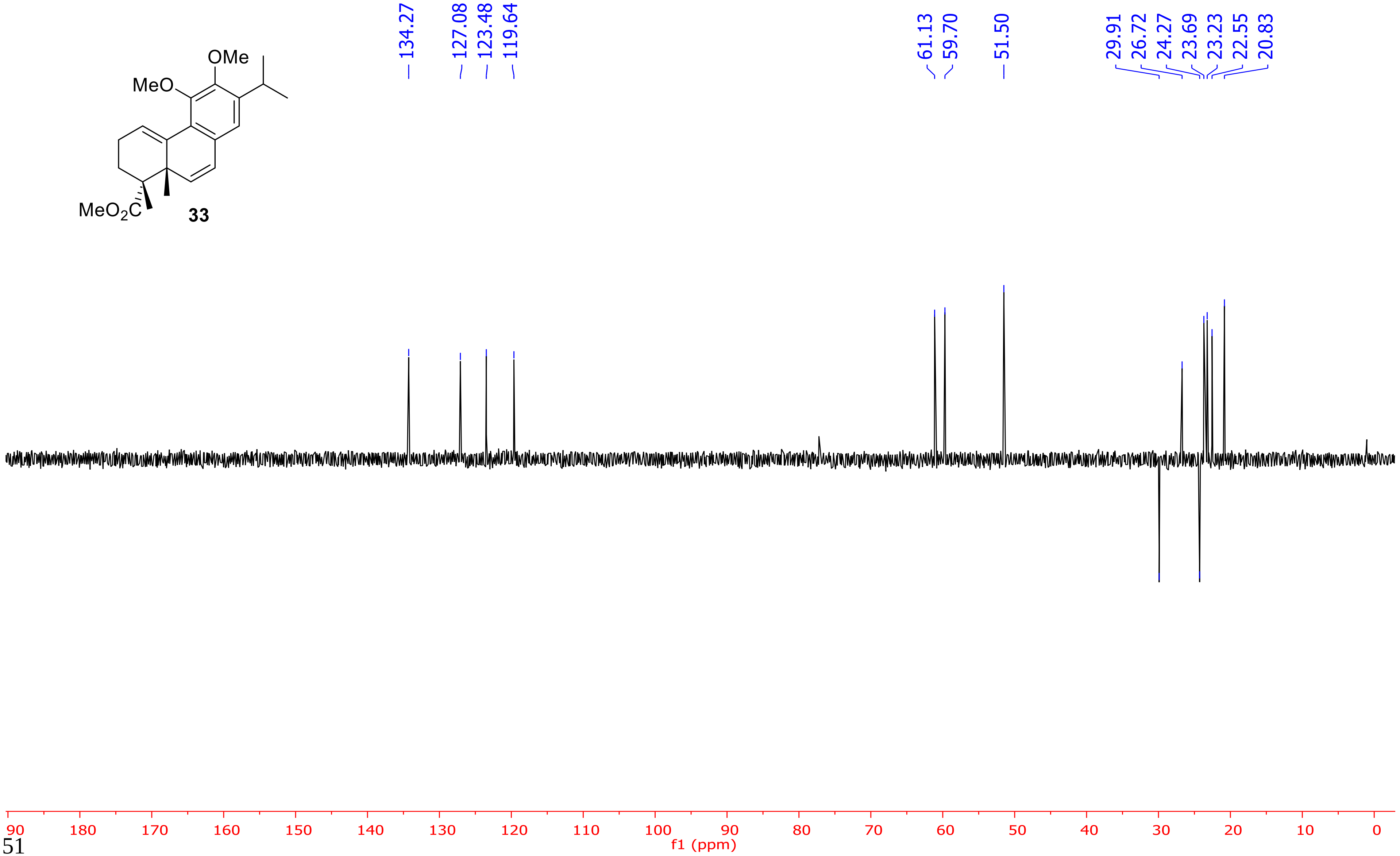
OMe
MeO
MeO2C
33
134.27
127.08
123.48
119.64
61.13
59.70
51.50
29.91
26.72
24.27
23.69
23.23
22.55
20.83
90
180
170
160
150
140
130
120
110
100
90
80
70
60
50
40
30
20
10
0
f1 (ppm)

51

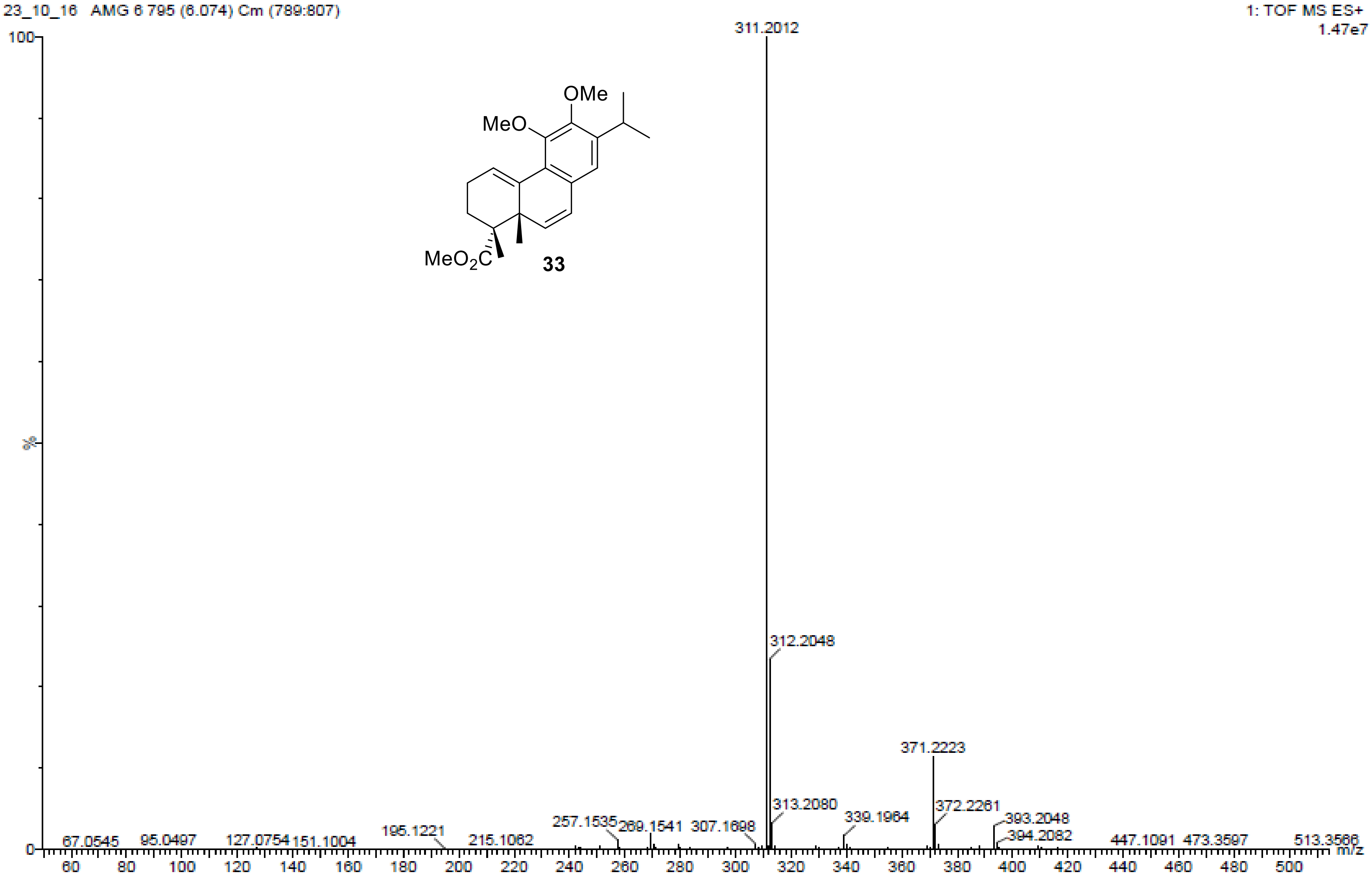
23_10_16 AMG 6 795 (6.074) Cm (789:807)
1: TOF MS ES+
1.47e7
311.2012
312.2048
313.2080
371.2223
372.2261
393.2048
394.2082
339.1964
307.1698
269.1541
257.1535
215.1062
195.1221
151.1004
127.0754
95.0497
67.0545
447.1091
473.3597
513.3566
OMe
MeO
MeO_2C
33
%
m/z

52

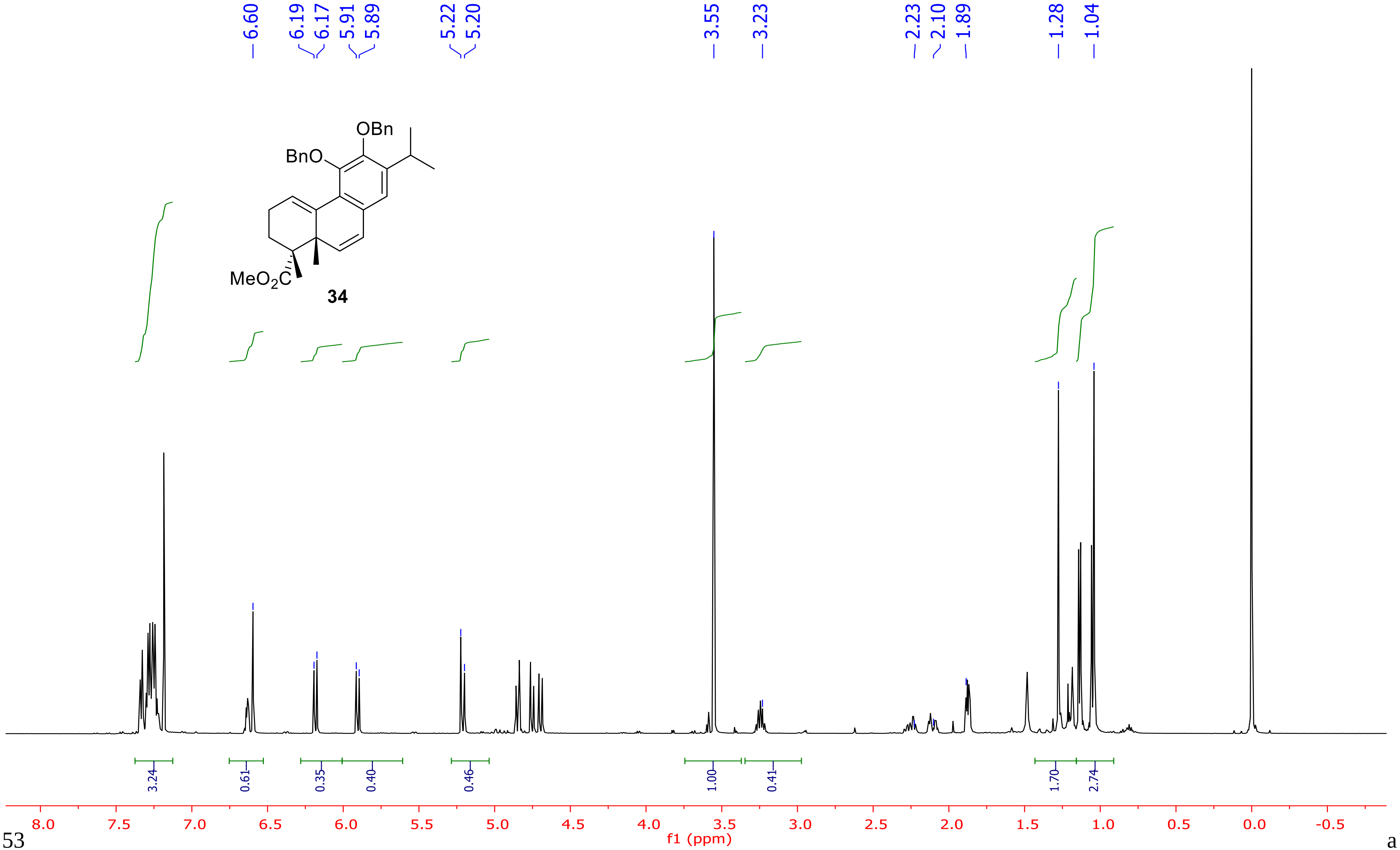

6.60
6.19
6.17
5.91
5.89
5.22
5.20
3.55
3.23
2.23
2.10
1.89
1.28
1.04
OBn
BnO
MeO2C
34
3.24
0.61
0.35
0.40
0.46
1.00
0.41
1.70
2.74
8.0 7.5 7.0 6.5 6.0 5.5 5.0 4.5 4.0 3.5 3.0 2.5 2.0 1.5 1.0 0.5 0.0 -0.5
f1 (ppm)

53 a

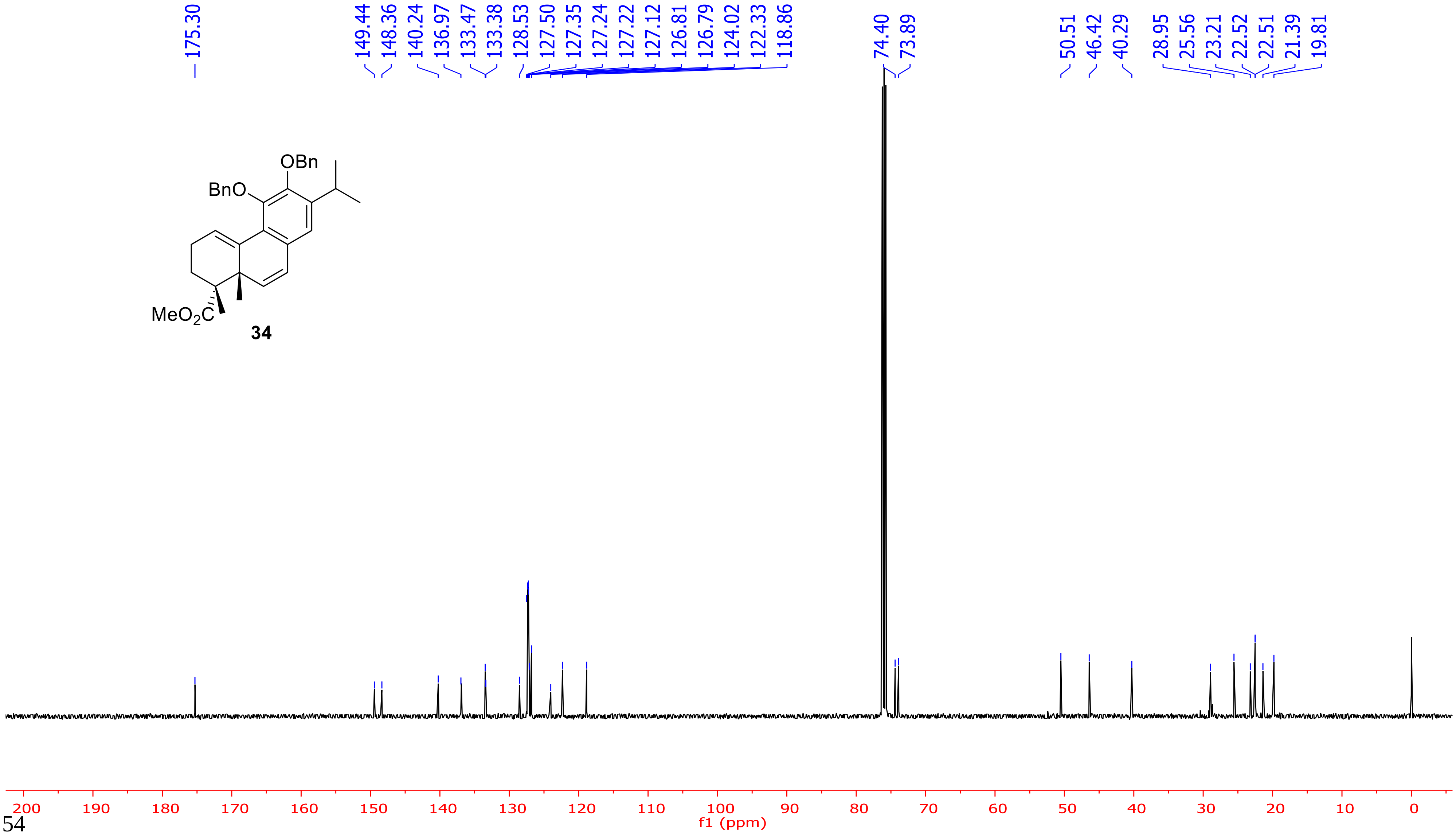
175.30
149.44
148.36
140.24
136.97
133.47
133.38
128.53
127.50
127.35
127.24
127.22
127.12
126.81
126.79
124.02
122.33
118.86
74.40
73.89
50.51
46.42
40.29
28.95
25.56
23.21
22.52
22.51
21.39
19.81
200 190 180 170 160 150 140 130 120 110 100 90 80 70 60 50 40 30 20 10 0
f1 (ppm)
OBn
BnO
MeO2C

34

54

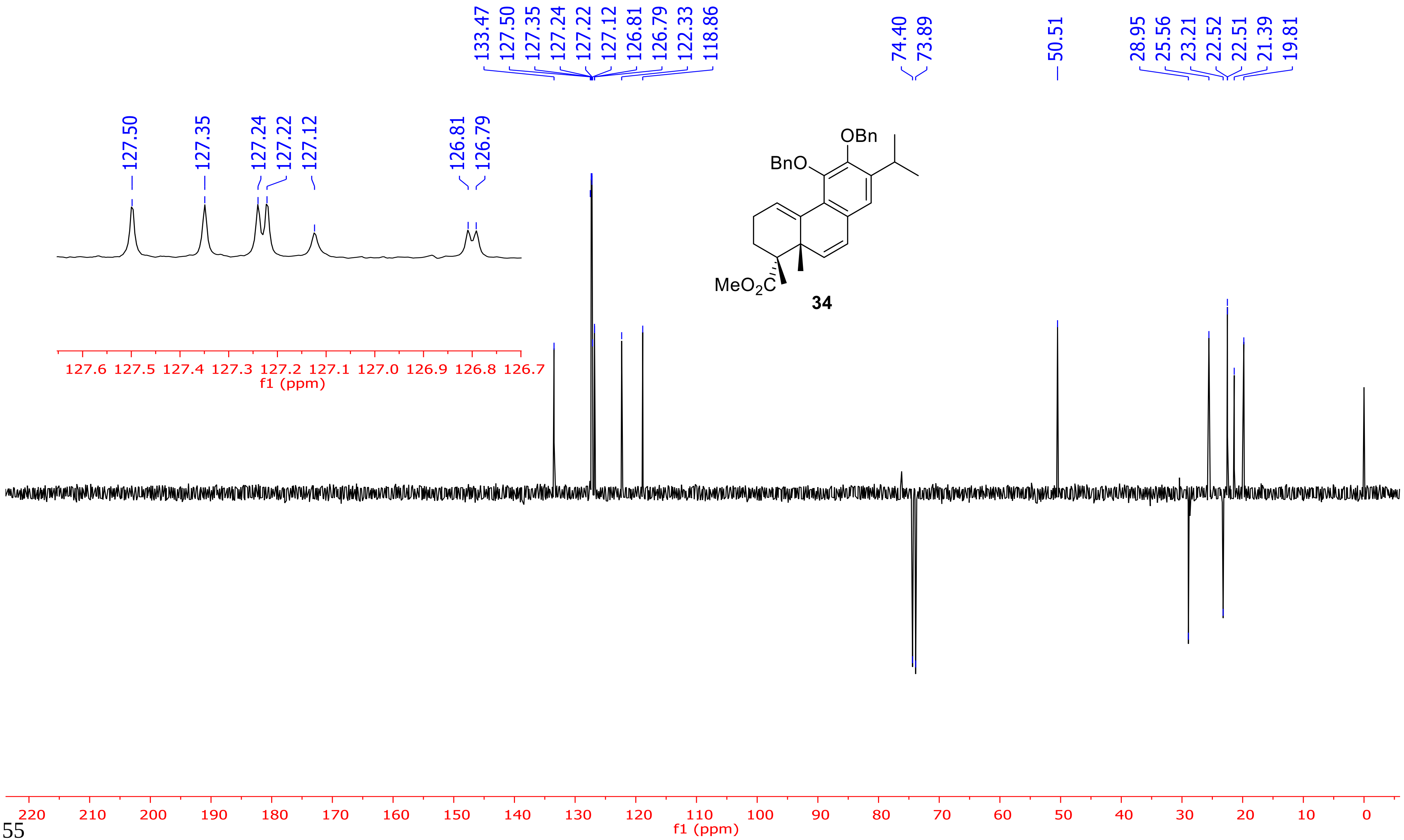
133.47
127.50
127.35
127.24
127.22
127.12
126.81
126.79
122.33
118.86
74.40
73.89
50.51
28.95
25.56
23.21
22.52
22.51
21.39
19.81
OBn
BnO
MeO2C
34
127.6 127.5 127.4 127.3 127.2 127.1 127.0 126.9 126.8 126.7
f1 (ppm)
220 210 200 190 180 170 160 150 140 130 120 110 100 90 80 70 60 50 40 30 20 10 0
f1 (ppm)

55

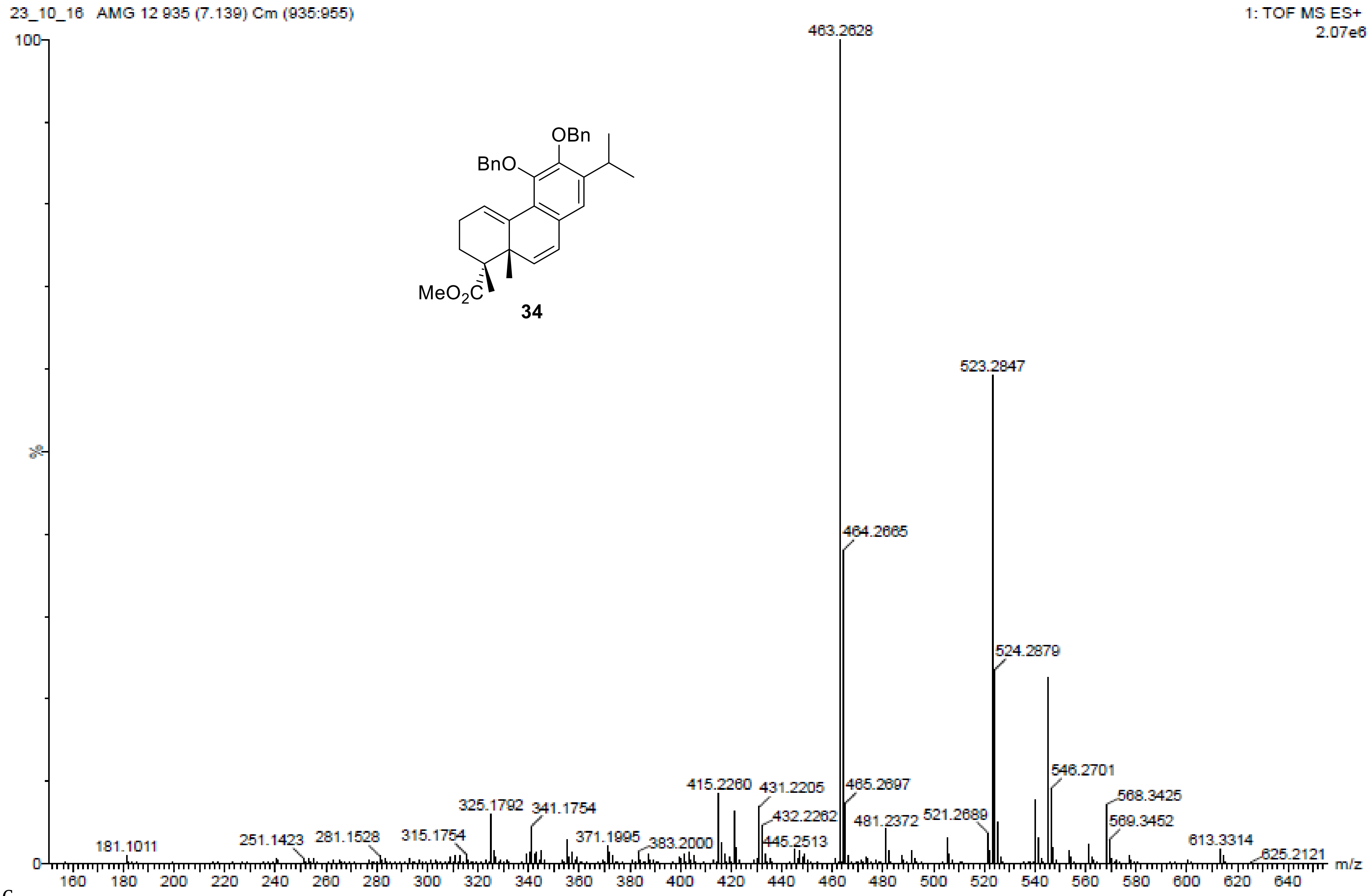

23_10_16 AMG 12 935 (7.139) Cm (935:955)
1: TOF MS ES+
2.07e6
463.2628
523.2847
464.2665
524.2879
546.2701
568.3425
569.3452
613.3314
625.2121
415.2260
431.2205
432.2262
445.2513
465.2697
481.2372
521.2689
325.1792
341.1754
371.1995
383.2000
315.1754
281.1528
251.1423
181.1011
OBn
BnO
MeO2C
34
%
m/z

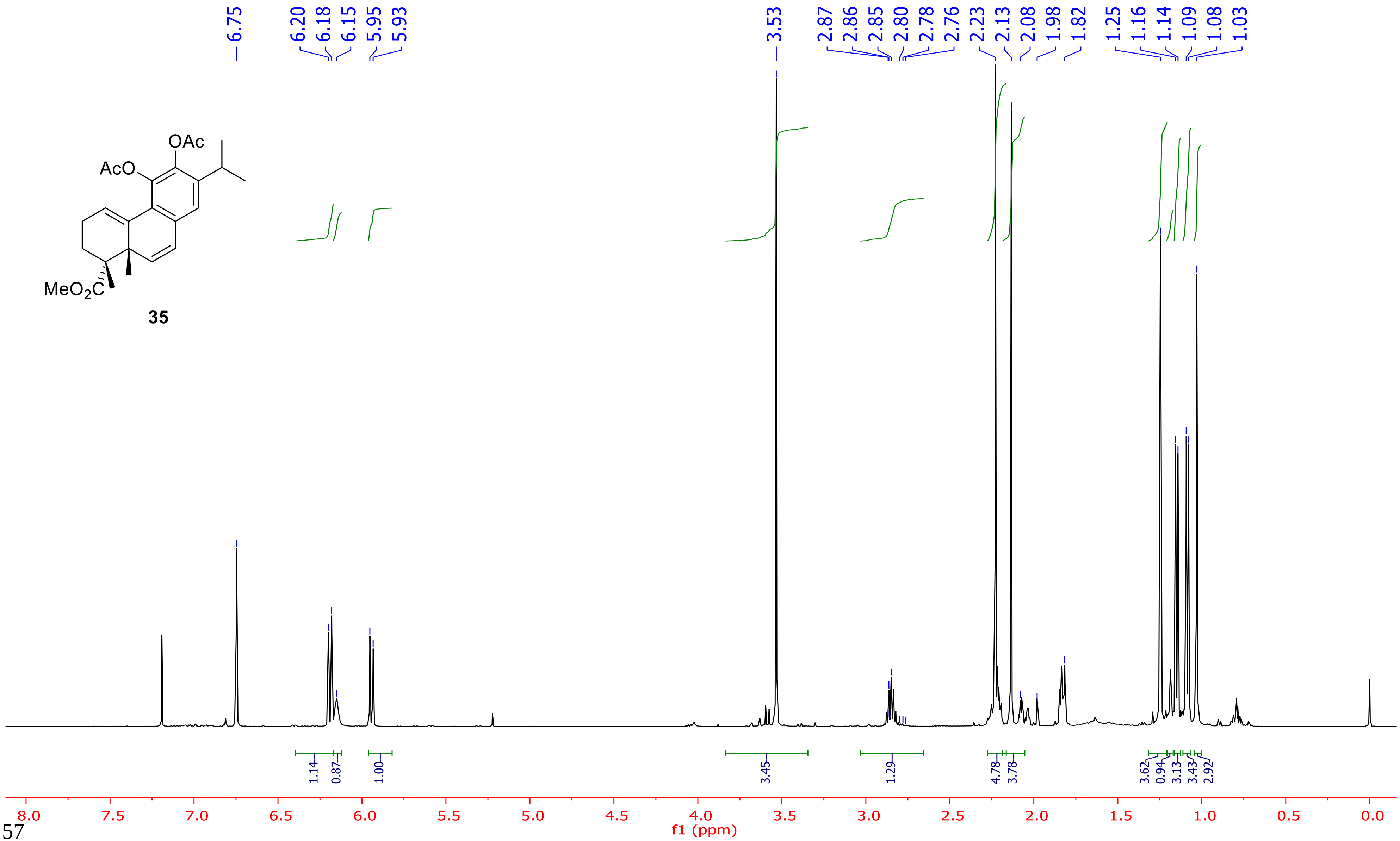
6.75
6.20
6.18
6.15
5.95
5.93
3.53
2.87
2.86
2.85
2.80
2.78
2.76
2.23
2.13
2.08
1.98
1.82
1.25
1.16
1.14
1.09
1.08
1.03
OAc
AcO
MeO2C
35
1.14
0.87
1.00
3.45
1.29
4.78
3.78
3.62
0.94
3.13
3.43
2.92
8.0
7.5
7.0
6.5
6.0
5.5
5.0
4.5
4.0
3.5
3.0
2.5
2.0
1.5
1.0
0.5
0.0
f1 (ppm)

57

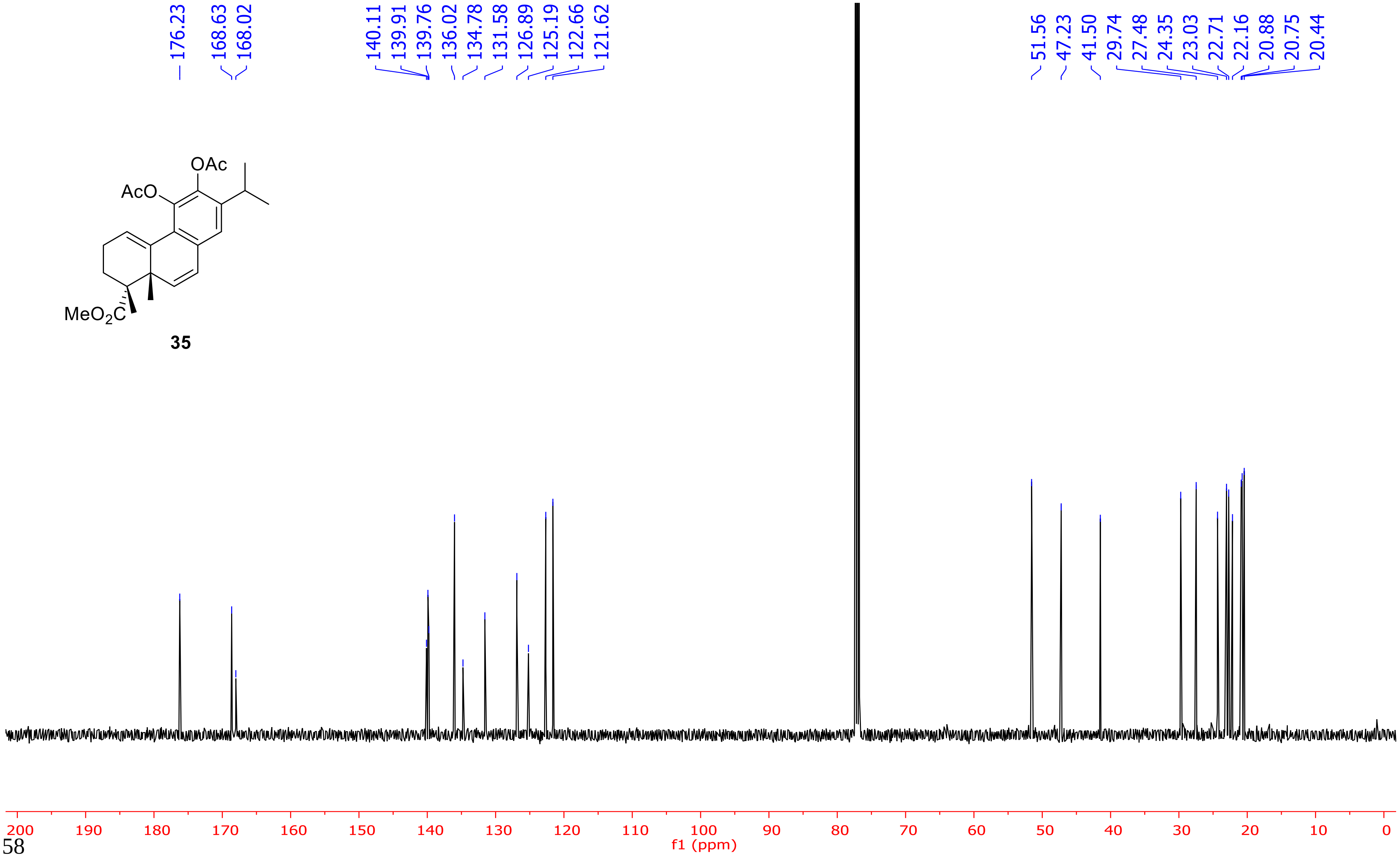
176.23
168.63
168.02
140.11
139.91
139.76
136.02
134.78
131.58
126.89
125.19
122.66
121.62
51.56
47.23
41.50
29.74
27.48
24.35
23.03
22.71
22.16
20.88
20.75
20.44
OAc
AcO
MeO2C
35
200
190
180
170
160
150
140
130
120
110
100
90
80
70
60
50
40
30
20
10
0
f1 (ppm)

58

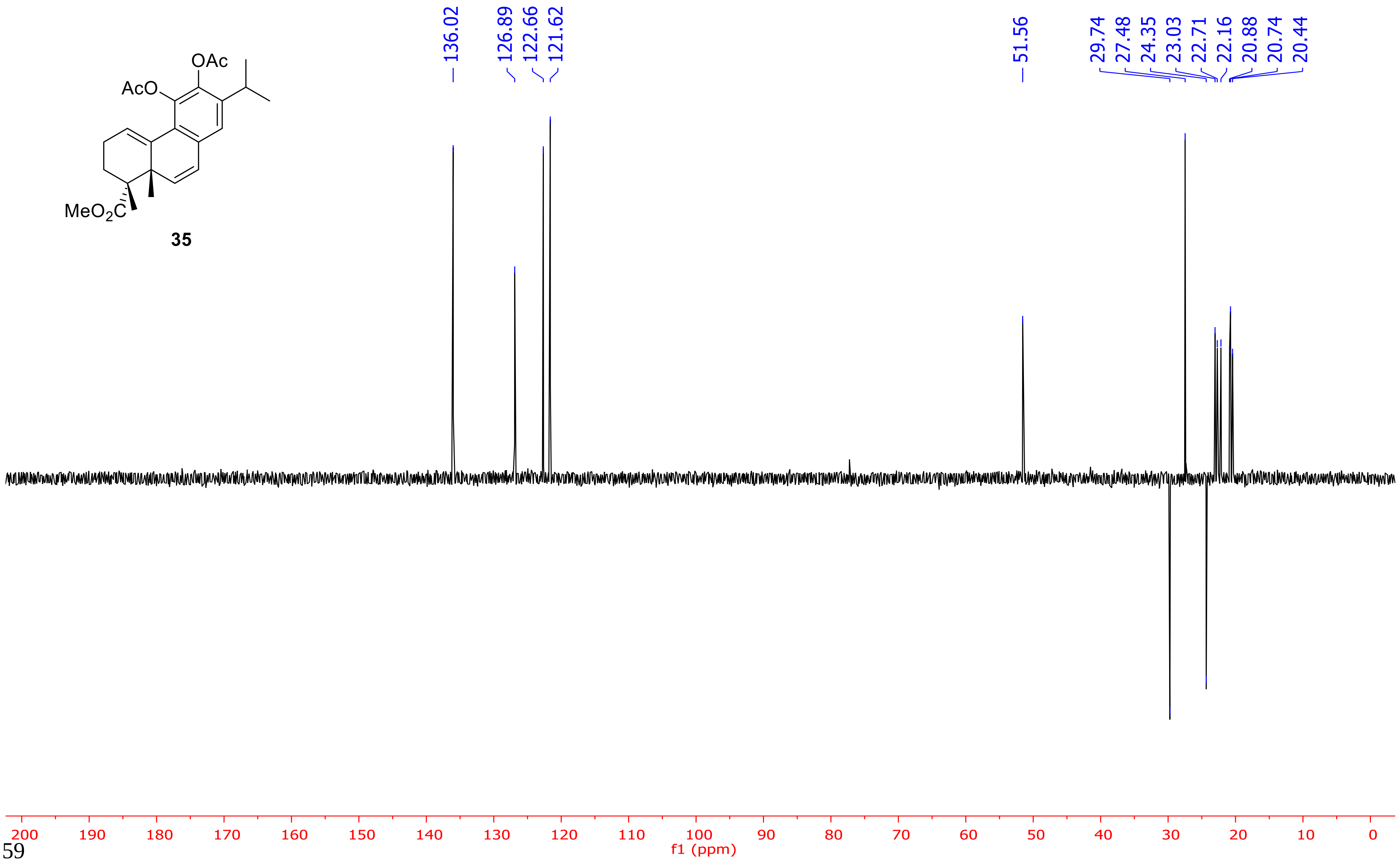
136.02
126.89
122.66
121.62
51.56
29.74
27.48
24.35
23.03
22.71
22.16
20.88
20.74
20.44
AcO
OAc
MeO2C
35
200
190
180
170
160
150
140
130
120
110
100
90
80
70
60
50
40
30
20
10
0
f1 (ppm)

59

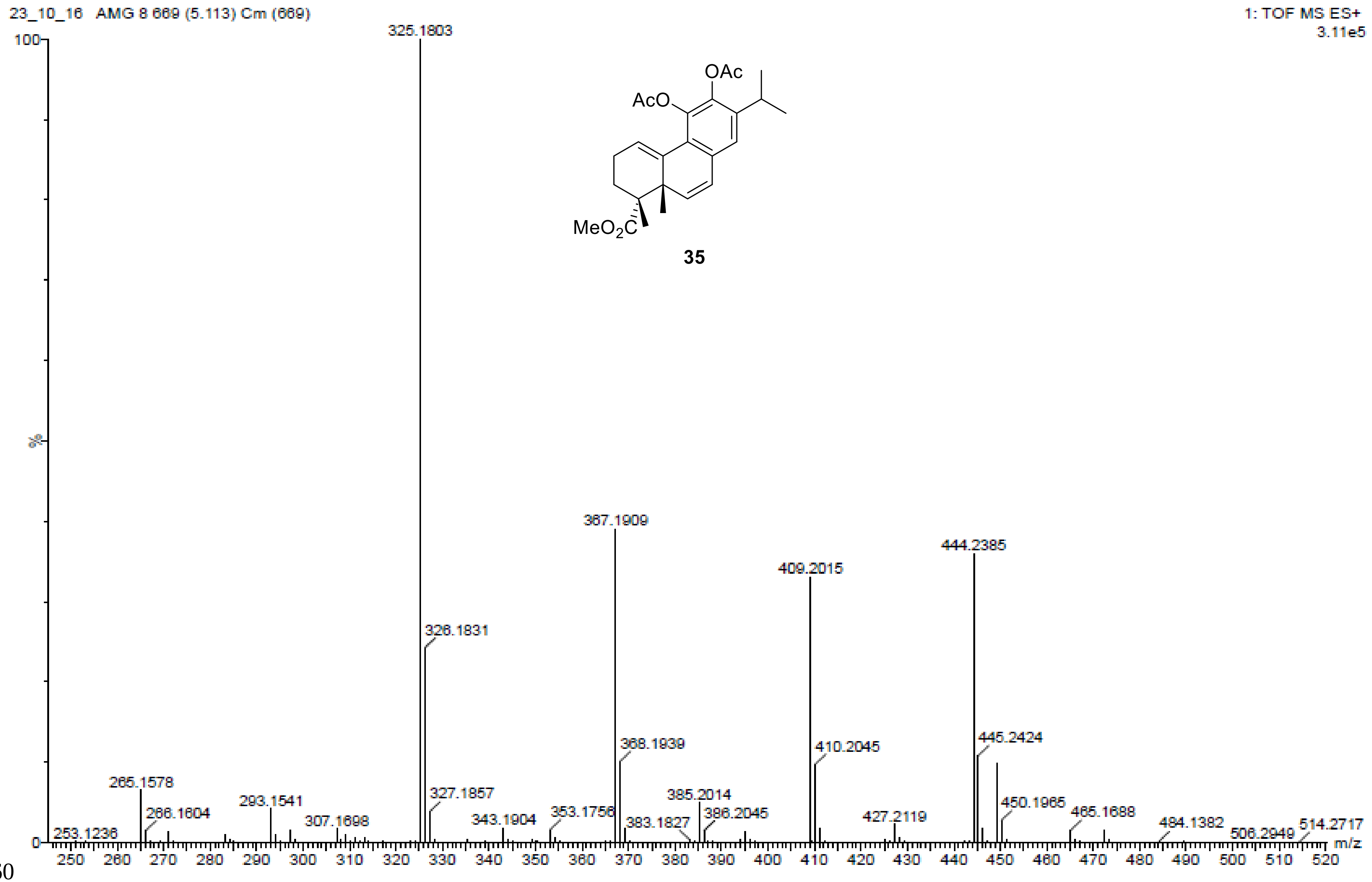

23_10_16 AMG 8 669 (5.113) Cm (669)
1: TOF MS ES+
3.11e5
OAc
AcO
MeO2C
325.1803
326.1831
327.1857
367.1909
368.1939
409.2015
410.2045
444.2385
445.2424
265.1578
266.1604
253.1236
293.1541
307.1698
343.1904
353.1756
383.1827
385.2014
386.2045
427.2119
450.1965
465.1688
484.1382
506.2949
514.2717
m/z

35

60

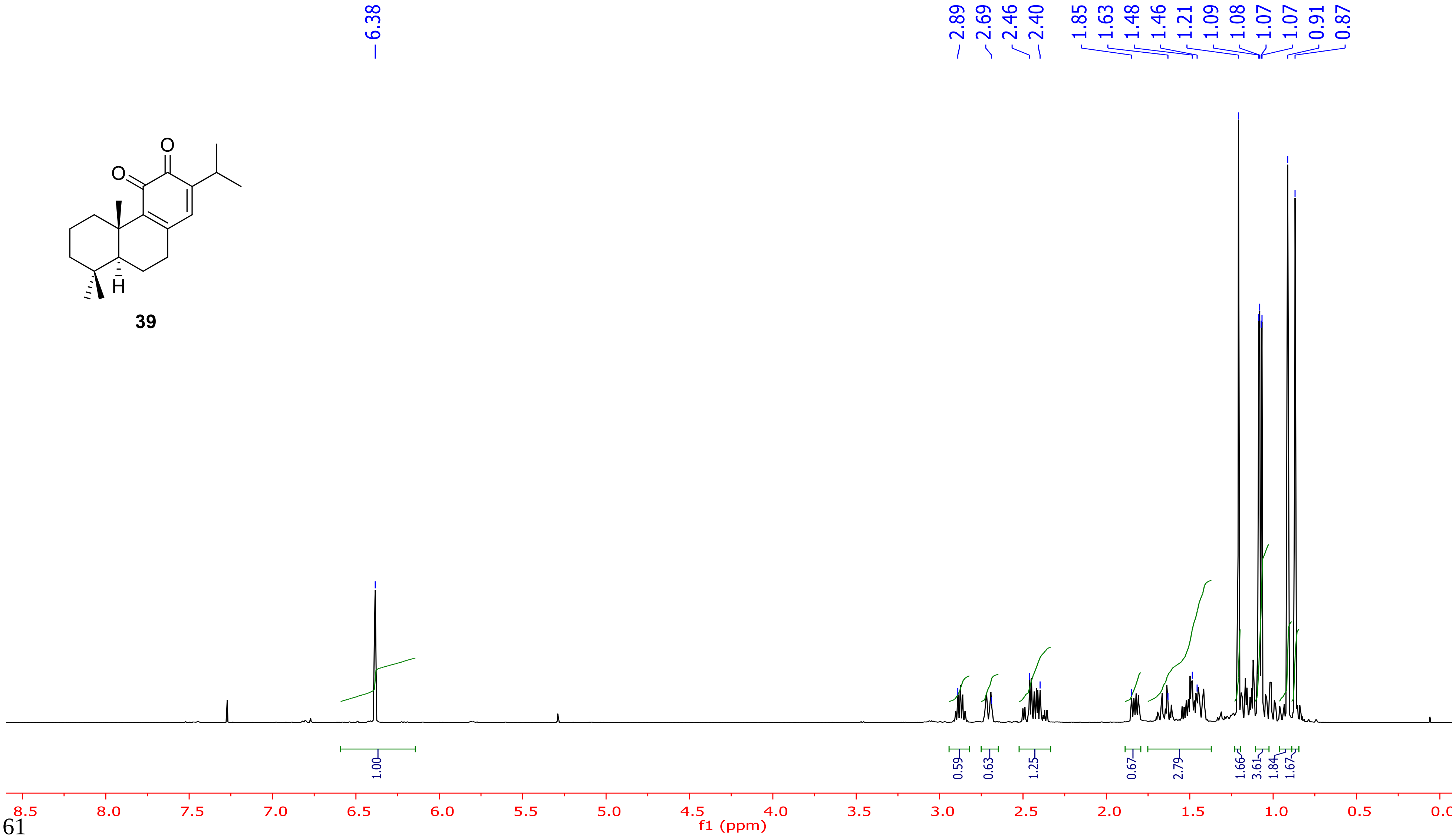
O
O
H
39
6.38
2.89
2.69
2.46
2.40
1.85
1.63
1.48
1.46
1.21
1.09
1.08
1.07
1.07
0.91
0.87
1.00
0.59
0.63
1.25
0.67
2.79
1.66
3.61
1.84
1.67
8.5
8.0
7.5
7.0
6.5
6.0
5.5
5.0
4.5
4.0
3.5
3.0
2.5
2.0
1.5
1.0
0.5
f1 (ppm)

61

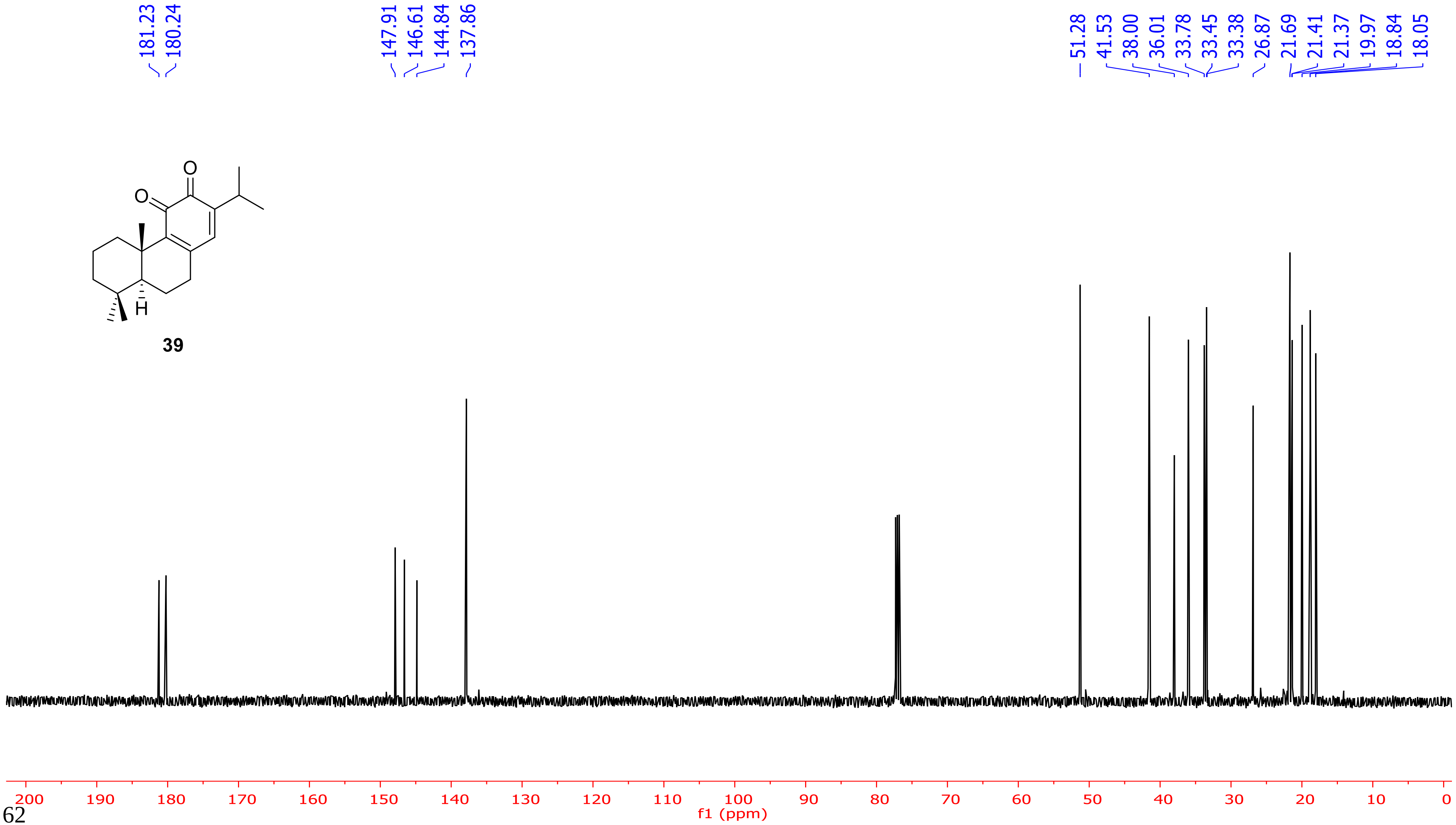

181.23
180.24
147.91
146.61
144.84
137.86
51.28
41.53
38.00
36.01
33.78
33.45
33.38
26.87
21.69
21.41
21.37
19.97
18.84
18.05
O
O
H
200
190
180
170
160
150
140
130
120
110
100
90
80
70
60
50
40
30
20
10
0
f1 (ppm)

39

62

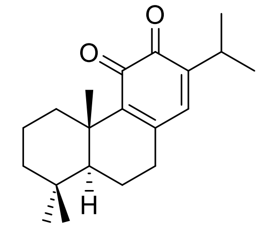

39

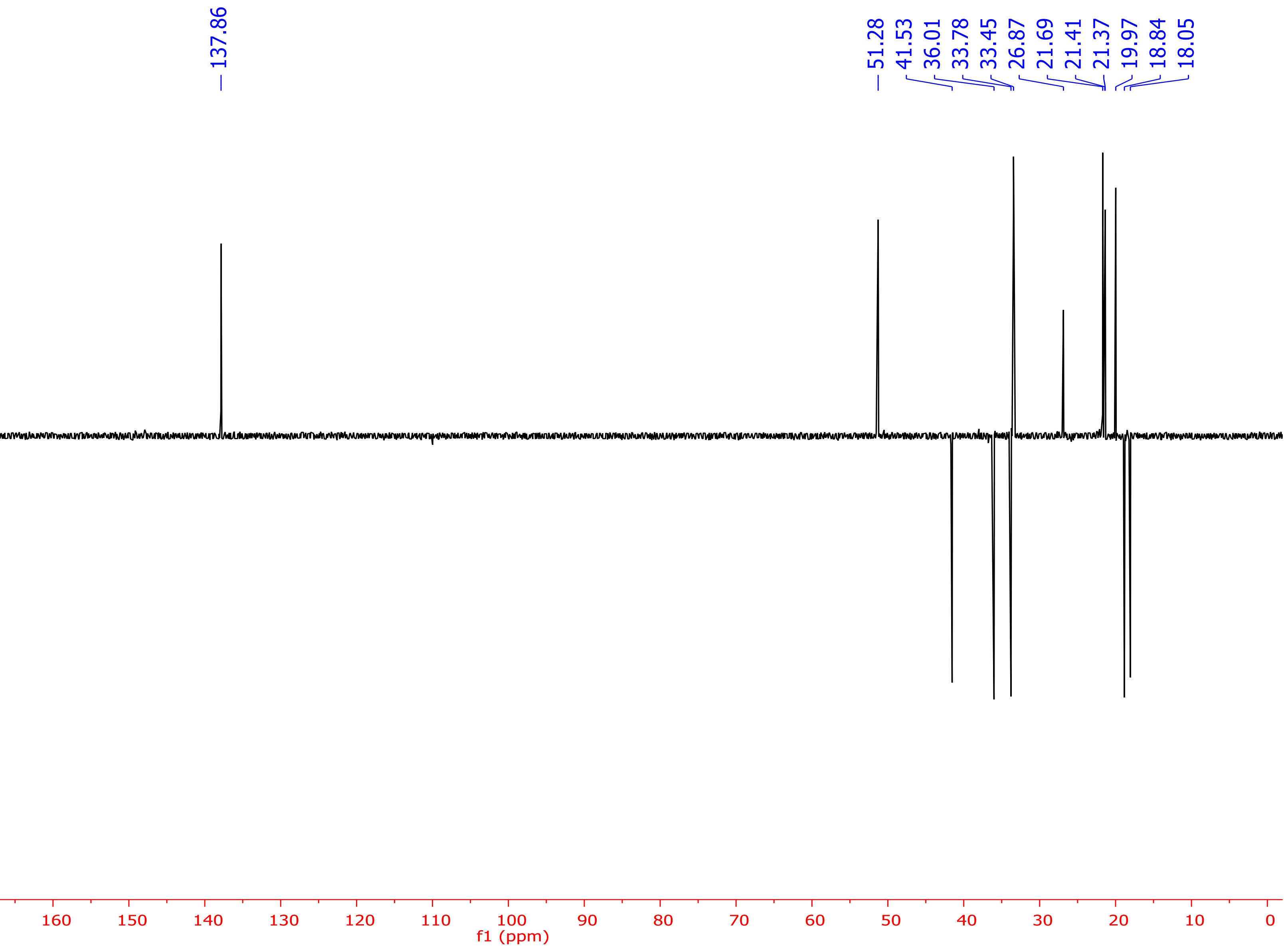
137.86
51.28
41.53
36.01
33.78
33.45
26.87
21.69
21.41
21.37
19.97
18.84
18.05
200
190
180
170
160
150
140
130
120
110
100
90
80
70
60
50
40
30
20
10
0
f1 (ppm)

63

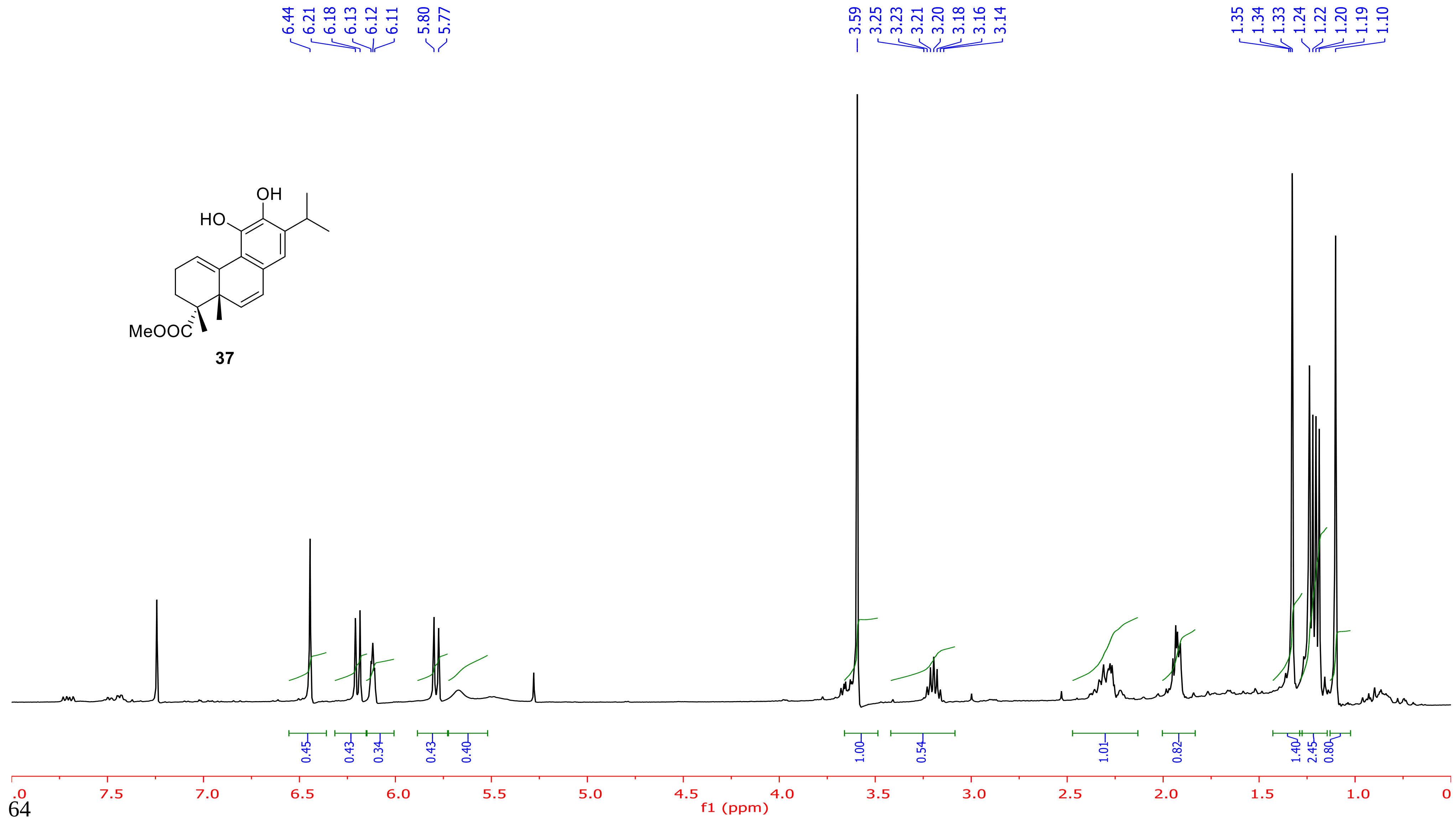
6.44
6.21
6.18
6.13
6.12
6.11
5.80
5.77
3.59
3.25
3.23
3.21
3.20
3.18
3.16
3.14
1.35
1.34
1.33
1.24
1.22
1.20
1.19
1.10
OH
HO
MeOOC
37
0.45
0.43
0.34
0.43
0.40
1.00
0.54
1.01
0.82
1.40
2.45
0.80
.0
7.5
7.0
6.5
6.0
5.5
5.0
4.5
4.0
3.5
3.0
2.5
2.0
1.5
1.0
0
f1 (ppm)
64

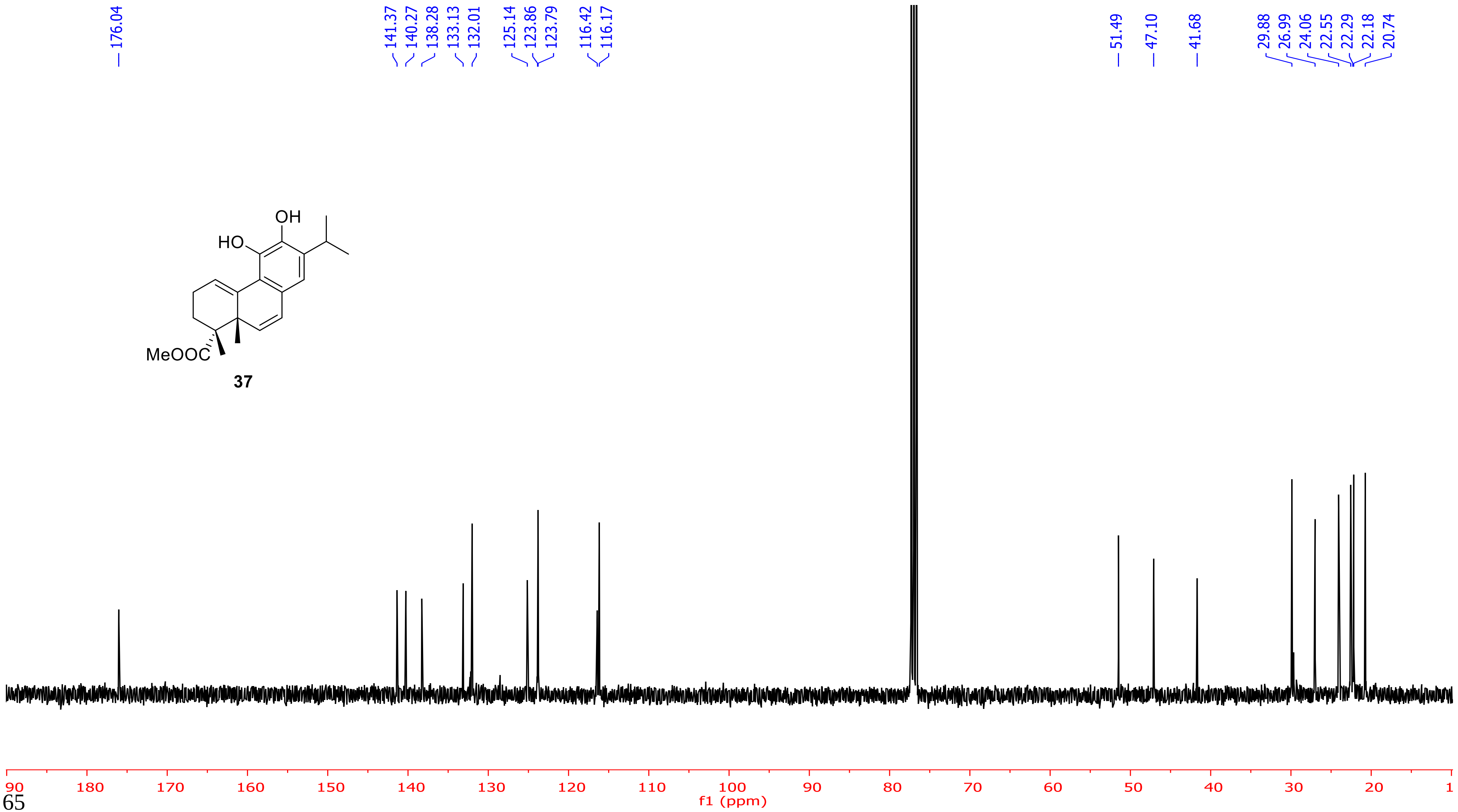
176.04
141.37
140.27
138.28
133.13
132.01
125.14
123.86
123.79
116.42
116.17
51.49
47.10
41.68
29.88
26.99
24.06
22.55
22.29
22.18
20.74
OH
HO
MeOOC
37
90
65
180
170
160
150
140
130
120
110
100
90
80
70
60
50
40
30
20
1
f1 (ppm)

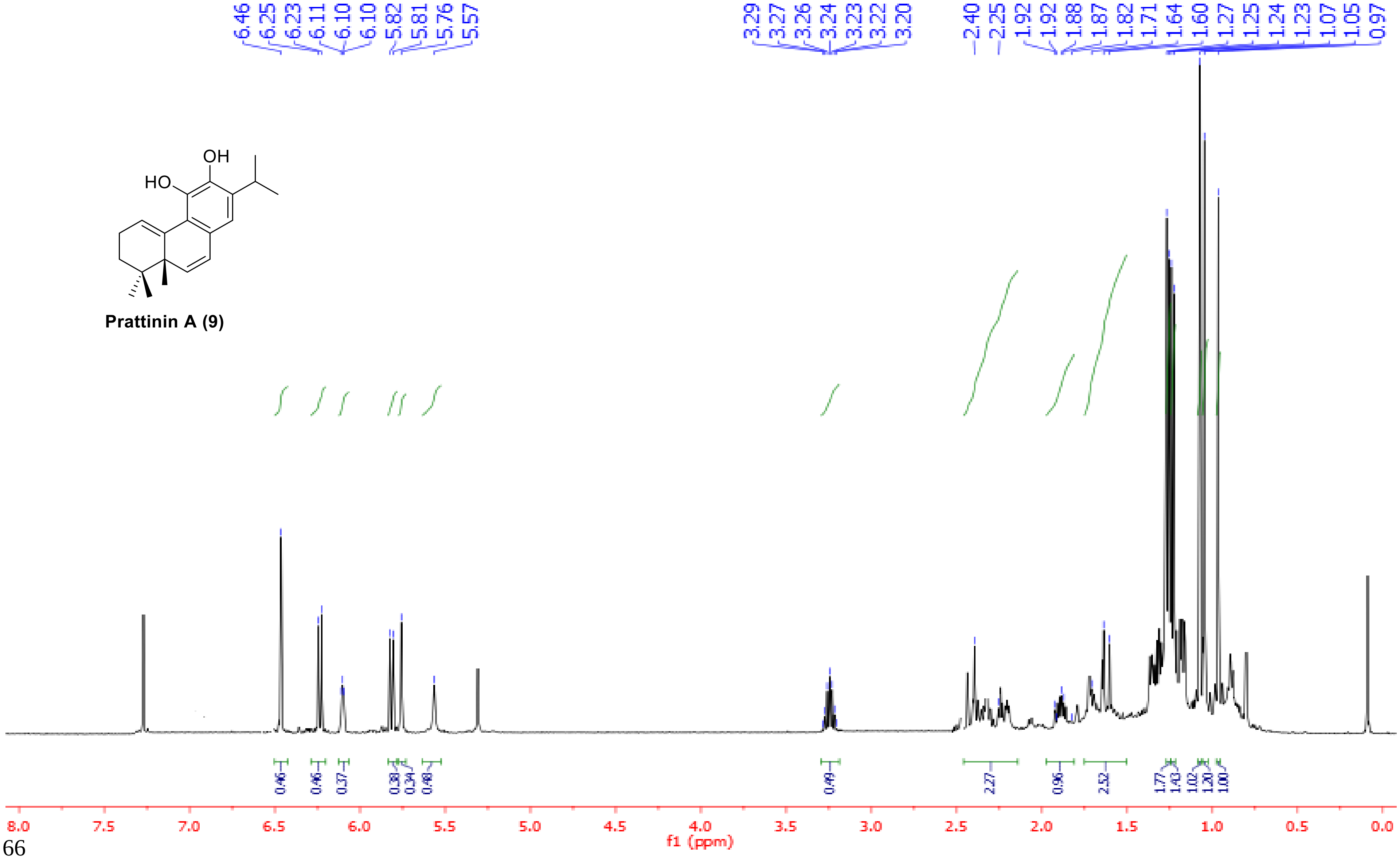
OH
HO
Prattinin A (9)
6.46 6.25 6.23 6.11 6.10 6.10 5.82 5.81 5.76 5.57
3.29 3.27 3.26 3.24 3.23 3.22 3.20
2.40 2.25 1.92 1.92 1.88 1.87 1.82 1.71 1.64 1.60 1.27 1.25 1.24 1.23 1.07 1.05 0.97
0.46 0.46 0.37 0.38 0.34 0.48 0.49 2.27 0.96 2.52 1.77 1.43 1.02 1.20 1.00
8.0 7.5 7.0 6.5 6.0 5.5 5.0 4.5 4.0 3.5 3.0 2.5 2.0 1.5 1.0 0.5 0.0
f1 (ppm)

66

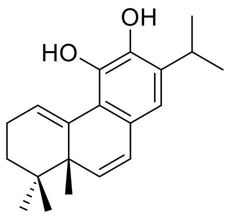

Prattinin A (9)

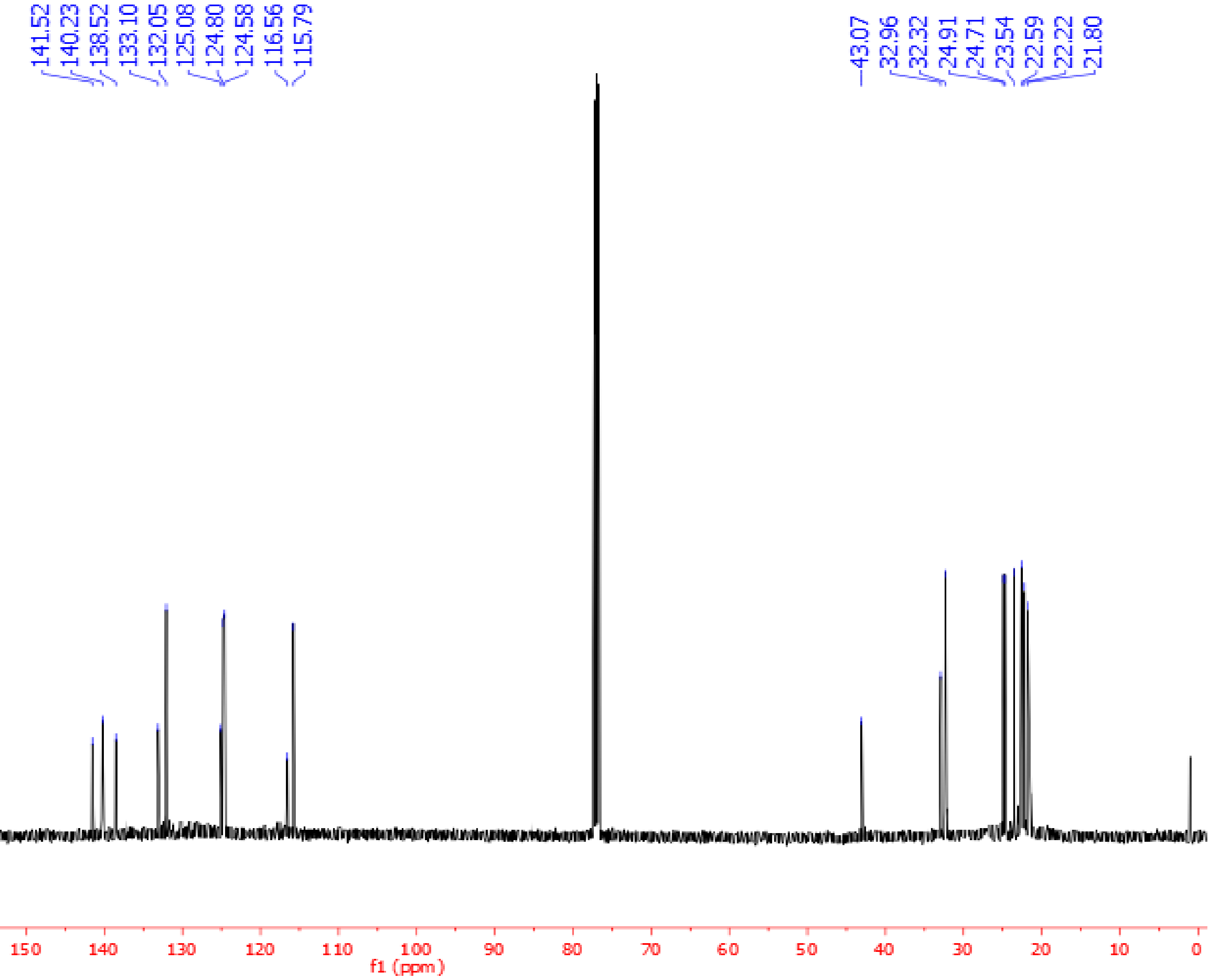

67

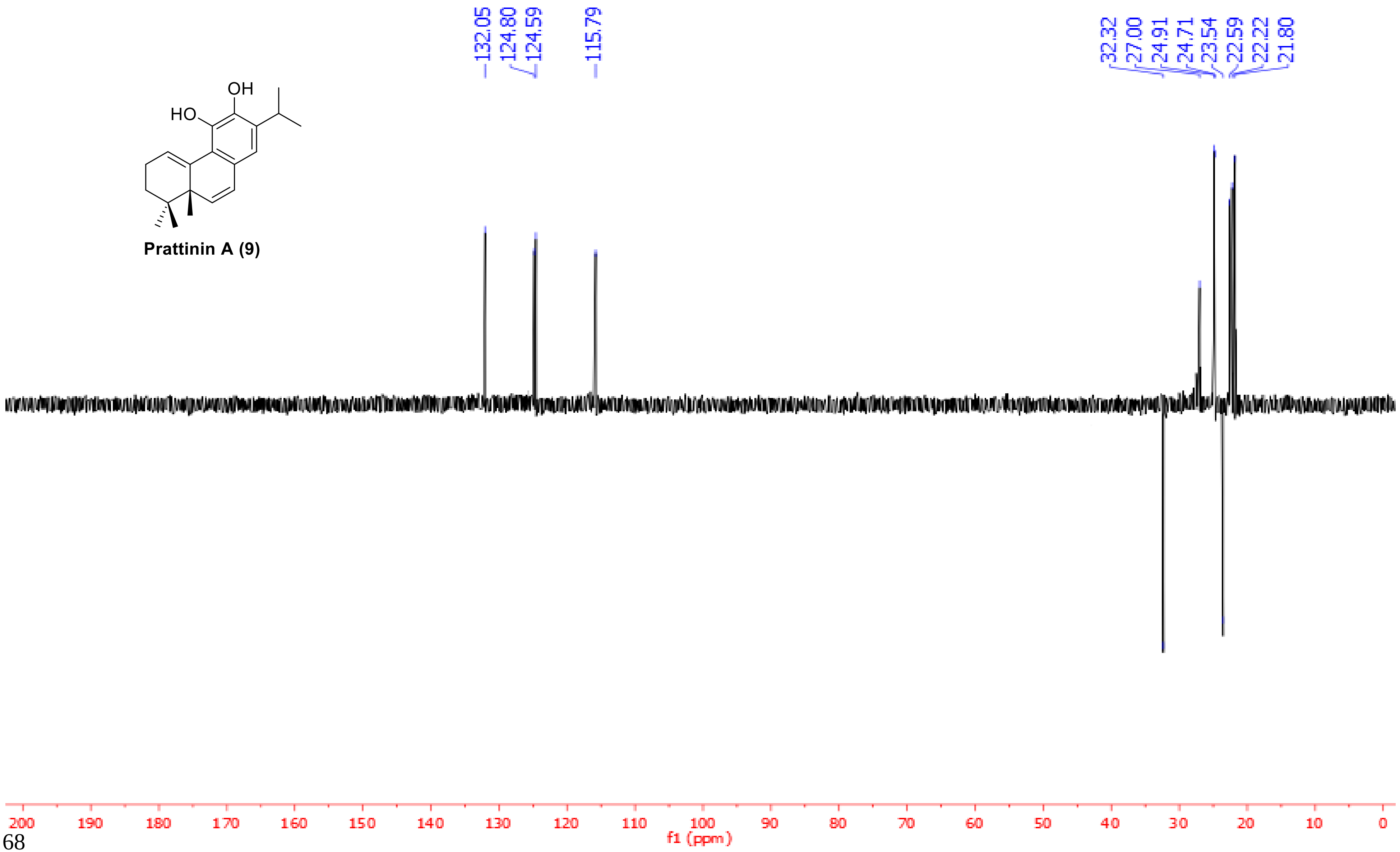

Prattinin A (9)

68

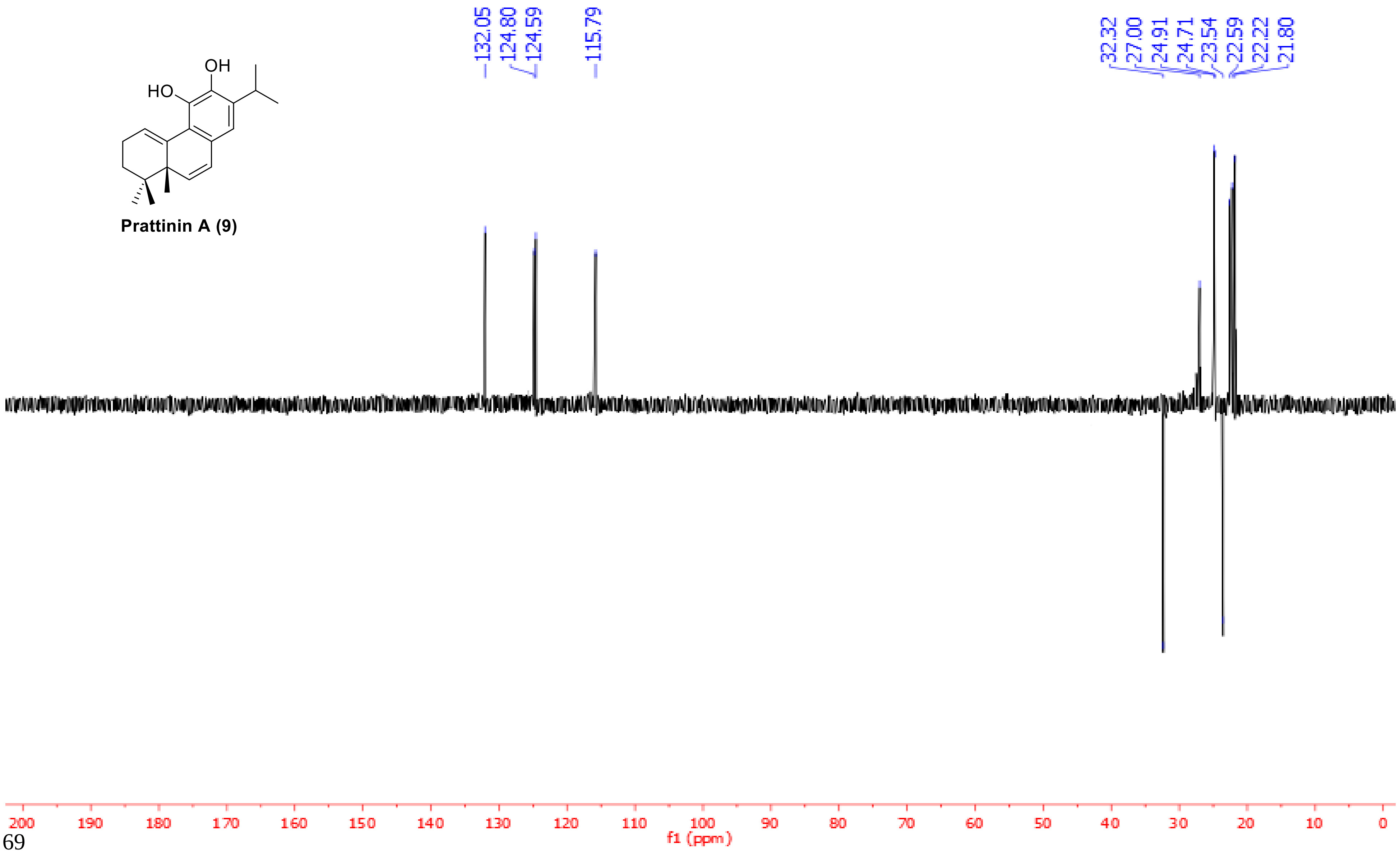
OH
HO
Prattinin A (9)
132.05
124.80
124.59
115.79
32.32
27.00
24.91
24.71
23.54
22.59
22.22
21.80
200
190
180
170
160
150
140
130
120
110
100
90
80
70
60
50
40
30
20
10
0
f1 (ppm)

69
